# Supplementary material for: Randomized, placebo controlled phase I trial of the safety, pharmacokinetics, pharmacodynamics and acceptability of a 90 day tenofovir plus levonorgestrel vaginal ring used continuously or cyclically in women: The CONRAD 138 study
Source: PLoS One. 2022 Oct 10;17(10):e0275794. doi: 10.1371/journal.pone.0275794 (PMC9550080; doi:10.1371/journal.pone.0275794)
Supplement: S1 Table — (DOCX) [file pone.0275794.s002.docx]

|  | | | | **Visit 1**  Screen | **Visit 2** | **Visit 3**  Base-line | **Visit 4** | **Visit 5**  24, 48, or 72h post insertion | **E2/P4** | **Visits**  **8,17,26** | **Visits**  **11,20,29** | **Visits**  **13,22,31** | **Visits**  **14,23** | **Visit 32**  48h, 72h, or 5d post removal | **Follow Up Contact** |
| --- | --- | --- | --- | --- | --- | --- | --- | --- | --- | --- | --- | --- | --- | --- | --- |
| Informed consent | | | | ✓ |  |  |  |  |  |  |  |  |  |  |  |
| Ask about AEs, CMs, and Menses | | | |  |  |  | ✓ | ✓ | ✓ | ✓ | ✓ | ✓ | ✓ | ✓ | ✓ |
| Urine | | Pregnancy test | | ✓ | ✓ | ✓ | ✓ |  |  |  |  |  | ✓ | ✓ |  |
| Blood | | HSV-2, HIV-1, and HBsAG | | ✓ |  |  |  |  |  |  |  |  |  | ✓  (HIV-1 Only) |  |
|  |  | Safety (CBC, chemistries, lipids - fasting) | | ✓ |  |  |  |  |  |  |  | ✓  (V31) |  |  |  |
|  |  | PK TFV | |  |  |  | ✓  (Pre,  8h) | ✓ |  | ✓ | ✓ | ✓ | ✓ | ✓ |  |
|  |  | PK LNG/SHBG | |  |  |  | ✓  (Pre,1,2,4,8h) | ✓ |  | ✓ | ✓ | ✓ | ✓ | ✓ |  |
|  |  | PD LNG (P4 and Estradiol) | |  | ✓^1^ |  | ✓ |  | ✓ | ✓ | ✓ | ✓ | ✓ |  |  |
| Pelvic Exam and Genital Samples | Exam | | Pelvic Exam | ✓ | (✓) | ✓ | ✓ | ✓ | ✓ | ✓ | ✓ | ✓ | ✓ | ✓ | (✓) |
|  |  |  | Gram Stain | ✓ | (✓) | (✓) | (✓) | (✓) | (✓) | (✓) | (✓) | (✓) | (✓) | (✓) | (✓) |
|  | PK | | CV Tissue – Biopsy TFV, TFV-DP |  |  |  |  | ✓ |  |  |  | ✓  (V31) |  | ✓ |  |
|  |  |  | CV Fluid (swab) TFV |  |  |  | ✓  (2,8h) | ✓ |  | ✓ | ✓ |  | ✓ | ✓ |  |
|  |  |  | Rectal Fluid (sponge) TFV |  |  |  |  | ✓ |  |  | ✓ |  |  | ✓ |  |
|  | Safety | | CV Tissue – Biopsy Lymphocytes |  |  | ✓ |  |  |  |  |  | ✓  (V31) |  |  |  |
|  |  |  | CV Fluid (CVL) Soluble Secreted Proteins |  |  |  | ✓ |  |  |  | ✓  (V29) |  |  |  |  |
|  |  |  | CV Fluid (swab) Microflora |  |  |  | ✓ |  |  |  |  | ✓ |  |  |  |
|  | | PD | CV Tissue – Biopsy [EVMS site only]  HIV-1 & HSV-2 Infectivity |  |  | ✓ |  |  |  |  |  | ✓  (V31) |  |  |  |
|  |  |  | CV Fluid  anti-HIV-1/anti-HSV-2 |  |  |  | ✓ |  |  |  | ✓  (V11,29) |  |  |  |  |
|  |  |  | Cervical Mucus  Quality/Sperm Migration |  |  |  |  |  | (✓) | (✓) | (✓) | (✓) | (✓) |  |  |
|  |  |  | Rectal Fluid  anti-HIV-1/anti-HSV-2 |  |  |  | ✓ |  |  |  | ✓  (V29) |  |  |  |  |
| Randomization to treatment and or time point post-insertion/removal | | | |  |  | ✓ |  |  | ✓  (V27) | ✓  (V26) |  |  |  |  |  |
| IVR Dispensation/Insertion | | | |  |  |  | ✓ |  |  |  |  |  | ✓ |  |  |
| IVR Removal (Complete IVR processing at V31) | | | |  |  |  |  |  |  |  |  | ✓^7^ |  |  |  |
| Acceptability/Psychosocial Questionnaire | | | |  |  | ✓ |  |  |  |  |  | ✓  (V13,31) |  |  |  |
| IDI (completed by a subset of participants) | | | |  |  |  |  |  | ✓ |  |  | ✓  (V31) |  | ✓ |  |
| Study Exit | | | |  |  |  |  |  |  |  |  |  |  |  | ✓ |

AE=adverse event; CBC=complete blood count; CM=concomitant medication; CT=Chlamydia trachomatis; CV=cervicovaginal; CVL=cervicovaginal lavage; EVMS=Eastern Virginia Medical School; GC=Neisseria gonorrhea; h=hour; HBsAg= hepatitis B surface antigen; HIV=human immunodeficiency virus; HSV‑herpes simplex virus; IDI=in-depth interview; IVR=intravaginal ring; LNG=levonorgestrel; P4=serum progesterone; PD=pharmacodynamics; PK=pharmacokinetics; Pre=pre-IVR insertion; TFV=tenofovir; TFV‑DP=tenofovir‑diphosphate ; TV=Trichomonas vaginalis; V=visit.

(✓) = if indicated

**NOTE:** directed physical exam, pelvic exam (possibly including removal of the IVR, per investigator discretion), dipstick/microscopy/culture and wet mount/pH were performed at any visit per investigator discretion

**Supplemental Table 1. Schedule of Evaluations**
