# Supplementary material for: Randomized, placebo controlled phase I trial of the safety, pharmacokinetics, pharmacodynamics and acceptability of a 90 day tenofovir plus levonorgestrel vaginal ring used continuously or cyclically in women: The CONRAD 138 study
Source: PLoS One. 2022 Oct 10;17(10):e0275794. doi: 10.1371/journal.pone.0275794 (PMC9550080; doi:10.1371/journal.pone.0275794)
Supplement: S2 Table — (DOCX) [file pone.0275794.s003.docx]

|  | TFV/LNG continuous | | | | TFV/LNG Interrupted | | | | Placebo Continuous | | | | Placebo Interrupted | | | |
| --- | --- | --- | --- | --- | --- | --- | --- | --- | --- | --- | --- | --- | --- | --- | --- | --- |
|  | N | Mean | STD | Median | N | Mean | STD | Median | N | Mean | STD | Median | N | Mean | STD | Median |
| CD4* | 2 | 53.4 | 13.1 | 53.4 | 6 | 9.5 | 5.8 | 7.3 | 1 | 34.4 | . | 34.4 | 1 | 38.1 | . | 38.1 |
| CCR5* | 2 | 0 | 0 | 0 | 6 | 0 | 0 | 0 | 1 | 0 | . | 0 | 1 | 0 | . | 0 |
| CD4* | 2 | 209.6 | 115.4 | 209.6 | 7 | 56.8 | 51.82 | 42.7 | 1 | 137.6 | . | 137.6 | 1 | 105.6 | . | 105.6 |
| CCR5* | 2 | 40 | 11.3 | 40 | 8 | 15.7 | 14.25 | 8 | 1 | 51.2 | . | 51.2 | 1 | 3.2 | . | 3.2 |

*CD4 and CCR5 measured in a subset of ectocervical biopsies from the EVMS site only, processed using cryopreservation, as in methods

**Supplemental Table 2. CD4 and CCR5 positive cell density in cryopreserved samples.**
