# Supplementary material for: Randomized, placebo controlled phase I trial of the safety, pharmacokinetics, pharmacodynamics and acceptability of a 90 day tenofovir plus levonorgestrel vaginal ring used continuously or cyclically in women: The CONRAD 138 study
Source: PLoS One. 2022 Oct 10;17(10):e0275794. doi: 10.1371/journal.pone.0275794 (PMC9550080; doi:10.1371/journal.pone.0275794)
Supplement: S3 Table — (DOCX) [file pone.0275794.s004.docx]

| Sample | Parameter (units) | Time Period | PK Parameter | Statistic | TFV/LNG Continuous (n = 18) | TFV/LNG Interrupted (n = 17) |
| --- | --- | --- | --- | --- | --- | --- |
| Plasma | TFV (ng/mL) | Post-insertion (0 – 72 hours) | AUC_0-24_ (h*ng/mL) | Estimate (SE) | 4.82 (0.59) | 5.08 (0.87) |
|  |  |  | AUC_0-48_ (h*ng/mL) | Estimate (SE) | 25.94 (5.62) | 28.97 (5.74) |
|  |  |  | AUC_0-72_ (h*ng/mL) | Estimate (SE) | 59.84 (11.47) | 61.99 (11.05) |
|  |  |  | C_max_ (ng/mL) | Estimate (SE) | 1.42 (0.28) | 1.59 (0.44) |
|  |  |  | T_max_ (h) | Estimate | 72 | 48 |
|  |  | Post Removal | T_1/2_ (h) | Estimate | NC | NC |
|  |  |  |  |  |  |  |
| Vaginal Fluid | TFV (ng/mg) | Post-insertion (0 – 72 hours) | AUC_0-24_ (h*ng/mg) | Estimate (SE) | 4,651.46 (547.71) | 4,475.16 (624.67) |
|  |  |  | AUC_0-48_ (h*ng/mg) | Estimate (SE) | 31,865.29 (5,493.36) | 20,410.89 (2,357.98) |
|  |  |  | AUC_0-72_ (h*ng/mg) | Estimate (SE) | 70,999.10 (11,195.40) | 52.138.38 (6,519.34) |
|  |  |  | C_max_ (ng/mg) | Estimate (SE) | 1,756.35 (443.59) | 1,805.81 (435.64) |
|  |  |  | T_max_ (h) | Estimate | 48 | 72 |
|  |  | Post Removal | T_1/2_ (h) | Estimate | NC | NC |
|  |  |  |  |  |  |  |
| Rectal Fluid | TFV (ng/mg) | Post-insertion (0 – 72 hours) | AUC_0-24_ (h*ng/mg) | Estimate (SE) | 13.16 (19.82) | 10.00 (10.47) |
|  |  |  | AUC_0-48_ (h*ng/mg) | Estimate (SE) | 27.91 (19.83) | 64.03 (38.71) |
|  |  |  | AUC_0-72_ (h*ng/mg) | Estimate (SE) | 38.52 (20.39) | 114.06 (75.41) |
|  |  |  | C_max_ (ng/mg) | Estimate (SE) | 1.10 (0.83) | 3.67 (3.11) |
|  |  |  | T_max_ (h) | Estimate | 24 | 48 |
|  |  | Post Removal | T_1/2_ (h) | Estimate | NC | NC |
|  |  |  |  |  |  |  |
| Vaginal Tissue | TFV (ng/mg) | Post-insertion (0 – 72 hours) | AUC_0-24_ (h*ng/mg) | Estimate (SE) | 454.63 (390.30) | 215.86 (185.37) |
|  |  |  | AUC_0-4_ (h*ng/mg) | Estimate (SE) | 1,140.13 (399.91) | 1,136.19 (338.38) |
|  |  |  | AUC_0-72_ (h*ng/mg) | Estimate (SE) | 3,166.65 (1,116.38) | 3,008.59 (664.60) |
|  |  |  | C_max_ (ng/mg) | Estimate (SE) | 149.64 (85.94) | 97.33 (24.55) |
|  |  |  | T_max_ (h) | Estimate | 72 | 72 |
|  |  | Post Removal | T_1/2_ (h) | Estimate | NC | NC |
|  |  |  |  |  |  |  |
|  | TFV-DP (fmol/mg) | Post-insertion (0 – 72 hours) | AUC_0-24_ (h*fmol/mg) | Estimate (SE) | 5,667.29 (3,201.27) | 1,367.34 (789.03) |
|  |  |  | AUC_0-4_ (h*fmol/mg) | Estimate (SE) | 21,259.99 (6,743.04) | 22,904.51 (14,279.57) |
|  |  |  | AUC_0-72_ (h*fmol/mg) | Estimate (SE) | 96,531.40 (33,149.49) | 113,694.17 (50,225.17) |
|  |  |  | C_max_ (fmol/mg) | Estimate (SE) | 5,445.50 (2,565.47) | 5,884.99 (3,444.81) |
|  |  |  | T_max_ (h) | Estimate | 72 | 72 |
|  |  | Post Removal | T_1/2_ (h) | Estimate | NC | NC |
|  |  |  |  |  |  |  |
| Serum | LNG (pg/mL) | Post-insertion (0-72 hours) | AUC_0-8_ (h*pg/mL) | Mean (SD) | 1,606.3 (522.27) | 1,812.7 (651.49) |
|  |  |  |  | CV% | 32.51 | 35.94 |
|  |  |  |  | Geometric Mean | 1522.8 | 1708.6 |
|  |  |  |  | Geometric CV (%) | 35.61 | 36.54 |
|  |  |  |  | Median | 1,607.3 | 1,700.5 |
|  |  |  |  | Min., Max. | 705.2, 2748.7 | 902.5, 3,174.0 |
|  |  |  | AUC_0-24_ (h*pg/mL) | Estimate (SE) | 6,779.56 (546.33) | 7,076.27 (561.21) |
|  |  |  | AUC_0-48_ (h*pg/mL) | Estimate (SE) | 16,303 (1,539.40) | 15,464.27 (1,380.91) |
|  |  |  | AUC_0-72_ (h*pg/mL) | Estimate (SE) | 24,136.59 (2,258.80) | 22,949.07 (1,832.95) |
|  |  |  | C_max_ (pg/mL) | Estimate (SE) | 407.40 (75.97) | 368.67 (62.17) |
|  |  |  | T_max_ (h) | Estimate | 48 | 24 |
|  |  | Post Removal | T_1/2_ (h) | Estimate | 25.74 | NC |
|  |  |  |  |  |  |  |
|  | SHBG (ug/mL) | Post-insertion (0-72 hours) | AUC_0-8_ (h*ug/mL) | Mean (SD) | 78.9 (50.95) | 69.9 (24.41) |
|  |  |  |  | CV% | 64.61 | 34.91 |
|  |  |  |  | Geometric Mean | 65.9 | 66.6 |
|  |  |  |  | Geometric CV (%) | 66.48 | 31.60 |
|  |  |  |  | Median | 51.1 | 60.2 |
|  |  |  |  | Min., Max. | 27.6, 179.1 | 44.7, 125.2 |
|  |  |  | AUC_0-24_ (h*ug/mL) | Estimate (SE) | 267.43 (31.88) | 214.81 (15.50) |
|  |  |  | AUC_0-48_ (h*ug/mL) | Estimate (SE) | 556.57 (77.94) | 441.60 (45.01) |
|  |  |  | AUC_0-72_ (h*ug/mL) | Estimate (SE) | 754.17 (100.08) | 637.27 (69.34) |
|  |  |  | C_max_ (ug/mL) | Estimate (SE) | 13.51 (3.39) | 9.61 (2.50) |
|  |  |  | T_max_ (h) | Estimate | 24 | 48 |
|  |  | Post Removal | T_1/2_ (h) | Estimate | NC | NC |

SE = Standard Error, SD = Standard Deviation, CV% = Coefficient of variation percent, Min. = Minimum, Max. = Maximum, AUC = area under the curve; Cmax = maximum concentration; LNG = levonorgestrel; NC = not calculated; PK = pharmacokinetic; SHBG = sex hormone binding globulin; TFV = tenofovir; TFV-DP = tenofovir diphosphate; T1/2, apparent terminal half-life; Tmax, time to maximum concentration

**Supplemental Table 3. Pharmacokinetic parameters**
