# Supplementary material for: Randomized, placebo controlled phase I trial of the safety, pharmacokinetics, pharmacodynamics and acceptability of a 90 day tenofovir plus levonorgestrel vaginal ring used continuously or cyclically in women: The CONRAD 138 study
Source: PLoS One. 2022 Oct 10;17(10):e0275794. doi: 10.1371/journal.pone.0275794 (PMC9550080; doi:10.1371/journal.pone.0275794)
Supplement: S4 Table — (DOCX) [file pone.0275794.s005.docx]

Supplemental Table 4. Comparison of paired changes from baseline in percent HSV-2 inhibition by vaginal and rectal fluids using Vero cell assay

| IVR Treatment, Dosing Regimen and Fluid | V4 Baseline Pre IVR Insertion | | | | Month 1 (V11) | | | | P ( V11 – V4) | End of Treatment (V29) | | | | P (V29 – V4) |
| --- | --- | --- | --- | --- | --- | --- | --- | --- | --- | --- | --- | --- | --- | --- |
| VAGINAL FLUID | N | Mean | STD | Median | N | Mean | STD | Median |  | N | Mean | STD | Median |  |
| TFV/LNG CONTINUOUS | 11 | 14.03 | 42.5 | 4.55 | 10 | 49.02 | 41.63 | 46.44 | 0.12 | 12 | 23.74 | 29.31 | 17.36 | 0.02 |
| TFV/LNG INTERRUPTED | 11 | 19.09 | 32.05 | 15.38 | 11 | 11.59 | 28.59 | 11.54 | 1.00 | 12 | 0.22 | 21.82 | 1.51 | 0.34 |
| PLACEBO CONTINUOUS | 3 | 11.38 | 7.01 | 10.58 | 3 | 12.53 | 2.56 | 13.54 | 1.00 | 4 | 17.3 | 10.08 | 16.36 | 1.00 |
| PLACEBO INTERRUPTED | 4 | -7.55 | 27.57 | -15.36 | 4 | 31.33 | 36.7 | 20.73 | 0.12 | 5 | 14.01 | 22.76 | 10.91 | 0.15 |
| RECTAL FLUID | V4 Baseline Pre IVR Insertion | | | | Month 1 (V11) not assessed for rectal fluid | | | | | End of Treatment (V29) | | | | P (V29 – V4) |
|  | N | Mean | STD | Median |  |  |  |  |  | N | Mean | STD | Median |  |
| TFV/LNG CONTINUOUS | 11 | -10.74 | 25.83 | -9.89 |  |  |  |  |  | 13 | 18.47 | 29.59 | 8.24 | 0.05 |
| TFV/LNG INTERRUPTED | 10 | -1.14 | 41.57 | 12.39 |  |  |  |  |  | 10 | 12.04 | 29.29 | 12.1 | 0.31 |
| PLACEBO CONTINUOUS | 2 | 5.84 | 21.58 | 5.84 |  |  |  |  |  | 5 | 0.56 | 21.01 | -4.4 | 0.13 |
| PLACEBO INTERRUPTED | 3 | -5.29 | 32.29 | 6.25 |  |  |  |  |  | 3 | 24.15 | 14 | 27.06 | 0.34 |
