# Supplementary material for: Randomized, placebo controlled phase I trial of the safety, pharmacokinetics, pharmacodynamics and acceptability of a 90 day tenofovir plus levonorgestrel vaginal ring used continuously or cyclically in women: The CONRAD 138 study
Source: PLoS One. 2022 Oct 10;17(10):e0275794. doi: 10.1371/journal.pone.0275794 (PMC9550080; doi:10.1371/journal.pone.0275794)
Supplement: S1 File — (DOCX) [file pone.0275794.s006.docx]

**SUPPLEMENTAL METHODS**

**Density and phenotype of immune cells in ectocervical tissue**

For cryopreservation, the ectocervical tissues were embedded in Optimal Cutting Temperature (O.C.T.) compound (Tissue Tek, Torrance, CA) at -20°C immediately. Five to six micron sections were cut using a cryostat microtome (Tissue-Tek, USA) and stored at -20°C until processed for staining for CD4 and CCR5. In brief, the slides were brought to room temperature and washed with phosphate buffered saline (PBS). The sections were then fixed in cold acetone for 15 minutes. After subsequent washings with PBS the tissue sections were incubated for 30 minutes with specific serum protein to block non-specific binding. The tissues were then treated with CD4 (1:20, DAKO) or CCR5 (1:100, R&D) and placed in a refrigerator overnight. The following day, steps followed were similar to that processed for paraffin embedded sections.

The antigens were detected using AEC chromogen – substrate kit (SkyTek labs, Mississauga, Ontario, Canada) and mounted with Accergyl mounting media (Accurate Chemicals, NY, USA). Cell phenotype was identified using specific monoclonal antibodies against CD45, CD3, CD8, (Leica Biosystems, Wetzlar Germany) and HLA-DR (Santa Cruz Biotechnology, Dallas, TX) (in paraffin embedded tissues) and CCR5 (R&D Systems, Minneapolis, MN) and CD4 (Agilent, Santa Clara, CA) (in cryopreserved embedded tissues). Positive stained cells were counted under the microscope (Nikon E-800). In brief, 5 - 6 fields were randomly selected using a Nikon E800 microscope from each section and these images were captured using a CCD camera (Spot Camera, Diagnostic Instruments MI, USA). Cell density was expressed as the mean of the counts in 5 – 6 fields in cells/mm^2^.

**Secreted soluble proteins from the CV mucosa**

The CVL was centrifuged at 4^o^C for 10 min at 800 g, and frozen at -80 ⁰C and transported to the laboratory of Genital Tract Biology at Brigham and Women’s Hospital (RF) where the supernatant was analyzed for safety biomarkers. ELISA was used to measure levels of the secretory leukocyte protease inhibitor (SLPI) (R&D Systems, Minneapolis, MN) and β-defensin- 2 (BD2) (Phoenix Pharmaceuticals, Burlingame, CA) using Victor2 reader (Perkin Elmer Life Sciences, Boston, MA). Samples were screened at 50-fold dilution for SLPI and 100-fold dilution for BD2, and all samples showing levels above the assay detection range were additionally diluted to obtain accurate measurements. Interleukin (IL)-1α, IL-6, IL-10, granulocyte-macrophage colony-stimulating factor (GM-CSF), macrophage inflammatory protein (MIP)-1α , RANTES and tumor necrosis factor (TNF)-α, and were measured in undiluted CVLs by a multiplex electrochemiluminescence (ECL) assay (Meso Scale Discovery (MSD), Gaithersburg, MD). Interferon gamma-induced protein 10 (IP-10), IL-8 (2x and 20x) and IL-1receptor antagonist (RA) were measured by separate single-plex MSD assays because of detection antibody incompatibility (IP-10), higher dilution required (20-fold for IL-8 and 100-fold or 1000-fold for IL-1RA). IL-1-RA was diluted 50x and 100x. All measurements were performed in duplicate. A split quality control pool prepared from CVLs was tested on each plate showing inter-plate coefficient variation (CV) of 10% for SLPI, 11.8% for BD2, between 4.7% and 8.5% for the markers on the MSD multiplex plates, 4.5% for IP-10, 3.1% for IL-8 and 8.5% for IL-1RA.

**Tenofovir pharmacokinetics**

Assay lower limits of quantitation (LLOQ) were as follows: plasma TFV, 0.31 ng/mL; vaginal fluid (VF) (swab) TFV, 0.625 ng/Dacron swab; rectal fluid (RF) (sponge) TFV, 1.25 ng/sponge; vaginal tissue TFV, 0.05 ng/sample. Results were normalized to net collection device weight (swab or sponge) or weight of tissue analyzed, and TFV concentrations in these matrices were reported as ng/mg. TFV-DP was measured using a previously described indirect approach, in which TFV was quantitated following isolation of TFV-DP from homogenized tissue lysates and enzymatic conversion to the TFV molecule [1]. The assay LLOQ for TFV-DP in tissue was 5 fmol/sample, and drug concentrations were normalized to the amount of tissue analyzed [2]. All assays were validated in accordance with FDA, Guidance for Industry: Bioanalytical Method Validation recommendations [3]. PK parameters were estimated for plasma TFV concentrations, serum LNG concentrations, CV and rectal fluid concentrations of TFV and CV tissue concentrations of TFV and TFV-DP. For each sample type and analyte, the following PK parameters were calculated using mean (composite) concentrations from each available nominal time point from pre-insertion (0 hours) at Visit 4 through 72 hours post-insertion (Visit 5):

 AUC_0-24_: Area under the mean concentration-time curve from time zero (pre-insertion) to 24 hours post-insertion using nominal time points and linear trapezoidal summation

 AUC_0-48_: Area under the mean concentration-time curve from time zero (pre-insertion) to 48 hours post-insertion using nominal time points and linear trapezoidal summation

 AUC_0-72_: Area under the mean concentration-time curve from time zero (pre-insertion) to 72 hours post-insertion using nominal time points and linear trapezoidal summation

 C_max_: Maximum value of the mean concentration time curve

 T_max_: Nominal time at which the maximum value of the mean concentration time curve occurred

For each sample type and analyte, the following PK parameter was calculated using mean (composite) concentrations from the post-IVR removal time point at Visit 32 of 48 hours, 72 hours, and 5 days:

 T _1/2_: Apparent terminal half-life (hours), determined as ln2/λz, where λz is the apparent terminal elimination rate constant (1/hours), determined by linear regression of mean log concentrations at the nominal terminal phase time points.

**Levonorgestrel pharmacokinetics and sex hormone binding globulin (SHBG)**

Serum (approximately 150 µL) was mixed with 100 μl ultrapure water (Milli-Q, EMD Millipore, Billerica, MA) containing 1.8 ng/ml LNG-d6 isotopic standard (Toronto Research Chemicals, North York, ON, Canada) and added to a 400 µl Strate DE solid-phase extraction (SPE) plate (Phenomenex, Torrance, CA). LNG was eluted with 3 x 600 µl dichloromethane (Sigma, St. Louis, MO), dried with nitrogen gas (Airgas, Radnor, PA), and reconstituted in 50 μl of 25% (v:v) methanol:ultrapure water. For calibration curves, charcoal-stripped human serum (Biochemed Services, Winchester, VA) was spiked with unlabeled LNG standard (Sigma) in methanol and diluted serially to final concentrations between 0.020 and 20 ng/ml in a 12-point curve. The spiked standard was then extracted as described above. After the reconstitution step, samples were subjected to LC-MS/MS analysis. Using a Shimadzu SIL-30CAMP autosampler, 25 μl of each sample were injected onto a Raptor 2.7 µm Biphenyl 50 mm X 2.1 mm column (Restek, Bellefonte, PA). Mobile phase consisted of 0.15 mM ammonium fluoride (Sigma) in water (A), and methanol with no additive (B) with a flow rate of 0.4 ml/min. The LC time gradient was created using a Shimadzu Nexera LC-30AD system as follows: 0.00 – 0.50 min, hold 76% B; 0.50 – 3.00 min, 76-96% B; 3.00 – 3.10 min, 96-100% B; 3.10 – 4.00 min, hold 100% B; 4.00 – 4.10 min, return to 76% B and hold for re-equilibration until 5.80 min. LNG was detected in positive ion mode with multiple reaction monitoring (MRM) using a Shimadzu LCMS-8050 tandem triple-quadrupole MS with heated electrospray ionization. The MRM transitions used were: LNG, 313.30🡪245.10 (quant), 313.30🡪227.00 (qual), 313.30🡪187.10 (qual), m/z; LNG-d6, 319.30🡪251.25 (quant), 319.30🡪114.10 (qual), m/z. Retention time for LNG was 2.498 min; retention time for LNG-d6 was 2.462 min. Data processing and analysis was performed using LabSolutions Software, V5.72 (Shimadzu). Intra-assay CV for LNG was <6.1% and inter-assay CV for LNG was 11.4% (n=6 assays).

SHBG in serum was analyzed by immunoassay using a Roche Cobas e411 automated clinical platform (Roche Diagnostics, Indianapolis, IN). The assay range for SHBG is 0.033-19 µg/ml. Intra-day CV was <3.8% and inter-day CV was 9.0% (n=10 days).

For serum LNG and SHBG, the following additional PK parameter was calculated from the individual participant concentration versus time data using non-compartmental methods and actual elapsed time from dosing:

 AUC_0-8_: Area under the concentration-time curve from time zero (pre-insertion) to 8 hours post-insertion, calculated by linear trapezoidal summation

**p24 antigen production by tissue biopsies infected ex vivo with HIV-1_BaL_**

We collected the tissue culture supernatant (approximately 300 µL) every three to four days and replenished cultures with media containing IL-2. We stored the supernatants at -80˚C and evaluated them at the end of the culture for HIV-1 p24 antigen expression by ELISA (Perkin Elmer) in pg/mL. Area under the curve (AUC), cumulative (CUM) p24 antigen production, SOFT, and p24 at Day 21 (p24_D21) were calculated as reported [9].

**Levonorgestrel pharmacodynamic surrogate assessment**

To determine the CM Insler score, at least 2 examiners assessed the CM on a scale of 0 – 3 for each factor (spinnbarkeit, volume, viscosity, cellularity and ferning) with a score of 10 or more indicating normal, ovulatory, mid cycle mucus receptive to sperm penetration [10]. An aliquot of CM was placed on a slide and approximately 50 µL of donor sperm and the CM sperm interaction was assessed per standard guidelines [11]. Donor sperm were obtained under an EVMS IRB approved protocol (IRB #13-02-FB-0031).

**REFERENCES FOR SUPPLEMENTAL METHODS**

1. Bushman LR, Kiser JJ, Rower JE, Klein B, Zheng JH, Ray ML, et al. Determination of nucleoside analog mono-, di-, and tri-phosphates in cellular matrix by solid phase extraction and ultra-sensitive LC-MS/MS detection. J Pharm Biomed Anal. 2011;56(2):390-401. Epub 2011/07/01. doi: 10.1016/j.jpba.2011.05.039. PubMed PMID: 21715120; PubMed Central PMCID: PMCPMC3153375.

2. Shieh E, Marzinke MA, Fuchs EJ, Hamlin A, Bakshi R, Aung W, et al. Transgender women on oral HIV pre-exposure prophylaxis have significantly lower tenofovir and emtricitabine concentrations when also taking oestrogen when compared to cisgender men. J Int AIDS Soc. 2019;22(11):e25405. Epub 2019/11/07. doi: 10.1002/jia2.25405. PubMed PMID: 31692269; PubMed Central PMCID: PMCPMC6832671.

3. FDA. Guidance for Industry Bioanalytical Method Validation Guidance for Industry Bioanalytical Method Validation. . 2018;<http://www.fda.gov/Drugs/GuidanceComplianceRegulatoryInformation/Guidances/default.htm>.

4. Wei X, Decker JM, Liu H, Zhang Z, Arani RB, Kilby JM, et al. Emergence of resistant human immunodeficiency virus type 1 in patients receiving fusion inhibitor (T-20) monotherapy. Antimicrob Agents Chemother. 2002;46(6):1896-905. Epub 2002/05/23. doi: 10.1128/aac.46.6.1896-1905.2002. PubMed PMID: 12019106; PubMed Central PMCID: PMCPMC127242.

5. Corporation P. Luminescent Cell Viability Assay Technical Bulletin, TB2882015.

6. Corporation P. Bright-Glo Luciferase Assay System Technical Manual, TM0522015.

7. Keller MJ, Madan RP, Torres NM, Fazzari MJ, Cho S, Kalyoussef S, et al. A randomized trial to assess anti-HIV activity in female genital tract secretions and soluble mucosal immunity following application of 1% tenofovir gel. PLoS One. 2011;6(1):e16475. Epub 2011/02/02. doi: 10.1371/journal.pone.0016475. PubMed PMID: 21283552; PubMed Central PMCID: PMC3026837.

8. Thurman AR, Schwartz JL, Brache V, Clark MR, McCormick T, Chandra N, et al. Randomized, placebo controlled phase I trial of safety, pharmacokinetics, pharmacodynamics and acceptability of tenofovir and tenofovir plus levonorgestrel vaginal rings in women. PLoS One. 2018;13(6):e0199778. Epub 2018/06/29. doi: 10.1371/journal.pone.0199778. PubMed PMID: 29953547; PubMed Central PMCID: PMCPMC6023238.

9. Richardson-Harman N, Lackman-Smith C, Fletcher PS, Anton PA, Bremer JW, Dezzutti CS, et al. Multisite comparison of anti-human immunodeficiency virus microbicide activity in explant assays using a novel endpoint analysis. J Clin Microbiol. 2009;47(11):3530-9. doi: 10.1128/JCM.00673-09. PubMed PMID: 19726602; PubMed Central PMCID: PMC2772583.

10. Insler V, Melmed H, Eichenbrenner I, Serr DM, Lunenfeld B. The Cervical Score: A Simple Semiquantitative Method for Monitoring of the Menstrual Cycle. International Journal of Gynaecology and Obstetrics. 1972;10(6):223-7.

11. Organization WH. The World Health Organization Laboratory Manual for the Examination of Human Semen and Sperm-Cervical Mucus Interaction. Fourth ed. Cambridge, United Kingdom: Cambridge University Press; 1999.

12. Jacot TA, Clark MR, Adedipe OE, Godbout S, Peele AG, Ju S, et al. Development and clinical assessment of new objective adherence markers for four microbicide delivery systems used in HIV prevention studies. Clin Transl Med. 2018;7(1):37. Epub 2018/11/08. doi: 10.1186/s40169-018-0213-6. PubMed PMID: 30402770; PubMed Central PMCID: PMCPMC6219998.
