# Supplementary material for: Randomized, placebo controlled phase I trial of the safety, pharmacokinetics, pharmacodynamics and acceptability of a 90 day tenofovir plus levonorgestrel vaginal ring used continuously or cyclically in women: The CONRAD 138 study
Source: PLoS One. 2022 Oct 10;17(10):e0275794. doi: 10.1371/journal.pone.0275794 (PMC9550080; doi:10.1371/journal.pone.0275794)
Supplement: S1 Fig — (DOC) [file pone.0275794.s007.doc]

**
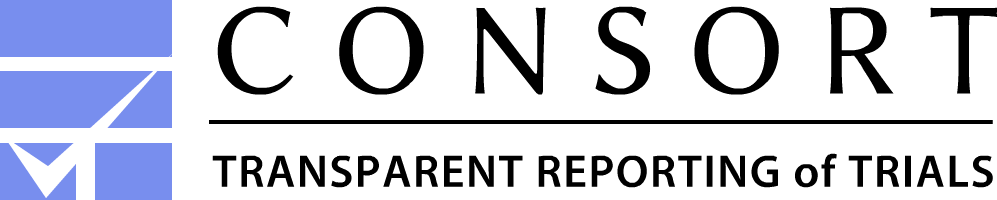
**

**CONSORT 2010 Flow Diagram**

**Allocation**

**Analysis**

**Follow-Up**

**Enrollment**

Assessed for eligibility (n= 68)

Excluded (n= 21)

♦  Not meeting inclusion criteria (n= 21)

♦  Declined to participate (n= 0)

♦  Other reasons (n= 0)

Analysed (n=36)
♦ Excluded from analysis (give reasons) (n=1) due to protocol violations

Lost to follow-up (give reasons) (n= 0)

Discontinued intervention (give reasons) (n=7) for personal subject reasons (n=4), other (n=2), physician decision (n=1)

Allocated to TFVLNG (n= 37)

♦ Received allocated intervention (n= 37)

♦ Did not receive allocated intervention (give reasons) (n= 0)

Lost to follow-up (give reasons) (n= 0)

Discontinued intervention (give reasons) (n=0 )

Allocated to Placebo (n= 10)

♦ Received allocated intervention (n=10)

♦ Did not receive allocated intervention (give reasons) (n=0 )

Analysed (n=10)
♦ Excluded from analysis (give reasons) (n= 0)

Randomized (n=47)
