# Supplementary material for: Randomized, placebo controlled phase I trial of the safety, pharmacokinetics, pharmacodynamics and acceptability of a 90 day tenofovir plus levonorgestrel vaginal ring used continuously or cyclically in women: The CONRAD 138 study
Source: PLoS One. 2022 Oct 10;17(10):e0275794. doi: 10.1371/journal.pone.0275794 (PMC9550080; doi:10.1371/journal.pone.0275794)
Supplement: S1 Protocol — (DOCX) [file pone.0275794.s008.docx]

Tenofovir/Levonorgestrel Intravaginal Ring

**Phase I, 90-Day Safety, Pharmacokinetic, and Pharmacodynamic Study of Intravaginal Rings Releasing Tenofovir and Levonorgestrel**

***Evaluating New Ring Choices (ENRICH)***

**IND #118,510**

**Protocol A15-138**

**22 May 2018**

**VERSION 4.0**

Prepared By:

CONRAD

1911 North Fort Myer Drive

Suite 900

Arlington VA 22209

Phone: 703-524-4744

Investigator’s Agreement

**Phase I, 90-Day Safety, Pharmacokinetic, and Pharmacodynamic Study of Intravaginal Rings Releasing Tenofovir and Levonorgestrel**

***Evaluating New Ring Choices (ENRICH)***

**Protocol A15-138**

**22 May 2018**

**Version 4.0**

*This document may contain confidential information. It is understood that persons to whom this information is disclosed will not disclose it further without permission from CONRAD, unless such information is published or otherwise becomes public knowledge.*

I have received and read the Investigator’s Brochure for the Tenofovir/Levonorgestrel Intravaginal Ring and have read Protocol A15-138 and agree to conduct the study as outlined. I will comply with all requirements regarding the obligations of clinical investigators as fully outlined in 21 Code of Federal Regulations (CFR) Parts 50, 56, and 312.60 and in the Statement of Investigator (1572), which I have also signed. I will ensure that all associates, colleagues, and employees assisting in the conduct of this study are informed about the obligations incurred by their contribution to the study. I agree to maintain the confidentiality of all information received or developed in connection with this protocol.

Printed Name of Investigator

Signature of Investigator

Date

Procedures in Case of Emergency

Table 1: Emergency Contact Information

| Role in Study | Name | Email Address and Telephone number |
| --- | --- | --- |
| Clinical Study Leader  Medical Director / Medical Monitor / SAE Reporting | Jill Schwartz, M.D. | jschwartz@conrad.org  (703) 524-4744 |
| Project Manager | Susan Ju | sju@conrad.org  (703) 524-4744 |

Any changes to these personnel will be documented separately.

1. Synopsis

| **Name of Sponsor:**  CONRAD  1911 North Fort Myer Drive  Suite 900  Arlington VA 22209 | |
| --- | --- |
| **Name of Investigational Product:**  Tenofovir/Levonorgestrel Intravaginal Ring | |
| **Name of Active Ingredients:**  Tenofovir (TFV), Levonorgestrel (LNG) | |
| **Title of Study:**  Phase I, 90-Day Safety, Pharmacokinetic, and Pharmacodynamic Study of Intravaginal Rings Releasing Tenofovir and Levonorgestrel | |
| **Principal Investigator and Study center(s):**   - Andrea Thurman, M.D., Clinical Research Center, Eastern Virginia Medical School, Norfolk, VA, USA - Vivian Brache, Lic., PROFAMILIA, Santo Domingo, Dominican Republic | |
| **Studied period (years):**  Estimated date first participant enrolled: Q3 2017  Estimated date last participant completed: Q4 2018 | **Phase of development:**  Phase I Expanded Safety |
| **Objectives**  ***Primary Objective: Safety***   - Evaluate genital and systemic safety of the TFV/LNG IVR during and after 90 days of continuous use or 90 days of interrupted use (3x28 days)   ***Secondary Objectives:***  **Pharmacokinetics (PK) and Pharmacodynamics (PD)**   - Evaluate PK of TFV - Evaluate PK of LNG - Evaluate PD surrogate markers of contraceptive efficacy of LNG - Evaluate PD surrogate markers of anti-HIV-1 and anti-HSV-2 efficacy of TFV in CV fluid   **Bleeding Patterns**   - Compare bleeding patterns by regimen and by product group   **Forgiveness**   - Evaluate forgiveness of TFV and LNG   **Acceptability**   - Examine user preferences for IVR attributes and user attitudes, beliefs, and experiences towards IVR use by regimen and by product use   **Adherence**   - Assess use of IVR during study period   ***Exploratory Objectives:***  **Pharmacodynamics (PD)**   - Evaluate PD surrogate markers of anti-HIV-1 and anti-HSV-2 efficacy of TFV in rectal fluid - Evaluate PD surrogate markers of anti-HSV-2 efficacy of TFV in CV tissue, as possible - Assess qualitative measure of TFV in a vaginal swab   **Adherence**   - Assess product use through returned IVRs by spectroscopic or other analytical methods - Compare self-report of IVR removal scale to objective biomarkers of IVR use - Assess baseline user characteristics as predictors of adherence   **Endpoints**  ***Primary Endpoints: Safety***   - Treatment-emergent adverse events (TEAEs) - Changes in serum chemistries, lipids, and complete blood count (CBC) - Development of cervicovaginal (CV) ulcerations, abrasions, edema, and other findings as assessed by naked eye visualization of the CV epithelium - Changes in soluble markers of innate mucosal immunity and inflammatory response in *CV fluid* [e.g., IL-1α, IL-6, IL-10, TNFα, RANTES, MIP-1α, IP-10, GM-CSF, IL-8, IL-1RA, SLPI, and BD2] - Changes in HIV-1 target immune cell phenotype (e.g., CD45, CD68, CD4, and CD1a) and HIV-1 activation/proliferation markers (e.g., CD38, CCR5, HLA-DR, Ki67) in *CV tissue* - Changes in hydrogen peroxide-secreting Lactobacilli concentration and other endogenous vaginal bacteria by quantitative PCR - Microbial growth on returned IVRs   ***Secondary Endpoints:***  **Pharmacokinetics of TFV and LNG**   - TFV concentrations in *plasma*, *CV fluid*, *rectal fluid*, and *CV tissue* - TFV-DP concentrations in *CV tissue* - LNG concentrations in *serum* - Residual drug (TFV and LNG) in returned IVRs   **Pharmacodynamics of LNG**  Surrogates of contraceptive efficacy:   - Cervical mucus assessment   - Cervical mucus quality (score of >10)   - Sperm migration on the Simplified Slide test - Ovulation by serum progesterone (P4) - Effect on follicular development by serum estradiol concentration   **Pharmacodynamics of TFV**   - Anti-HIV-1 activity in *CV* *fluid* - Anti-HSV-2 activity in *CV* *fluid* - Comparison of HIV-1 ex vivo infection in *CV tissue* (EVMS only) at baseline and after 90 days of IVR use   **Bleeding Patterns**   - Participant self-report of bleeding   **Forgiveness**   - Decay of LNG during 3-day periods of non-use in interrupted regimen, and after 90 days of IVR use - Decay of TFV during 3-day periods of non-use in interrupted regimen, and after 90 days of IVR use   **Acceptability**   - Responses to key questions on acceptability and psychosocial questionnaire(s) (all participants), and feedback during in-depth interviews (subset of participants)   **Adherence**   - Number and percentage of participants with Discontinuations/Expulsions/Removals by self-report   ***Exploratory Endpoints:***  **Pharmacodynamics of TFV**   - Anti-HIV-1 activity in *rectal fluid* - Anti-HSV-2 activity in *rectal fluid* - Comparison of HSV-2 ex vivo infection in *CV tissue* (EVMS-only) at baseline and after 90 days of IVR use, as possible - Qualitative measure of TFV in a vaginal swab   **Adherence**   - Presence or absence of spectroscopic pattern signatures or other analytical measures of drug or placebo products (EVMS only) - Characterization of returned IVRs (active and placebo) via objective IVR biomarkers (e.g., residual glycerin content and bioassay) and residual drug (TFV and LNG), as feasible - Correlation of IVR removal scale factors and objective biomarkers of IVR use - Correlation of baseline user characteristics and objective biomarkers of IVR use | |
| **Study Design:**  This will be a Phase I, randomized, placebo-controlled, parallel study. A total of 60 eligible participants will be randomized in a 4:4:1:1 ratio to study arm (see Table below), where Continuous arms will use the assigned IVR for 90 days continuously, and where Interrupted arms will use the assigned IVR for 3 periods of 28 days with 3 IVR-free days in between.  Randomization Assignments   \| Arm \| N \| Dose \| Regimen \| \| --- \| --- \| --- \| --- \| \| 1 \| 24 \| TFV/LNG IVR (8-10mg/20μg) \| 90 Days (Continuous) \| \| 2 \| 24 \| TFV/LNG IVR (8-10mg/20μg) \| 3x28 Days (Interrupted) \| \| 3 \| 6 \| Placebo \| 90 Days (Continuous) \| \| 4 \| 6 \| Placebo \| 3x28 Days (Interrupted) \|   **Visit 1 (Screening):** All women will undergo a screening visit to determine the presence of exclusionary criteria to confirm that they are eligible to continue in the study.  **Visit 2 (Confirmation of ovulation):** Participants will undergo testing on cycle day 21 to confirm ovulation (P4 level of ≥3.0 ng/ml), and, if confirmed, will proceed to Visit 3.  **Visit 3 (Baseline and Randomization)**: On cycle day 24, participants will be asked to respond to psychosocial acceptability questions. Cervicovaginal (CV) fluid will be collected for semen testing and CV biopsies will be collected for target immune cells and HIV-1 activation/proliferation markers, and HIV-1 and HSV-2 ex-vivo infectivity (EVMS only). Participants will be randomized to study arm and will also receive a random time point assignment at this visit for post-IVR insertion sample collection at Visit 5.  **Visit 4 (IVR Insertion):** On cycle day 6, baseline samples for blood (TFV/LNG/SHBG/estradiol/P4), CV and rectal fluid for anti-HIV-1 and anti-HSV-2 activity, CV fluid for semen testing, soluble markers, and microflora (qPCR) will also be collected. Initiation of IVR will occur. After IVR insertion, blood samples for LNG/SHBG PK will be collected at four time points (1, 2, 4, and 8 hours), blood for TFV PK will be collected at 8 hours, and CV fluid for TFV PK will be collected at 2 and 8 hours.  **Visit 5 (Post insertion sample collection):** Based on randomized assignment to 24, 48, or 72 hours after IVR insertion, blood for TFV/LNG/SHBG, CV fluid and rectal fluid for TFV, and CV tissue for TFV/TFV-DP concentrations will be collected.  **Visits 6, 7, 9, 10, 12, 15, 16, 18, 19, 21, 24, 25, 27, 28, and 30 (P4/Estradiol):** Blood for P4 and estradiol concentrations will be collected approximately twice a week throughout IVR use, and cervical mucus will be collected once during each month of IVR use, according to estradiol/P4 concentrations. At Visit 27 (if not done at Visit 26), participants will receive a random time point assignment for post IVR removal sample collection at Visit 32.  Guidelines for Cervical Mucus Collection   \| Estradiol concentration \| Cervical Mucus Collection \| \| --- \| --- \| \| Is between 75 - 150 pg/ml \| The participant will be asked to come in at their next scheduled visit for cervical mucus collection. Participants should be instructed to abstain from vaginal/anal intercourse/activity for 48 hours prior to cervical mucus collection, as possible. \| \| Is >150 pg/ml \| The participant will be asked to come in ideally within 24 hours of results being known for cervical mucus collection. Collection can take place the same day that results are known, per investigator discretion. \| \| Does not reach 75 pg/ml before the end of each month (i.e., days 28, 59, and 90) of IVR use \| Cervical mucus will be collected at the end of each month of IVR use (ideally days 28, 59, and 90 of IVR use). \| \| Note that P4 must be <3ng/ml to collect cervical mucus. Once cervical mucus has been collected for that month, it should not be collected again until the next month unless there are extenuating circumstances (e.g., unviable sample), though estradiol and P4 samples will continue to be collected twice weekly. \| \|   Within the first month of IVR use, preferably at either Visit 6 or Visit 7, an in-depth interview will be conducted for a subset of participants.  **Visits 8, 17, and 26 (Months 1, 2, and 3):** Blood for TFV/LNG/SHBG/estradiol/P4, and CV fluid for TFV PK will be collected. Cervical mucus may also be collected at this visit (see Table above). At Visits 8 and 17, CV fluid will be collected for semen testing. At Visit 26 (if not done at Visit 27), participants will receive a random time point assignment for post IVR removal sample collection at Visit 32.  **Visits 11, 20, and 29 (Months 1, 2, and 3):** Blood for TFV/LNG/SHBG/estradiol/P4, and CV and rectal fluid for TFV PK will be collected. Cervical mucus may also be collected at this visit (see Table above). At Visits 11 and 29, CV fluid will be collected for anti-HIV-1 and anti-HSV-2 activity and semen testing. At Visit 29, rectal fluid will also be collected for anti-HIV-1 and anti-HSV-2 activity, as well as CV fluid for soluble markers.  **Visits 13, 22, and 31 (Months 1, 2, and 3):** Blood for TFV/LNG/SHBG/estradiol/P4 will be collected. Cervical mucus may also be collected at this visit (see Table above). Vaginal fluid will be collected for qualitative measure of TFV and for microflora. At Visits 13 and 31, participants will also be asked to provide feedback about their experience with the IVR via a questionnaire. At Visits 13 and 22, participants in Arms 2 and 4 will remove the IVR in clinic under observation, and their IVR will be stored.  At Visit 31, blood for CBC, serum chemistries, and fasting lipids will be collected; the IVR will be removed; CV tissue for TFV/TFV-DP concentrations, HIV-1 and HSV-2 ex-vivo infectivity (EVMS only), and immune cell and activation markers will be collected; an in-depth interview may be conducted for a subset of participants anytime starting at Visit 31 through one week after Visit 32.  **Visits 14 and 23:** Blood for TFV/LNG/SHBG/estradiol/P4 will be collected. Cervical mucus may also be collected at this visit (see Table above). Participants in Arms 2 and 4 will re-insert their [stored] IVR. For all participants, CV fluid for TFV PK and semen testing will be collected.  **Visit 32 (Post removal sample collection):** Based on randomized assignment to 48 hours, 72 hours, or 5 days after IVR removal, blood for TFV/LNG/SHBG and HIV-1, CV and rectal fluid for TFV PK; and CV tissue for TFV/TFV-DP concentrations will be collected.  **Follow Up Contact:** A final site contact will be scheduled for approximately 1 - 2 weeks after the last visit to ask the participant about adverse events (AEs) and concomitant medications (CMs). The participant will be exited unless there are symptoms that require follow up.  Participants will be given a guidebook in which to record AEs and CMs. Menses, bleeding/spotting, and vaginal/anal activity/intercourse will also be recorded in the guidebook.  Participants will abstain from the use of vaginal products (other than the study product and condoms) including tampons (except for menses), spermicides, lubricants, and douches for the whole study. Participants will abstain from vaginal and anal intercourse/activity starting 48 hours prior to cervical mucus collections, as possible, and 48 hours before Visits 4 and 29; and for 5 days after CV tissue collection. For sex acts during the study, non-spermicidal condoms must be used.  An interim analysis may be conducted to evaluate drug-release parameters to help inform product development. | |
| **Number of participants (planned):** Approximately 120 women may be consented and undergo assessment procedures in order to have at least 60 women complete the study. Women will be randomized in a 4:4:1:1 ratio (see Table “Randomization Assignments” above). A subset of approximately 20 women will be selected for an in-depth interview to take place during the first month of IVR use and again after 90 days of use. | |
| **Diagnosis and main criteria for inclusion:**  This study will recruit healthy, non-pregnant, ovulatory, HIV-uninfected women aged 18 to 50 with a body mass index (BMI) less than 30 kg/m^2^, regular menstrual cycles (approximately 26-35 days) by participant report, and willing to use non-spermicidal condoms for sex and follow other study restrictions. Women will be protected from pregnancy by abstinence from penile-vaginal intercourse, same-sex relationship, consistent use of non-spermicidal condoms, or sterilization of either partner. | |
| **Investigational product, dosage, and mode of administration:**  IVR releasing 8-10 mg of TFV and 20 μg of LNG per day  Mode of administration: intravaginal | |
| **Reference therapy, dosage and mode of administration:**  Placebo IVR  Mode of administration: intravaginal | |
| **Duration of treatment:**  90 consecutive days or 3x28 days of IVR use, depending on randomization to regimen | |
| **Criteria for evaluation:**  ***Safety:*** It is expected that there will be no clinically significant concerns in any of the product arms based on previous use. | |
| **Statistical methods:**  *Sample Size:* Sample size for this Phase I study is primarily based upon the size of similar studies and feasibility although statistical considerations are considered.  *Analysis Populations:*  The Randomized Population (RP) includes all randomized participants.  The Treated Population (TP) is a subset of RP and will consist of all randomized participants with any IVR use.  The Evaluable Population (EP) is a subset of TP and will consist of all randomized participants with any IVR use and contributing at least some follow-up safety or PK/PD data. The Evaluable Population will be the primary analysis population for most objectives.  *Evaluation of Study Objectives:* Study objectives will be evaluated by clinical review of descriptive summaries and graphical displays.  Analysis of safety will include summaries by treatment group of the incidence of treatment-emergent adverse events and incidence of other findings from pelvic examinations (e.g., ulcerations, abrasions, edema, and bleeding). Changes from baseline in clinical laboratory endpoints, soluble markers of mucosal immunity, HIV-1 target immune cell and activation markers, and microflora bacteria concentrations will be summarized by treatment group and time point. Shift tables will be produced where relevant.  PK analysis will include descriptive statistics by time point of TFV concentrations for all plasma, tissue, and fluid sample types, in addition to LNG and SHBG concentrations from plasma samples and TFV-DP concentrations from CV tissue samples. PK parameters (e.g., C_max_, T_max_, AUC) of TFV, TFV-DP, and LNG will be estimated by non-compartmental analysis of the concentration time curve or mean (composite) concentration time curve as appropriate. Comparisons of TFV concentrations and select PK parameters of TFV concentration will be made between active [continuous TFV/LNG IVR (8-10mg/20μg) vs. interrupted TFV/LNG IVR (8-10mg/20μg)] treatment groups.  PD endpoints will be summarized using descriptive statistics by time point as well as changes from baseline. Estimates and 95% confidence intervals will be provided. Comparison between treatment groups may be performed for selected endpoints using an analysis of covariance (ANCOVA) model though it is acknowledged that the trial may not be powered to test hypotheses regarding treatment group differences. Analysis will include summaries of P4 and estradiol concentrations over time, HIV-1 and HSV-2 infectivity endpoints from CV tissue [p24, cumulative p24 (CUM; the summation of p24 values measured by Elisa from Day 0 to Day 21), AUC (area under the virus growth curve), and p24 and the soft endpoint (SOFT)] before and after treatment, anti-HIV-1/anti-HSV-2 activity in CV and rectal fluid, and qualitative measurement of TFV in CV fluid.  Other secondary and exploratory endpoints will be summarized by treatment group using descriptive statistics, as appropriate. Endpoints of quantitative adherence (acceptability questionnaire) will be largely descriptive and will be collected via a GCP-compliant electronic survey system. IDIs will be audio-recorded, transcribed, and translated as necessary, for qualitative analysis.  We may explore correlations between IVR scales collected at Visits 3, 13, and 31 with biomarker adherence measures.  *Descriptive Statistics:* Categorical variables will be summarized by frequencies and percentages. Continuous variables will be summarized by means, standard deviations, medians, quartiles, minima and maxima. Summaries of PK concentrations will also include the geometric mean and geometric coefficient of variation.  *Imputation:* In terms of summarizing concentration data, values that are below the lower limit of quantification (LLOQ) will be handled as follows: pre-dose concentrations will be set to zero and post-dose concentrations will be set to one-half the value LLOQ. Otherwise, no imputation is anticipated. If needed, rules for imputing missing values will be created and will be documented in the SAP or statistical report.  *Control of Type I Error:* This Phase I study is descriptive in nature. Any p-values or confidence intervals around estimates of treatment differences will not be adjusted for multiplicity and are descriptive in nature. These statistics will be provided to guide clinical judgment; caution must be used as these statistics will not have a controlled Type I error.  *Definition of Baseline:* Baseline is defined as the non-missing value obtained closest to but prior to the first product use.  *Interim Analysis:* An interim analysis may be conducted to evaluate drug-release parameters to help inform the direction of further clinical development (i.e., additional studies). The analysis details of any interim analysis will be described in the SAP. | |

Figure 1: Study Schematic


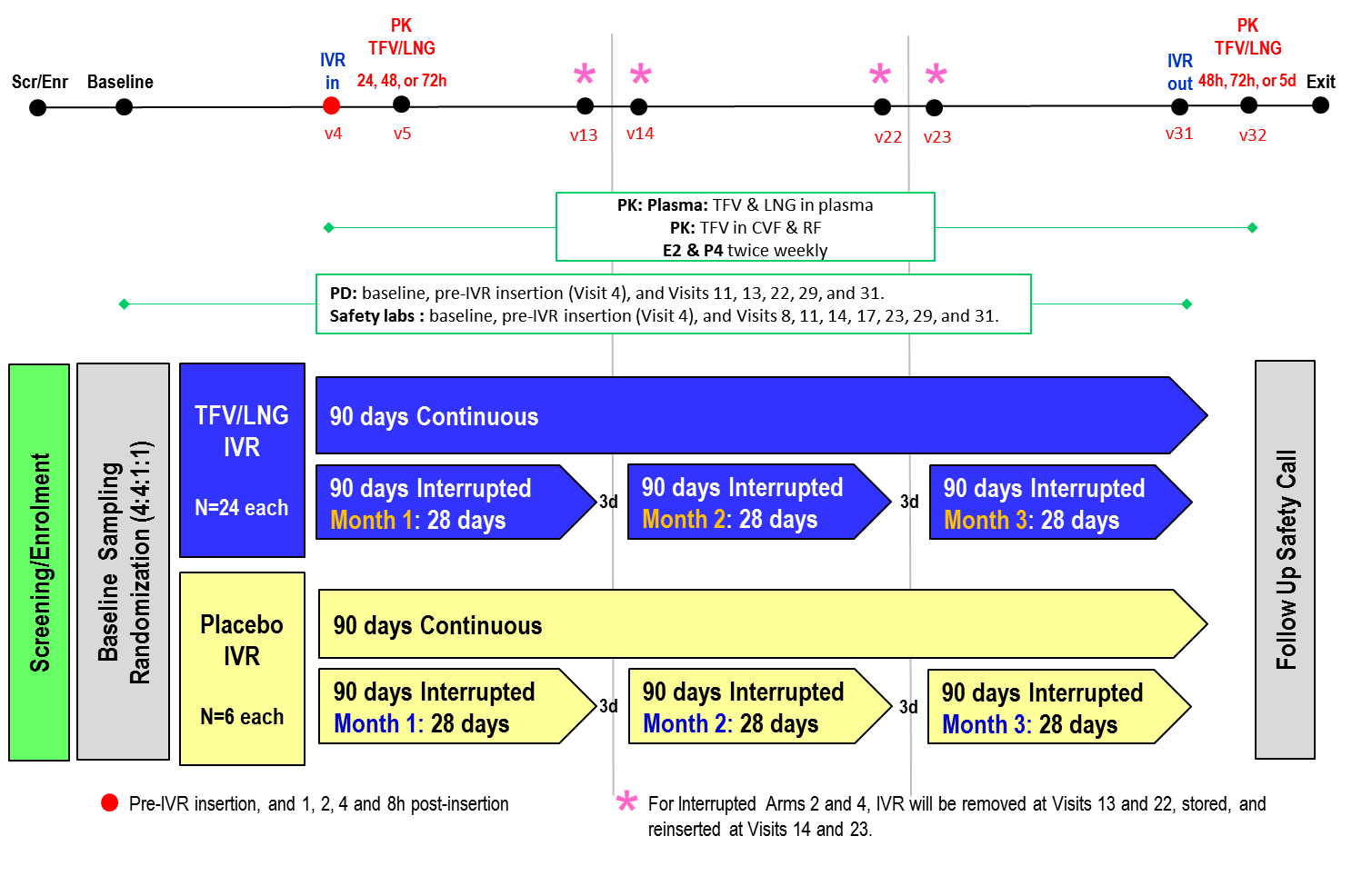


1. Table of Contents, List of Tables, and List of Figures

Table of Contents

[1. Synopsis 4](#_Toc513805352)

[2. Table of Contents, List of Tables, and List of Figures 14](#_Toc513805353)

[3. List of Abbreviations and Definitions of Terms 21](#_Toc513805354)

[4. Introduction 24](#_Toc513805356)

[5. Trial purpose, Objectives and Endpoints 26](#_Toc513805357)

[5.1. Purpose 26](#_Toc513805358)

[5.2. Objectives 26](#_Toc513805359)

[5.2.1. Primary Objective: Safety 26](#_Toc513805360)

[5.2.2. Secondary Objectives: Pharmacokinetics (PK) and Pharmacodynamics (PD) 26](#_Toc513805361)

[5.2.3. Secondary Objective: Bleeding Patterns 26](#_Toc513805362)

[5.2.4. Secondary Objective: Forgiveness 26](#_Toc513805363)

[5.2.5. Secondary Objective: Acceptability 26](#_Toc513805364)

[5.2.6. Secondary Objective: Adherence 26](#_Toc513805365)

[5.2.7. Exploratory Objectives: Pharmacodynamics (PD) 26](#_Toc513805366)

[5.2.8. Exploratory Objectives: Adherence 26](#_Toc513805367)

[5.3. Endpoints 27](#_Toc513805368)

[5.3.1. Primary Endpoints: Safety 27](#_Toc513805369)

[5.3.2. Secondary Endpoints: Pharmacokinetics of TFV and LNG 27](#_Toc513805370)

[5.3.3. Secondary Endpoints: Pharmacodynamics of LNG 27](#_Toc513805371)

[5.3.4. Secondary Endpoints: Pharmacodynamics of TFV 27](#_Toc513805372)

[5.3.5. Secondary Endpoints: Bleeding Patterns 28](#_Toc513805373)

[5.3.6. Secondary Endpoints: Forgiveness 28](#_Toc513805374)

[5.3.7. Secondary Endpoints: Acceptability 28](#_Toc513805375)

[5.3.8. Secondary Endpoints: Adherence 28](#_Toc513805376)

[5.3.9. Exploratory Endpoints: Pharmacodynamics of TFV 28](#_Toc513805377)

[5.3.10. Exploratory Endpoints: Adherence 28](#_Toc513805378)

[6. Investigational Plan 29](#_Toc513805379)

[6.1. Overall Study Design 29](#_Toc513805380)

[6.2. Anticipated Length of Study 34](#_Toc513805381)

[6.3. Number of Subjects 34](#_Toc513805382)

[6.4. Treatment Assignment 34](#_Toc513805383)

[6.5. Criteria for Study Termination 34](#_Toc513805384)

[7. Selection and Withdrawal of participants 35](#_Toc513805385)

[7.1. Subject Inclusion Criteria 35](#_Toc513805386)

[7.2. Subject Exclusion Criteria 36](#_Toc513805387)

[7.3. Participant Withdrawal 37](#_Toc513805388)

[7.3.1. Withdrawal Criteria 37](#_Toc513805389)

[7.3.2. Target Enrollment 37](#_Toc513805390)

[7.3.3. Follow Up for Discontinued Participants 37](#_Toc513805391)

[8. Study Procedures 38](#_Toc513805392)

[8.1. Visit 1: Screening 38](#_Toc513805393)

[8.2. Visit 2: Confirmation of Ovulation 39](#_Toc513805394)

[8.3. Visit 3: Baseline and Randomization 40](#_Toc513805395)

[8.4. Visit 4: IVR Insertion 41](#_Toc513805396)

[8.5. Visit 5: Post-Insertion Sample Collection (24, 48, or 72 hours) 42](#_Toc513805397)

[8.6. Visits 6, 7, 9, 10, 12, 15, 16, 18, 19, 21, 24, 25, 27, 28, 30 (P4/Estradiol) 43](#_Toc513805398)

[8.7. Guidelines for Cervical Mucus Collection 44](#_Toc513805399)

[8.8. Visits 8, 17, and 26 (Months 1, 2, and 3) 44](#_Toc513805400)

[8.9. Visits 11, 20, and 29 (Months 1, 2, and 3) 45](#_Toc513805401)

[8.10. Visits 13, 22, and 31 (Months 1, 2, and 3) 46](#_Toc513805402)

[8.11. Visits 14 and 23 (Months 1 and 2) 47](#_Toc513805403)

[8.12. Visit 32: Post-Removal Sample Collection (48 or 72 hours, or 5 days) 48](#_Toc513805404)

[8.13. Follow-Up Call/Contact 49](#_Toc513805405)

[8.14. Unscheduled Visits 49](#_Toc513805406)

[8.15. Early Discontinuations 49](#_Toc513805407)

[9. Treatment of Participants 50](#_Toc513805408)

[9.1. Description of Study Drug 50](#_Toc513805409)

[9.2. Concomitant Medications 50](#_Toc513805410)

[9.3. Treatment Compliance 51](#_Toc513805411)

[9.4. Randomization and Blinding 51](#_Toc513805413)

[10. Study DRug Materials and Management 52](#_Toc513805414)

[10.1. Description of Study Drug 52](#_Toc513805415)

[10.2. Study Drug Packaging and Labeling 52](#_Toc513805416)

[10.3. Study Drug Storage 53](#_Toc513805417)

[10.4. Study Drug Preparation 53](#_Toc513805418)

[10.5. IVR Insertion and Removal 53](#_Toc513805419)

[10.6. Study Drug Accountability 53](#_Toc513805420)

[10.7. Study Drug Handling and Disposal 53](#_Toc513805421)

[11. Assessment of Endpoints 54](#_Toc513805422)

[11.1. Pharmacokinetics 54](#_Toc513805423)

[11.1.1. Pharmacokinetics: Blood Sample Collection 54](#_Toc513805424)

[11.1.2. Pharmacokinetics: Genital and Rectal Sample Collection 54](#_Toc513805425)

[11.2. Assessment of Pharmacodynamics 55](#_Toc513805426)

[11.2.1. Pharmacodynamics: Blood Sample Collection 55](#_Toc513805427)

[11.2.2. Pharmacodynamics: Genital and Rectal Sample Collection 55](#_Toc513805428)

[12. Assessment of Safety 56](#_Toc513805429)

[12.1. Medical History 56](#_Toc513805430)

[12.2. Physical and Pelvic Examination 56](#_Toc513805431)

[12.3. Laboratory Assessments 58](#_Toc513805432)

[12.3.1. Blood Samples 58](#_Toc513805433)

[12.3.2. Genital Samples 58](#_Toc513805434)

[12.3.3. Pregnancy 58](#_Toc513805435)

[13. Adverse and Serious Adverse Events 59](#_Toc513805436)

[13.1. Definition of Adverse Events (AE) 59](#_Toc513805437)

[13.1.1. Suspected Adverse Reaction 59](#_Toc513805438)

[13.1.2. Adverse Reaction 59](#_Toc513805439)

[13.1.3. Serious Adverse Event (SAE) or Serious Suspected Adverse Reaction 59](#_Toc513805440)

[13.1.4. Unexpected Adverse Event or Unexpected Suspected Adverse Reaction 60](#_Toc513805441)

[13.1.5. Serious and Unexpected Suspected Adverse Reaction 60](#_Toc513805442)

[13.2. Relationship to Study Product 61](#_Toc513805443)

[13.3. Recording and Grading Adverse Events for Severity 61](#_Toc513805444)

[13.4. Reporting Serious Adverse Events 62](#_Toc513805445)

[13.5. Temporary Product Hold/Permanent Discontinuation in Response to Adverse Events 63](#_Toc513805446)

[13.5.1. Grade 1 or 2 63](#_Toc513805447)

[13.5.2. Grade 3 and 4 63](#_Toc513805448)

[14. Statistics 64](#_Toc513805449)

[14.1. Sample Size Justification 64](#_Toc513805450)

[14.2. General Statistical Issues 65](#_Toc513805451)

[14.3. Analysis Populations 65](#_Toc513805452)

[14.4. Statistical Analysis 65](#_Toc513805453)

[14.5. Interim Analysis 66](#_Toc513805454)

[15. management of Intercurrent Events 67](#_Toc513805455)

[15.1. Loss to Follow-up 67](#_Toc513805456)

[15.2. Protocol Adherence 67](#_Toc513805457)

[15.3. Protocol Violations 67](#_Toc513805458)

[15.4. Modification of Protocol 68](#_Toc513805459)

[16. Direct access to source data/documents 69](#_Toc513805460)

[16.1. Study Monitoring 69](#_Toc513805461)

[16.2. Audits and Inspections 69](#_Toc513805462)

[16.3. Institutional Review Board (IRB) 70](#_Toc513805463)

[17. quality control and quality assurance 71](#_Toc513805464)

[18. ethics 72](#_Toc513805465)

[18.1. Ethics Review 72](#_Toc513805466)

[18.2. Ethical Conduct of the Study 72](#_Toc513805467)

[18.3. Written Informed Consent 72](#_Toc513805468)

[18.3.1. Procedure for Obtaining Informed Consent 72](#_Toc513805469)

[18.3.2. Subject Confidentiality 73](#_Toc513805470)

[19. Data handling and recordkeeping 74](#_Toc513805471)

[19.1. Method of Data Capture 74](#_Toc513805472)

[19.2. Inspection of Records 74](#_Toc513805473)

[19.3. Retention of Records 74](#_Toc513805474)

[20. Investigator Responsibilities 75](#_Toc513805475)

[20.1. Prior to Starting Study 75](#_Toc513805476)

[20.1.1. Signing of Investigator’s Agreement and Amendments 75](#_Toc513805477)

[20.1.2. Forms and Records 75](#_Toc513805478)

[20.2. During the Study: Forms and Records 76](#_Toc513805479)

[20.3. During the Study: Progress Reports 76](#_Toc513805480)

[21. Publication Policy 77](#_Toc513805481)

[22. References 78](#_Toc513805482)

List of Tables

[Table 1: Emergency Contact Information 3](#_Toc513805483)

[Table 2: Abbreviations and Specialist Terms 21](#_Toc513805484)

[Table 3: Randomization Assignments 29](#_Toc513805485)

[Table 4: Guidelines for Cervical Mucus Collection 30](#_Toc513805486)

[Table 5: Schedule of Evaluations 32](#_Toc513805487)

[Table 6: Investigational Product 50](#_Toc513805488)

[Table 7: PK: Blood Sample Collection 54](#_Toc513805489)

[Table 8: PK: Genital and Rectal Sample Collection 54](#_Toc513805490)

[Table 9: PD: Blood Sample Collection 55](#_Toc513805491)

[Table 10: PD: Genital and Rectal Sample Collection 55](#_Toc513805492)

[Table 11: Safety Management Plan for Genital Findings 57](#_Toc513805493)

[Table 12: Safety: Genital Assessments 58](#_Toc513805494)

[Table 13: Grading the Severity of Adverse Events 61](#_Toc513805495)

[Table 14: Exact Two-sided 95% Confidence Intervals Based on Observing a Particular Rate of Safety Endpoints for Groups of Size 24 and 48 64](#_Toc513805496)

[Table 15: Difference in the Observed Rates of Safety Events 64](#_Toc513805497)

List of Figures

[Figure 1: Study Schematic 12](#_Toc513805498)

[Figure 2: Study Drug Label 52](#_Toc513805499)

1. List of Abbreviations and Definitions of Terms

Table 2: Abbreviations and Specialist Terms

| AE | Adverse event |
| --- | --- |
| AIDS | Acquired immunodeficiency syndrome |
| ANCOVA | Analysis of covariance |
| API | Active pharmaceutical ingredients |
| BMI | Body mass index |
| BV | Bacterial vaginosis |
| CAPRISA | Centre for the AIDS Programme of Research in South Africa |
| CBC | Complete blood count |
| CFR | Code of Federal Regulations |
| CI | Confidence Interval |
| CLIA | Clinical Laboratory Improvement Act |
| CM | Concomitant Medication |
| CRA | Clinical Research Associate |
| CRF | Case report form |
| CT | *Chlamydia trachomatis* |
| CV | Cervicovaginal |
| CVL | Cervicovaginal lavage |
| DAIDS | Division of AIDS |
| eCRF | Electronic case report form |
| EP | Evaluable population |
| EVMS | Eastern Virginia Medical School |
| FDA | U.S. Food and Drug Administration |
| FWA | Federal-wide assurance |
| GC | *Neisseria gonorrhea* |
| GCP | Good Clinical Practice |
| GMP | Good Manufacturing Practice |
| HBsAG | Hepatitis B surface antigen |
| HIPAA | The Health Insurance Portability and Accountability Act |
| HIV | Human Immunodeficiency Virus |
| HSV | Herpes Simplex Virus |
| ICH | International Council for Harmonization |
| IDI | In-depth interview |
| IEC | Independent Ethics Committee |
| IL | Interleukins |
| IND | Investigational New Drug |
| IRB | Institutional Review Board |
| IUD | Intrauterine device |
| IVR | Intravaginal Ring |
| LDC | Less developed country |
| LLOQ | Lower level of quantification |
| LNG | Levonorgestrel |
| MPA | Multiple Project Assurance |
| MPT | Multipurpose Prevention Technology |
| NDA | New Drug Application |
| NIH | National Institutes of Health |
| NSAIDs | Nonsteroidal Anti-inflammatory Drug |
| OC | Oral contraceptive |
| P4 | Serum progesterone |
| PD | Pharmacodynamic |
| PI | Principal Investigator |
| PK | Pharmacokinetic |
| qPCR | Quantitative Polymerase Chain Reaction |
| RP | Randomized population |
| SAE | Serious adverse event |
| SAP | Statistical analysis plan |
| SHBG | Sex hormone binding globulin |
| SLPI | Secretory leukocyte protease inhibitor |
| STI | Sexually transmitted infection |
| TDF | Tenofovir disoproxil fumarate (Viread^®^) |
| TFV | Tenofovir |
| TP | Treated population |
| TV | *Trichomonas vaginalis* |
| UTI | Urinary tract infection |
| WHO | World Health Organization |

1. Introduction

Almost half of all pregnancies worldwide, estimated to be over 100 million annually, are unintended.1^,^2 Despite the existence of a variety of effective contraceptives, discontinuation and non-use remain high, primarily due to side effects, cost, inconvenient dosing schedules, limited access to prescription products and/or poor acceptance of the method by the male partner, resulting in an unacceptably high rate of unintended or mistimed pregnancies. There is a compelling need for innovative and acceptable contraceptives to fill gaps in the existing method mix available worldwide. Statistics clearly show an unmet need for highly effective contraception, especially in less developed countries (LDCs), where 99% of maternal deaths occur.1^,^3 Not surprisingly, these countries, especially those in sub-Saharan Africa and south Asia, are also at the core of the acquired immunodeficiency syndrome (AIDS) epidemic.4

Over 33 million people worldwide are infected with human immunodeficiency virus type 1 (HIV-1) and 22.4 million live in sub-Saharan Africa.4 Women increasingly bear the burden of the HIV-1 pandemic, with more than 60% of new infections occurring in women in sub-Saharan Africa.4 Most women acquire HIV-1 through heterosexual contact with an infected male partner, often as they are unable to negotiate condom use. Poverty, malnutrition, lack of education, and gender inequality fuel both unplanned pregnancies and HIV-1 transmission. There are a significant number of women, especially in LDCs, needing protection against sexually transmitted infections (STIs), in particular HIV-1. These women also need long-term, highly effective contraceptive methods to provide optimal birth spacing and family size. Highly effective contraceptives (e.g., sterilization, intrauterine devices, hormonal contraception) typically provide little or no protection against STIs, while barrier methods (e.g., male or female condoms) have unacceptably high contraceptive failure rates with typical use.

A major breakthrough in HIV-1 prevention was achieved with the announcement of the Centre for the AIDS Programme of Research in South Africa (CAPRISA) 004 trial results at the 2010 International AIDS Conference.5 The first randomized, double blind, placebo-controlled trial of tenofovir (TFV) 1% vaginal gel found a 39% overall reduction in HIV-1 incidence (Intent-to-Treat analysis) and a 54% reduction among women with high adherence to gel use.5 TFV gel applied before and after vaginal intercourse also reduced herpes simplex virus type 2 (HSV-2) acquisition by 51% among women in the CAPRISA 004 trial.

There is an urgent need to expand upon the successes of recent advances in HIV-1 prevention and past novel contraceptive delivery systems to further integrate the priorities of family planning and reproductive health especially in highly vulnerable populations in LDCs. CONRAD plans to meet this need by developing a “dual-protection” product, containing both levonorgestrel (LNG) and TFV providing both contraceptive and microbicidal activity (against HIV-1 and HSV-2), that is safe, highly effective, acceptable and affordable.

LNG has an established track record of safety and efficacy and is, arguably, the best progestin to be incorporated in a controlled-release device, due to its low molecular weight, physical stability, and steady micro-dose release rate from an IVR.6 The contraceptive efficacy of a 20 μg/day LNG IVR has been clearly demonstrated in two clinical studies.7,8 The first was carried out by the World Health Organization (WHO) in 1005 women and yielded a life table pregnancy rate (intrauterine) with the IVR in situ of 3.6 per 100 women (95% CI 2.2-5.0) and of ectopic pregnancy 0.2% (one woman).7 This pregnancy rate was felt to compare favorably with a combined oral contraceptive (OC) tested by WHO and was less than half the pregnancy rate of a progestin-only OC tested by WHO.9,10 Likewise, the ectopic pregnancy rate was within the range of ectopic pregnancy rates seen with copper intrauterine devices (IUDs) and progestin-only OCs. The second study was carried out in the United Kingdom among 1710 women and yielded a 1-year total pregnancy rate of 5.1% (CI 3.6-6.6%) and a 2-year rate of 6.5% (CI 4.4-8.6%).8 The corresponding method failure rates were 4.0% and 1.2%. This was felt to be comparable to pregnancy rates seen with progestin-only OCs.

TFV is a nucleotide reverse transcriptase inhibitor orally administered as a bioavailable prodrug, tenofovir disoproxil fumarate (TDF; Viread^®^). TFV is an important component of today’s anti-HIV-1 therapeutic drug armamentarium and, with over 3.5 million patient-years of use, has been demonstrated to be safe and effective, with low risk of developing resistance. Safety, PK and acceptability studies of TFV 1% gel have demonstrated its suitability as a microbicide. However, TFV microbicide gel has some potential disadvantages, in terms of the practicality of the dosing regimen. Specifically, participants enrolled in CAPRISA were instructed to insert the first dose of TFV gel vaginally within the 12 hours prior to intercourse, as soon as possible within the 12 hours after intercourse and to use no more than 2 doses in one 24 hour period.5 We believe that an IVR, which would eliminate the need for pre-coital and post-coital administration, would enhance adherence and assure optimal genital tract tissue levels of drug prior to virus exposure. Preclinical animal studies have shown similar TFV tissue concentrations after TFV 1% vaginal gel and TFV IVR applications.

The CONRAD IVR was designed to be similar in appearance to NuvaRing^®^, and is within the range of stiffness observed with marketed rings (e.g., slightly stiffer than NuvaRing, slightly less stiff than Estring^®^). CONRAD 128, a Phase I trial to study the safety, PK, PD, and acceptability of the TFV/LNG IVR for one month in women has completed. Conclusions include: no safety concerns, LNG and TFV PK benchmarks met, TFV effect showing anti-HIV-1 activity, surrogates of contraceptive performance within specifications, and high TFV-DP levels supporting potential pharmacological forgiveness.11 Tissue levels of TFV-DP, the active metabolite of TFV, were similar or exceeded those obtained with the gel after a BAT24 regimen tested in CAPRISA 004 or a daily regimen tested in VOICE.12 Concentrations of TFV-DP remained high in tissues even after ring removal, providing the basis for a pharmacologically forgiving method. Plasma mean concentrations of LNG were >300 pg/ml within 1 hour of ring insertion.

Protocol A15-138, a randomized, placebo-controlled study, will extend the use of the IVR product to either 90 days of continuous use or 3 separate periods of 28 days of use with a 3 day washout between 28 day periods. The 3 day ring-free interval will add information on the impact of ring removal on LNG PK & PD and TFV PK. we will also evaluate bleeding patterns and acceptability by addressing differences with continuous versus monthly use.

1. Trial purpose, Objectives and Endpoints
   1. Purpose

The purpose of this trial is to assess the safety, pharmacokinetics (PK), and pharmacodynamics (PD) of the TFV/LNG IVR.

- 1. Objectives
     1. Primary Objective: Safety
- Evaluate genital and systemic safety of the TFV/LNG IVR during and after 90 days of continuous use or 90 days of interrupted use (3x28 days)
  - 1. Secondary Objectives: Pharmacokinetics (PK) and Pharmacodynamics (PD)
- Evaluate PK of TFV
- Evaluate PK of LNG
- Evaluate PD surrogate markers of contraceptive efficacy of LNG
- Evaluate PD surrogate markers of anti-HIV-1 and anti-HSV-2 efficacy of TFV in CV fluid
  - 1. Secondary Objective: Bleeding Patterns
- Compare bleeding patterns by regimen and by product group
  - 1. Secondary Objective: Forgiveness
- Evaluate forgiveness of TFV and LNG
  - 1. Secondary Objective: Acceptability
- Examine user preferences for IVR attributes and user attitudes, beliefs, and experiences towards IVR use by regimen and by product use
  - 1. Secondary Objective: Adherence
- Assess use of IVR during study period
  - 1. Exploratory Objectives: Pharmacodynamics (PD)
- Evaluate PD surrogate markers of anti-HIV-1 and anti-HSV-2 efficacy of TFV in rectal fluid
- Evaluate PD surrogate markers of anti-HSV-2 efficacy of TFV in CV tissue, as possible
- Assess qualitative measure of TFV in a vaginal swab
  - 1. Exploratory Objectives: Adherence
- Assess product use through returned IVRs by spectroscopic or other analytical methods
- Compare self-report of IVR removal scale to objective biomarkers of IVR use
- Assess baseline user characteristics as predictors of adherence
  1. Endpoints
     1. Primary Endpoints: Safety
- Treatment-emergent adverse events (TEAEs)
- Changes in serum chemistries, lipids, and complete blood count (CBC)
- Development of cervicovaginal (CV) ulcerations, abrasions, edema, and other findings as assessed by naked eye visualization of the CV epithelium
- Changes in soluble markers of innate mucosal immunity and inflammatory response in *CV fluid* [e.g., IL-1α, IL-6, IL-10, TNFα, RANTES, MIP-1α, IP-10, GM-CSF, IL-8, IL-1RA, SLPI, and BD2]
- Changes in HIV-1 target immune cell phenotype (e.g., CD45, CD68, CD4, and CD1a) and HIV-1 activation/proliferation markers (e.g., CD38, CCR5, HLA-DR, Ki67) in *CV tissue*
- Changes in hydrogen peroxide-secreting Lactobacilli concentration and other endogenous vaginal bacteria by quantitative PCR
- Microbial growth on returned IVRs
  - 1. Secondary Endpoints: Pharmacokinetics of TFV and LNG
- TFV concentrations in *plasma*, *CV fluid*, *rectal fluid*, and *CV tissue*
- TFV-DP concentrations in *CV tissue*
- LNG concentrations in *serum*
- Residual drug (TFV and LNG) in returned IVRs
  - 1. Secondary Endpoints: Pharmacodynamics of LNG

Surrogates of contraceptive efficacy:

- Cervical mucus assessment
  - Cervical mucus quality (score of >10)
  - Sperm migration on the Simplified Slide test
- Ovulation by serum progesterone (P4)
- Effect on follicular development by serum estradiol concentration
  - 1. Secondary Endpoints: Pharmacodynamics of TFV
- Anti-HIV-1 activity in *CV* *fluid*
- Anti-HSV-2 activity in *CV* *fluid*
- Comparison of HIV-1 ex vivo infection in *CV tissues* (EVMS only) at baseline and after 90 days of IVR use
  - 1. Secondary Endpoints: Bleeding Patterns
- Participant self-report of bleeding
  - 1. Secondary Endpoints: Forgiveness
- Decay of LNG during 3-day periods of non-use in interrupted regimen, and after 90 days of IVR use
- Decay of TFV during 3-day periods of non-use in interrupted regimen, and after 90 days of IVR use
  - 1. Secondary Endpoints: Acceptability
- Responses to key questions on acceptability and psychosocial questionnaire(s) (all participants), and feedback during in-depth interviews (subset of participants)
  - 1. Secondary Endpoints: Adherence
- Number and percentage of participants with Discontinuations/Expulsions/Removals by self-report
  - 1. Exploratory Endpoints: Pharmacodynamics of TFV
- Anti-HIV-1 activity in *rectal fluid*
- Anti-HSV-2 activity in *rectal fluid*
- Comparison of HSV-2 ex vivo infection in *CV tissues* (EVMS only) at baseline and after 90 days of IVR use, as possible
- Qualitative measure of TFV in a vaginal swab
  - 1. Exploratory Endpoints: Adherence
- Presence or absence of spectroscopic pattern signatures or other analytical measures of drug or placebo products (EVMS only)
- Characterization of returned IVRs (active and placebo) via objective IVR biomarkers (e.g., residual glycerin content and bioassay) and residual drug (TFV and LNG), as feasible
- Correlation of IVR removal scale factors and objective biomarkers of IVR use
- Correlation of baseline user characteristics and objective biomarkers of IVR use

1. Investigational Plan
   1. Overall Study Design

This will be a Phase I, randomized, placebo-controlled, parallel study. A total of 60 eligible participants will be randomized in a 4:4:1:1 ratio to study arm (see Table below), where Continuous arms will use the assigned IVR for 90 days continuously, and where Interrupted arms will use the assigned IVR for 3 periods of 28 days with 3 IVR-free days in between

Table 3: Randomization Assignments

| Arm | N | Dose | Regimen |
| --- | --- | --- | --- |
| 1 | 24 | TFV/LNG IVR (8-10mg/20μg) | 90 Days (Continuous) |
| 2 | 24 | TFV/LNG IVR (8-10mg/20μg) | 3x28 Days (Interrupted) |
| 3 | 6 | Placebo | 90 Days (Continuous) |
| 4 | 6 | Placebo | 3x28 Days (Interrupted) |

**Visit 1 (Screening):** All women will undergo a screening visit to determine the presence of exclusionary criteria to confirm that they are eligible to continue in the study.

**Visit 2 (Confirmation of ovulation):** Participants will undergo testing on cycle day 21 to confirm ovulation (P4 level of ≥3.0 ng/ml), and, if confirmed, will proceed to Visit 3.

**Visit 3 (Baseline and Randomization)**: On cycle day 24, participants will be asked to respond to psychosocial acceptability questions. Cervicovaginal (CV) fluid will be collected for semen testing and CV biopsies will be collected for target immune cells and HIV-1 activation/proliferation markers, and HIV-1 and HSV-2 ex-vivo infectivity (EVMS only). Participants will be randomized to study arm and will also receive a random time point assignment at this visit for post-IVR insertion sample collection at Visit 5.

**Visit 4 (IVR Insertion):** On cycle day 6, baseline samples for blood (TFV/LNG/SHBG/estradiol/P4), CV and rectal fluid for anti-HIV-1 and anti-HSV-2 activity, CV fluid for semen testing, soluble markers, and microflora (qPCR) will also be collected. Initiation of IVR will occur. After IVR insertion, blood samples for LNG/SHBG PK will be collected at four time points (1, 2, 4, and 8 hours), blood for TFV PK will be collected at 8 hours, and CV fluid for TFV PK will be collected at 2 and 8 hours.

**Visit 5 (Post insertion sample collection):** Based on randomized assignment to 24, 48, or 72 hours after IVR insertion, blood for TFV/LNG/SHBG, CV fluid and rectal fluid for TFV, and CV tissue for TFV/TFV-DP concentrations will be collected.

**Visits 6, 7, 9, 10, 12, 15, 16, 18, 19, 21, 24, 25, 27, 28, and 30 (P4/Estradiol):** Blood for P4 and estradiol concentrations will be collected approximately twice a week throughout IVR use, and cervical mucus will be collected once during each month of IVR use, according to estradiol/P4 concentrations. At Visit 27 (if not done at Visit 26), participants will receive a random time point assignment for post IVR removal sample collection at Visit 32.

Table 4: Guidelines for Cervical Mucus Collection

| Estradiol concentration | Cervical Mucus Collection |
| --- | --- |
| Is between 75 - 150 pg/ml | The participant will be asked to come in at their next scheduled visit for cervical mucus collection. Participants should be instructed to abstain from vaginal/anal intercourse/activity for 48 hours prior to cervical mucus collection, as possible. |
| Is >150 pg/ml | The participant will be asked to come in ideally within 24 hours of results being known for cervical mucus collection. Collection can take place the same day that results are known, per investigator discretion. |
| Does not reach 75 pg/ml before the end of each month (i.e., days 28, 59, and 90) of IVR use | Cervical mucus will be collected at the end of each month of IVR use (ideally days 28, 59, and 90 of IVR use). |
| Note that P4 must be <3ng/ml to collect cervical mucus. Once cervical mucus has been collected for that month, it should not be collected again until the next month unless there are extenuating circumstances (e.g., unviable sample), though estradiol and P4 samples will continue to be collected twice weekly. | |

Within the first month of IVR use, preferably at either Visit 6 or Visit 7, an in-depth interview will be conducted for a subset of participants.

**Visits 8, 17, and 26 (Months 1, 2, and 3):** Blood for TFV/LNG/SHBG/estradiol/P4, and CV fluid for TFV PK will be collected. Cervical mucus may also be collected at this visit (see Table above). At Visits 8 and 17, CV fluid will be collected for semen testing. At Visit 26 (if not done at Visit 27), participants will receive a random time point assignment for post IVR removal sample collection at Visit 32.

**Visits 11, 20, and 29 (Months 1, 2, and 3):** Blood for TFV/LNG/SHBG/estradiol/P4, and CV and rectal fluid for TFV PK will be collected. Cervical mucus may also be collected at this visit (see Table above). At Visits 11 and 29, CV fluid will be collected for anti-HIV-1 and anti-HSV-2 activity and semen testing. At Visit 29, rectal fluid will also be collected for anti-HIV-1 and anti-HSV-2 activity, as well as CV fluid for soluble markers.

**Visits 13, 22, and 31 (Months 1, 2, and 3):** Blood for TFV/LNG/SHBG/estradiol/P4 will be collected. Cervical mucus may also be collected at this visit (see Table above). Vaginal fluid will be collected for qualitative measure of TFV and CV fluid will be collected for microflora. At Visits 13 and 31, participants will also be asked to provide feedback about their experience with the IVR via a questionnaire. At Visits 13 and 22, participants in Arms 2 and 4 will remove the IVR in clinic under observation, and their IVR will be stored. At Visit 31, blood for CBC, serum chemistries, and fasting lipids will be collected; the IVR will be removed; CV tissue for TFV/TFV-DP concentrations, HIV-1 and HSV-2 ex-vivo infectivity (EVMS only), and immune cell and activation markers will be collected; an in-depth interview may be conducted for a subset of participants anytime starting at Visit 31 through one week after Visit 32.

**Visits 14 and 23:** Blood for TFV/LNG/SHBG/estradiol/P4 will be collected. Cervical mucus may also be collected at this visit (see Table above). Participants in Arms 2 and 4 will re-insert their [stored] IVR. For all participants, CV fluid for TFV PK and semen testing will be collected.

**Visit 32 (Post removal sample collection):** Based on randomized assignment to 48 hours, 72 hours, or 5 days after IVR removal, blood for TFV/LNG/SHBG and HIV-1, CV and rectal fluid for TFV PK; and CV tissue for TFV/TFV-DP concentrations will be collected.

**Follow Up Contact:** A final site contact will be scheduled for approximately 1 - 2 weeks after the last visit to ask the participant about adverse events (AEs) and concomitant medications (CMs). The participant will be exited unless there are symptoms that require follow up.

Participants will be given a guidebook in which to record AEs and CMs. Menses, bleeding/spotting, and vaginal/anal intercourse/activity will also be recorded in the guidebook.

Participants will abstain from the use of vaginal products (other than the study product and condoms) including tampons (except for menses), spermicides, lubricants, and douches for the whole study. Participants will abstain from vaginal and anal intercourse/activity starting 48 hours prior to cervical mucus collections, as possible, and 48 hours before Visits 4 and 29; and for 5 days after CV tissue collection. For sex acts during the study, non-spermicidal condoms must be used.

An interim analysis may be conducted to evaluate drug-release parameters to help inform product development.

Table 5: Schedule of Evaluations

|  | | | **Visit 1**  Screening | **Visit 2** | **Visit 3**  Baseline | **Visit 4** | **Visit 5**^2^  24, 48, or 72h post insertion | **E2/P4**^3^ | **Visits**  **8,17,26** | **Visits**  **11,20,29** | **Visits**  **13,22,31** | **Visits**  **14,23** | **Visit 32**^2^  48h, 72h, or 5d post removal | **Follow Up Contact** |
| --- | --- | --- | --- | --- | --- | --- | --- | --- | --- | --- | --- | --- | --- | --- |
| Informed consent | | | ✓ |  |  |  |  |  |  |  |  |  |  |  |
| Review eligibility criteria, interval history | | |  | ✓ | ✓ |  |  |  |  |  |  |  |  |  |
| Demographic info/medical history | | | ✓ |  |  |  |  |  |  |  |  |  |  |  |
| Height, weight, and blood pressure | | | ✓ |  |  |  |  |  |  |  | ✓  (V31) |  |  |  |
| Ask about compliance with study instructions/restrictions | | |  | ✓ | ✓ | ✓ | ✓ | ✓ | ✓ | ✓ | ✓ | ✓ | ✓ |  |
| Ask about AEs, CMs, and Menses | | |  |  |  | ✓ | ✓ | ✓ | ✓ | ✓ | ✓ | ✓ | ✓ | ✓ |
| Urine | Pregnancy test | | ✓ | ✓ | ✓ | ✓ |  |  |  |  |  | ✓ | ✓ |  |
| Blood | HSV-2, HIV-1, and HBsAG (with counseling) | | ✓ |  |  |  |  |  |  |  |  |  | ✓  (HIV-1 Only) |  |
|  | Safety (CBC, chemistries, lipids - fasting) | | ✓ |  |  |  |  |  |  |  | ✓  (V31) |  |  |  |
|  | PK TFV | |  |  |  | ✓  (Pre,8h) | ✓ |  | ✓ | ✓ | ✓ | ✓ | ✓ |  |
|  | PK LNG/SHBG | |  |  |  | ✓  (Pre,1,2,4,8h) | ✓ |  | ✓ | ✓ | ✓ | ✓ | ✓ |  |
|  | PD LNG (P4) | |  | ✓^1^ |  | ✓ |  | ✓ | ✓ | ✓ | ✓ | ✓ |  |  |
|  | PD LNG (Estradiol) | |  |  |  | ✓ |  | ✓ | ✓ | ✓ | ✓ | ✓ |  |  |
| Pelvic Exam and Genital Samples | Exam | Pelvic Exam (+bimanual at V1) | ✓ | (✓) | ✓ | ✓ | ✓ | ✓^5^ | ✓ | ✓ | ✓ | ✓ | ✓ | (✓) |
|  |  | Gram/Amsel’s at V1/ Amsel’s after V1 | ✓ | (✓) | (✓) | (✓) | (✓) | (✓) | (✓) | (✓) | (✓) | (✓) | (✓) | (✓) |
|  |  | GC, CT, TV testing | ✓ |  |  |  |  |  |  |  |  |  |  |  |
|  |  | Pap | (✓) |  |  |  |  |  |  |  |  |  |  |  |
|  |  | CV fluid (swab)  Semen |  |  | ✓ | ✓ |  |  | ✓  (V8,17) | ✓  (V11,29) |  | ✓ |  |  |
|  | PK | CV Tissue – Biopsy  TFV, TFV-DP |  |  |  |  | ✓ |  |  |  | ✓  (V31) |  | ✓ |  |
|  |  | CV Fluid (swab)  TFV |  |  |  | ✓  (2,8h) | ✓ |  | ✓ | ✓ |  | ✓ | ✓ |  |
|  |  | Rectal Fluid (sponge)  TFV |  |  |  |  | ✓ |  |  | ✓ |  |  | ✓ |  |
|  | Safety | CV Tissue – Biopsy  HIV-1 Target Imm. Cell/Activation Mrkrs |  |  | ✓ |  |  |  |  |  | ✓  (V31) |  |  |  |
|  |  | CV Fluid (CVL)  Soluble Markers of Mucosal Immunity |  |  |  | ✓ |  |  |  | ✓  (V29) |  |  |  |  |
|  |  | CV Fluid (swab)  Microflora |  |  |  | ✓ |  |  |  |  | ✓^9^ |  |  |  |
| Pelvic Exam and Genital Samples | PD | CV Tissue – Biopsy [EVMS site only]  HIV-1 Infectivity |  |  | ✓ |  |  |  |  |  | ✓  (V31) |  |  |  |
|  |  | CV Tissue – Biopsy [EVMS site only]  HSV-2 Infectivity |  |  | ✓ |  |  |  |  |  | ✓  (V31) |  |  |  |
|  |  | CV Fluid  anti-HIV-1/anti-HSV-2 |  |  |  | ✓ |  |  |  | ✓  (V11,29) |  |  |  |  |
|  |  | CV Fluid  Qualitative measurement of TFV |  |  |  |  |  |  |  |  | ✓ |  |  |  |
|  |  | Cervical Mucus  Quality/Sperm Migration |  |  |  |  |  | (✓)^4^ | (✓)^4^ | (✓)^4^ | (✓)^4^ | (✓)^4^ |  |  |
|  |  | Rectal Fluid  anti-HIV-1/anti-HSV-2 |  |  |  | ✓ |  |  |  | ✓  (V29) |  |  |  |  |
| Randomization to treatment and or time point post insertion/removal | | |  |  | ✓ |  |  | ✓^6^  (V27) | ✓^6^  (V26) |  |  |  |  |  |
| IVR Dispensation/Insertion | | |  |  |  | ✓ |  |  |  |  |  | ✓^7^ |  |  |
| IVR Removal (Complete IVR processing at V31) | | |  |  |  |  |  |  |  |  | ✓^7^ |  |  |  |
| Dispense, Review, or Collect Guidebook | | |  |  |  | ✓ | ✓ | ✓ | ✓ | ✓ | ✓ | ✓ | ✓ |  |
| Provide relevant instructions (e.g., vaginal/anal activity, IVR reinsertion, restrictions on product use and vaginal/anal activity) | | | ✓ | ✓ | ✓ | ✓ | ✓ | ✓ | ✓ | ✓ | ✓ | ✓ | ✓ |  |
| Acceptability/Psychosocial Questionnaire | | |  |  | ✓ |  |  |  |  |  | ✓  (V13,31) |  |  |  |
| IDI (completed by a subset of participants) ^8^ | | |  |  |  |  |  | ✓ |  |  | ✓  (V31) |  | ✓ |  |
| Study Exit | | |  |  |  |  |  |  |  |  |  |  |  | ✓ |

(✓) = if indicated

Pre = pre-IVR insertion

**NOTE:** directed physical exam, pelvic exam (possibly including removal of the IVR, per investigator discretion), dipstick/microscopy/culture and wet mount/pH will be performed at any visit per investigator discretion

^1^ P4 testing will be done at Visit 2 (d21±1). If the result is ≥ 3 ng/ml, the participant will return for Visit 3. If P4 <3 ng/ml at Visit 2, repeat testing will be scheduled (d23±1). If the repeat testing yields a serum P4 of ≥ 3 ng/mL, the participant will return for Visit 3. If the P4 at repeat Visit 2 is <3 ng/mL, the participant will be discontinued. Pregnancy test is not required for repeat Visit 2.

^2^ Per assigned time point.

^3^ Visits 6, 7, 9, 10, 12, 15, 16, 18, 19, 21, 24, 25, 27, 28, and 30. E2/P4 visits should ideally take place twice a week (approx. every 3-4 days), preferably M/Th or T/F. See Section 8.6 for more detail.

^4^ Cervical mucus will be collected once per month of IVR use (for quality/sperm migration) as follows:

- If estradiol concentration is between 75 - 150 pg/ml, the participant will be asked to come in at their next scheduled visit for cervical mucus collection.
- If estradiol concentration >150 pg/ml, the participant will be asked to come in ideally within 24 hours of results being known for cervical mucus collection. Collection can take place on the same day that results are known, per investigator discretion.
- If estradiol concentrations do not reach 75 pg/ml before the end of each month of IVR use, cervical mucus will be collected at the end of each month of IVR use (ideally days 28, 59, and 90 of IVR use). Please see other instructions in protocol Section 8.7.

^5^ Pelvic exams performed at Visits 10, 13, 15, 18, 21, 24, 27, and 30.

^6^ Randomization for post IVR removal sample collection time point at Visit 32 can occur at either Visit 26 or Visit 27.

^7^ Arms 2 and 4 will remove IVR at Visits 13 and 22 and reinsert IVR at Visits 14 and 23. Final IVR removal for all participants will take place at Visit 31.

^8^ First IDI will be scheduled to take place in the first month of IVR use, preferably at Visit 6 or Visit 7. Second IDI will be scheduled to take place anytime beginning at Visit 31 through one week after Visit 32.

^9^ Copan swabs will be collected at Visit 13 and Visit 22 and stored for possible analysis to assess for microflora during the study.

- 1. Anticipated Length of Study

Recruitment for the study is expected to take approximately 6 - 8 months. Each participant is expected to complete the study in 5 - 6 months. Therefore, the clinical portion of the study is expected to be completed in about 11 - 14 months.

- 1. Number of Subjects

Approximately 120 women may be consented and undergo assessment procedures in order to have approximately 60 women complete all visits. All participants who undergo baseline genital sampling will be included in the analysis. Reference Section 7.3.2 for target enrollment.

- 1. Treatment Assignment

Women will be randomized to one of four arms: TFV/LNG IVR (8-10mg per day/20μg per day) for 90 days (Continuous), TFV/LNG IVR (8-10mg per day/20μg per day) for 3x28 days (Interrupted), placebo IVR for 90 days (Continuous), or placebo IVR for 3x28 days (Interrupted).

- 1. Criteria for Study Termination

The study may be stopped if:

- Review of AEs shows an unexpected, significant, or unacceptable risk to the participants enrolled in the study or treatment arm. AEs will be monitored by the Medical Monitor and a decision to discontinue the study because of safety will take into account whether the AEs were product-related, were serious, and/or led to participant discontinuation.
- Six or more subjects experience similar related Grade 3 or 4 AEs
- The site has failed to enroll subjects at an acceptable rate
- The protocol requirements have not been adhered to
- Administrative reasons

1. Selection and Withdrawal of participants

The volunteers for this study will be recruited using a variety of methods, including through existing databases (as protected health information permits) and through advertisements (in electronic or local media outlets) which have been approved by the local Institutional Review Board (IRB).

Only women will be recruited since the endpoints require vaginal and cervical sampling. The NIH has mandated that children, defined as younger than 18 years old, be included in research trials when appropriate. This study meets “Justifications for Exclusion” criteria for younger children as set forth by the NIH (specifically, “insufficient data are available in adults to judge potential risk in children” and “children should not be the initial group to be involved in research studies”). As such, this study does not plan to enroll children. Efforts will be made by the sites to recruit participants so that the racial and ethnic characteristics of the subject population will reflect the demographics of the study sites. No selection criteria shall be based on race or ethnicity.

- 1. Subject Inclusion Criteria

Volunteers must meet all of the following criteria prior to baseline sampling at Visit 3.

1. Female, age 18-50 years, inclusive
2. General good health (by volunteer history and per investigator discretion) without any clinically significant systemic disease (including, but not limited to significant liver disease/hepatitis, gastrointestinal disease, kidney disease, thyroid disease, osteoporosis or bone disease, and diabetes) and with an intact uterus and cervix.
3. Currently having regular menstrual cycles (approximately 26-35 days) by participant report
4. History of Pap smears and follow-up consistent with standard clinical practice as outlined in the Study Manual or willing to undergo a Pap smear at Visit 1
5. Protected from pregnancy by one of the following:
   - Sterilization of either partner
   - Abstinence from penile-vaginal intercourse
   - In a same sex relationship
   - Consistent use of non-spermicidal condoms
6. Willing to abstain from use of vaginal products (other than the study product and condoms) including tampons (except for menses), spermicides, lubricants, and douches for the whole study
7. Willing to abstain from any vaginal and anal intercourse/activity starting 48 hours before cervical mucus collection, as possible, and 48 hours before Visits 4 and 29, and for 5 days after tissue collection
8. Vaginal and cervical anatomy that, in the opinion of the investigator, lends itself to easy genital tract sample collection
9. Negative urine pregnancy test
10. P4 ≥3 ng/ml
11. Willing to give voluntary consent and sign an informed consent form
12. Willing and able to comply with protocol requirements
    1. Subject Exclusion Criteria

Volunteers must meet none of the following criteria prior to baseline sampling at Visit 3.

1. BMI **≥** 30 kg/m^2^
2. History of hysterectomy
3. Currently pregnant or within two calendar months from the last pregnancy outcome. *Note: If recently pregnant, must have had at least two spontaneous menses since pregnancy outcome*
4. Use of any hormonal contraceptive method in the last 3 months (oral, transdermal, transvaginal, implant, or hormonal intrauterine contraceptive device)
5. Injection of Depo-Provera in the last 10 months
6. Use of copper IUD
7. Currently breastfeeding or having breastfed an infant in the last two months, or planning to breastfeed during the course of the study
8. History of sensitivity/allergy to any component of the study products, topical anesthetic, or to both silver nitrate and Monsel’s solution
9. Contraindication to LNG
10. In the last three months, diagnosed with or treated for any STI or pelvic inflammatory disease. *Note: Women with a history of genital herpes or condylomata who have been asymptomatic for at least six months may be considered for eligibility.*
11. Symptomatic bacterial vaginosis (BV)
12. Positive test for *Trichomonas vaginalis* (TV), *Neisseria gonorrhea* (GC), *Chlamydia trachomatis* (CT), HIV-1, or Hepatitis B surface antigen (HBsAg)
13. Known blood disorder, including deep vein thrombosis (DVT) and pulmonary embolism (PE), or those that could lead to prolonged or continuous bleeding with biopsy
14. Chronic or acute vulvar or vaginal symptoms (pain, irritation, spotting/bleeding, discharge, etc.)
15. Known current drug or alcohol abuse which could impact study compliance
16. Grade 2 or higher laboratory abnormality, per the Division of AIDS, National Institute of Allergy and Infectious Disease (DAIDS) Table for Grading the Severity of Adverse Events, or clinically significant laboratory abnormality as determined by the clinician
17. Systemic use in the last two weeks or anticipated use during the study of any of the following: corticosteroids, antibiotics, anticoagulants or other drugs known to prolong bleeding and/or promote clotting, antifungals, or antivirals or antiretrovirals (e.g. acyclovir, valacyclovir, Viread^®^, Atripla^®^, Emtriva^®^, or Complera^®^), or CYP3A4 inducers or inhibitors as detailed in the Study Manual (e.g., St. John’s Wort or erythromycin). *Note: Participants should avoid non-steroidal anti-inflammatory drugs (NSAIDs) except for treatment of dysmenorrhea during menses. Participants may use acetaminophen on an as-needed but not daily basis during the study.*^[[1]](#footnote-1)^
18. Participation in any other investigational trial with use of a drug/device within the last 30 days or planned participation in any other investigational trial with use of a drug/device during the study
19. History of gynecological procedures (including genital piercing) on the external genitalia, vagina, or cervix within the last 14 days
20. Abnormal finding on laboratory or physical examination or a social or medical condition in the volunteer which, in the opinion of the investigator, would make participation in the study unsafe or would complicate interpretation of data
    1. Participant Withdrawal
       1. Withdrawal Criteria

Participants who sign the informed consent and agree to participate in the study, but do not meet eligibility criteria will not undergo baseline sampling procedures (Visit 3) and will not continue in the study. No case report forms (CRFs) will be completed for participants who do not undergo baseline sampling.

Once a participant undergoes baseline sampling, she may be withdrawn from the study for the following reasons:

- Failure to follow protocol requirements that is judged severe enough by the investigator to significantly affect study outcomes
- Pregnancy or desire to become pregnant
- Use of exogenous hormones including systemic corticosteroid therapy
- Medical reasons, including diagnosis of an STI or symptomatic BV
- Personal reasons (participant request)
- Discontinuation of treatment arm, or of entire study
  - 1. Target Enrollment

The target is to have at least 60 women complete the study. Participants who are randomized but do not complete all sampling will contribute to the analysis as described in Section 14 and additional participants may be recruited until the target number of completers is achieved.

Participants who initiate product use but discontinue the study prior to completion may not re-enroll.

- - 1. Follow Up for Discontinued Participants

If a participant chooses to discontinue the study after the baseline visit, the site should ask the participant to return for study procedures, as relevant, and ask about medications taken and AEs since the last visit. If the participant undergoes biopsy procedures, the participant will be contacted by telephone approximately 1 - 2 weeks after the biopsy. The participant will be exited from the study during the last contact with the site.

1. Study Procedures

Prospective participants may be pre-screened by telephone or in person: the study will be explained, the inclusion/exclusion criteria reviewed, volunteers’ questions answered, and Visit 1 scheduled. As volunteers will be asked to fast at least 8 hours before Visit 1, verbal consent for this will be obtained during pre-screening and documented. Over approximately five to six months, each woman will have 32 scheduled visits and a follow up contact.

- 1. Visit 1: Screening

This visit will be scheduled for when the volunteer is not expecting to be menstruating. The following will take place to confirm the volunteer is eligible to continue in the study. Note that if the participant is not able to complete any of the study procedures at this visit (e.g., fasting blood draw), she may return for an unscheduled visit prior to Visit 2.

- The study and informed consent form will be reviewed and all volunteer questions will be answered. If the volunteer is eligible and wishes to participate in the study, she will be asked to sign an informed consent form. The Principal Investigator (PI) or designee will sign the form and offer a copy to the participant. Permission may be requested as part of the informed consent process for storage of the biologic samples obtained during the study for possible future testing, as allowed by the site.
- The participant will be interviewed to obtain medical history and demographic information.
- Height, weight, and blood pressure will be measured.
- If the participant’s history is significant for a medical condition, a directed physical exam will be performed.
- A urine specimen will be obtained for a urine pregnancy test and, with urinary symptoms, a clean-catch dipstick urinalysis will be performed (urine microscopy and culture will be performed as clinically indicated). If the pregnancy test is positive, the participant will be referred as necessary and will not continue in the study. If a urinary tract infection (UTI) is diagnosed, the participant will be treated, preferably with oral medication, and this visit will continue as scheduled.
- The participant will be informed that she will be asked to complete psychosocial questionnaires 3 different times during the study. She will also be asked whether she is willing to participate in 2 in-depth interviews, if selected.
- Blood samples will be collected for HSV-2^[[2]](#footnote-2)^, HIV-1, and HBsAg testing, as well as baseline fasting complete blood count (CBC), lipids, and serum chemistries. Participants who test HIV or HBsAg positive at screening are not eligible to continue in the study, and will be counseled and or given referrals, as needed.
- A pelvic (with bimanual) exam will be performed.
- A sample for wet mount/pH will be taken if symptomatic for vulvovaginal candidiasis (yeast infection) or bacterial vaginosis (BV). Symptomatic yeast infections will be treated, preferably with oral medication, and this visit will continue. If symptomatic BV is diagnosed, the participant will be treated and the visit will continue. The next visit will be scheduled for at least 2 weeks after completion of treatment and resolution of symptoms.
  - Specimens for *Trichomonas vaginalis*, *Neisseria gonorrhea*, and *Chlamydia trachomatis* will be collected. If positive, the participant will be treated or referred as necessary and will not be eligible to continue.
  - A Pap smear will be performed as indicated, consistent with current practice guidelines.
  - Gram stain will be performed to assess Nugent score.
- The participant will be encouraged to use non-spermicidal condoms for every sex act during the study.
- The participant will be reminded to:
  - Follow study instructions regarding vaginal/anal intercourse/activity and vaginal product use as described in Section 7.1.
  - Not use certain medications during the study (see Section 9.2).

There should be sufficient time between Visit 1 and Visit 2 to allow any needed lab results to become available. If any tests reveal that the participant does not meet inclusion/exclusion criteria, the participant will not continue to Visit 2.

- 1. Visit 2: Confirmation of Ovulation

Visit 2 will be scheduled for cycle day 21 (± 1 day) to confirm ovulation.

- An interval medical history will be collected, and the participant will be asked if she followed study instructions. If participant did not comply, she will be reminded of protocol requirements and the visit may continue or be rescheduled at the investigator’s discretion.
- Eligibility criteria will be reviewed. If any lab tests reveal that the participant is no longer eligible, the visit should not proceed and the participant will not be eligible to continue.
- If indicated, a directed physical exam, pelvic exam, dipstick urinalysis (and possibly microscopy and culture), and/or wet mount/pH will be performed.
- A urine specimen will be obtained for a urine pregnancy test to confirm that the participant is not pregnant. If pregnancy is diagnosed, the participant will be referred as necessary and will not be eligible to continue.
- For evaluation and management of genital findings and infections at this and all subsequent visits, see Table 11. This table will also provide guidance about whether to continue a participant based on diagnoses.
- Blood will be drawn for P4 concentration.

If ovulation is not confirmed at Visit 2 by a P4 of ≥3 ng/ml, Visit 2 will be repeated in the same menstrual cycle to obtain a follow-up P4; repeat Visit 2 will be scheduled on cycle day 23 (± 1 day) and a urine pregnancy test will not be required. If ovulation is not confirmed at repeat Visit 2, the participant will not be eligible to continue.

If ovulation is confirmed at Visit 2 or repeat Visit 2 by a P4 of ≥3 ng/ml, Visit 3 will be scheduled. If results are available and ovulation is confirmed, Visit 3 may occur on the same day as (repeat) Visit 2.

- The participant will be given reminders as described at Visit 1.
  1. Visit 3: Baseline and Randomization

This visit is scheduled on or before cycle day 24 (but not later than day 26) of the same menstrual cycle in which ovulation was confirmed. If more than eight weeks pass between Visit 1 and Visit 3, then some or all of Visit 1 procedures must be repeated, as described in the Study Manual.

- An interval medical history will be collected.
- The study staff will ask the participant if she followed study instructions. If participant did not comply, she will be reminded of protocol requirements and the visit may continue or be rescheduled for ideally her current or next menstrual cycle at the investigator’s discretion.
- If indicated, a directed physical exam, dipstick urinalysis (and possibly microscopy/cultures) and/or wet mount/pH will be performed.
- A urine specimen will be obtained for a urine pregnancy test to confirm that the participant is not pregnant, unless this visit takes place the same day as Visit 2. If pregnancy is confirmed, the participant will be referred as necessary and will not be eligible to continue.

If the participant meets all eligibility criteria and agrees to participate, she will continue in the study.

- The participant will be asked to respond to psychosocial acceptability questions.
- A pelvic exam will be performed and the following procedures will be completed:
  - Collection of CV fluid for semen swab testing. If the semen biomarker is positive, the participant will be reminded of protocol requirements and the visit will be rescheduled for ideally the next menstrual cycle.
  - Collection of CV biopsies for HIV-1 target immune cell and HIV-1 activation/proliferation markers (both sites), and for HIV-1 and HSV-2 infectivity (EVMS only).
  - Post-biopsy instructions will be reviewed with the participant.
- The participant will be randomized to one of four study arms:
  - TFV/LNG IVR 90-days (continuous)
  - TFV/LNG IVR 3x28 days (interrupted)
  - Placebo IVR 90-days (continuous)
  - Placebo IVR 3x28 days (interrupted)
- The participant will also receive a random time point assignment for sample collection time (24, 48, or 72 hours post IVR insertion). Study staff will provide participants with the next randomization code electronically generated. It is considered a protocol violation to randomize participants out of order.
- The participant will be instructed to contact the site and come in for an evaluation at any time during the study if she experiences any symptoms as described in Section 8.14.
- The participant will be reminded:
- To follow instructions regarding vaginal/anal activity and medications as described at Visit 1
  - To abstain from vaginal/anal intercourse/activity for 5 days after this visit, and starting again 48 hours before the next visit
  1. Visit 4: IVR Insertion

Visit 4 will be scheduled on day 6 (± 2 days) ideally of the first menstrual cycle after Visit 3.

- The study staff will ask the participant if she followed the instructions from the previous visit, and ask about AEs, CMs, and menses. If the participant did not follow study instructions, she will be reminded of protocol requirements and the visit will be rescheduled or continued per investigator discretion.
- A urine specimen will be obtained for a urine pregnancy test to confirm that the participant is not pregnant. If pregnancy is diagnosed, the participant will be referred as necessary and will be discontinued.
- If indicated, a directed physical exam, dipstick urinalysis (and possibly microscopy/cultures) and/or wet mount/pH will be performed at this and all subsequent visits.
- Blood will be collected for assessment of baseline TFV/LNG/SHBG/estradiol/P4 concentrations.
- A pelvic exam will be performed and the following procedures will be completed:
  - Naked eye visualization of the CV epithelium
  - Collection of CV fluid for:
    - Semen swab testing. If the semen biomarker is positive, the participant will be reminded of protocol requirements and visit will be rescheduled.
    - Anti-HIV-1 and anti-HSV-2 activity
    - Soluble markers of innate mucosal immunity and inflammatory response
    - Microflora
  - Collection of rectal fluid for:
    - Anti-HIV-1 and anti-HSV-2 activity
- The site will dispense the participant’s assigned IVR, based on randomization. The participant will insert the IVR into her vagina, under the supervision of clinic staff.

The following procedures will be performed post IVR insertion:

- Blood will be collected at 1, 2, 4, and 8 hours post insertion for LNG/SHBG and at 8 hours post-insertion for TFV concentrations.
- CV fluid will be collected at 2 and 8 hours post insertion for TFV concentrations.
- A participant guidebook (which will include instructions regarding post-biopsy care, vaginal restrictions, and visit information) will be reviewed and given to the participant to record any medications taken or symptoms experienced, sexual and vaginal activity, menses and other vaginal bleeding, and removal/expulsion and reinsertion of study product.
- The participant will be reminded:
- To follow instructions regarding IVR insertion/removal/expulsions (see Section 10.5)
- To contact the site as soon as possible during IVR use if she notices an obvious visual defect in the IVR (e.g., a failure of the weld between drug-containing segments, made apparent by the IVR no longer being a complete circle)
- To follow instructions regarding vaginal/anal activity and medications as described at Visit 1
- To complete the diaries in the guidebook and bring the guidebook to every visit
- To contact the site at any time during the study if she experiences any of the symptoms described in Section 8.14
- If a participant experiences any symptoms or signs attributable to use of the IVR, they may continue use at the investigator’s discretion as outlined in Section 13.5.
  1. Visit 5: Post-Insertion Sample Collection (24, 48, or 72 hours)

Visit 5 will be scheduled to allow for sample collection to take place approximately 24, 48, or 72 hours after initial IVR insertion, based on time point assignment.

- The study staff will ask the participant if she followed the instructions from the previous visit, and ask about AEs, CMs, menses, and review the guidebook. If the participant did not follow study instructions, she will be reminded of protocol requirements and the visit will continue.
- If indicated, a directed physical exam, dipstick urinalysis (and possibly microscopy/cultures) and/or wet mount/pH will be performed.
- Blood will be collected for TFV and LNG/SHBG concentrations.
- A pelvic exam will be performed and the following procedures will be completed:
  - Naked eye visualization of the CV epithelium (IVR can be briefly removed during exam to allow for optimal evaluation of the CV epithelium at any time during the study, per investigator discretion)
  - Collection of CV fluid for:
    - TFV concentration
  - Collection of rectal fluid for:
    - TFV concentration
  - Collection of CV biopsies for
    - TFV and TFV-DP concentrations
- Post-biopsy instructions will be reviewed with the participant.
- The participant will be given reminders as described at Visit 4, and will be reminded to abstain from vaginal/anal intercourse/activity for 5 days after this visit.
  1. Visits 6, 7, 9, 10, 12, 15, 16, 18, 19, 21, 24, 25, 27, 28, 30 (P4/Estradiol)

These visits should adhere as close to the visit schedule (every 3-4 days, ideally M/Th or T/F) as possible; however, to allow for flexibility (e.g., to avoid menses), sites may deviate from this schedule, making every attempt to have two visits a week, as evenly spaced as possible (see Section 8.7).

- The study staff will ask the participant if she followed the instructions from the previous visit, and ask about AEs, CMs, menses, and review the guidebook. If the participant did not follow study instructions, she will be reminded of protocol requirements and the visit will continue.
- If indicated, a directed physical exam, dipstick urinalysis (and possibly microscopy/cultures) and/or wet mount/pH will be performed.
- At these visits, blood for P4 and estradiol concentrations will be collected.
- Cervical mucus may be collected (see Section 8.7) for:
  - cervical mucus quality
  - sperm migration
- The participant will be given reminders as described at Visit 4.
- **Within Month 1 (preferably at Visit 6 or 7): Additional Procedures**
  - If selected, the participant will undergo an in-depth interview.
- **At Visits 10, 15, 18, 21, and 24: Additional Procedures**
  - A pelvic exam will be performed and the following procedures will be completed:
    - Naked eye visualization of the CV epithelium
- **At Visit 27: Additional Procedures**
  - A pelvic exam will be performed and the following procedures will be completed:
    - Naked eye visualization of the CV epithelium
  - If not done at Visit 26, the participant will receive a random time point assignment at this visit for Visit 32 sample collection (48 hours, 72 hours, or 5 days post IVR removal).
- **At Visit 28: Additional Procedures**
  - The participant will be reminded to abstain from vaginal/anal intercourse/activity starting 48 hours before the next visit.
- **At Visit 30: Additional Procedures**
  - A pelvic exam will be performed and the following procedures will be completed:
    - Naked eye visualization of the CV epithelium
  - The participant will be reminded to fast prior to Visit 31 for collection of blood for CBC, serum chemistries, and lipids.
  1. Guidelines for Cervical Mucus Collection

Guidelines for Cervical Mucus Collection

| Estradiol concentration | Cervical Mucus Collection |
| --- | --- |
| Is between 75 - 150 pg/ml | The participant will be asked to come in at their next scheduled visit for cervical mucus collection. Participants should be instructed to abstain from vaginal/anal intercourse/activity for 48 hours prior to cervical mucus collection, as possible. |
| Is >150 pg/ml | The participant will be asked to come in ideally within 24 hours of results being known for cervical mucus collection. Collection can take place the same day that results are known, per investigator discretion. |
| Does not reach 75 pg/ml before the end of each month (i.e., days 28, 59, and 90) of IVR use | Cervical mucus will be collected at the end of each month of IVR use (ideally days 28, 59, and 90 of IVR use). |
| Note that P4 must be <3ng/ml to collect cervical mucus. Once cervical mucus has been collected for that month, it should not be collected again until the next month unless there are extenuating circumstances (e.g., unviable sample), though estradiol and P4 samples will continue to be collected twice weekly. | |

- 1. Visits 8, 17, and 26 (Months 1, 2, and 3)

These visits will be scheduled to take place ideally on days 10, 42, and 73 of IVR use, respectively.

- The study staff will ask the participant if she followed the instructions from the previous visit, and ask about AEs, CMs, menses, and review the guidebook. If the participant did not follow study instructions, she will be reminded of protocol requirements and the visit will continue.
- If indicated, a directed physical exam, dipstick urinalysis (and possibly microscopy/cultures) and/or wet mount/pH will be performed.
- Blood will be collected for TFV/LNG/SHBG/estradiol/P4 concentrations.
- A pelvic exam will be performed and the following procedures will be completed at investigator discretion, based on assessment of previous biopsy sites.
  - Naked eye visualization of the CV epithelium
  - Collection of CV fluid for:
    - TFV concentrations
  - Cervical mucus may be collected (see Section 8.7) for:
    - cervical mucus quality
    - sperm migration
- The participant will be given reminders as described at Visit 4.
- **At Visits 8 and 17: Additional Procedures**
  - Collection of CV fluid for:
    - Semen swab testing
- **At Visit 26: Additional Procedures**
  - If not done at Visit 27, the participant will receive a random time point assignment at this visit for Visit 32 sample collection (48 hours, 72 hours, or 5 days post IVR removal).
  1. Visits 11, 20, and 29 (Months 1, 2, and 3)

This visit will be scheduled to take place ideally on days 21, 53, and 84 of IVR use, respectively.

- The study staff will ask the participant if she followed the instructions from the previous visit, and ask about AEs, CMs, menses, and review the guidebook. If the participant did not follow study instructions, she will be reminded of protocol requirements and the visit will continue.
- If indicated, a directed physical exam, dipstick urinalysis (and possibly microscopy/cultures) and/or wet mount/pH will be performed.
- Blood will be collected for TFV/LNG/SHBG/estradiol/P4 concentrations.
- A pelvic exam will be performed and the following procedures will be completed:
  - Naked eye visualization of the CV epithelium
  - Collection of CV fluid for:
    - TFV concentration
  - Cervical mucus may be collected (see Section 8.7) for:
    - cervical mucus quality
    - sperm migration
  - Collection of rectal fluid for:
    - TFV concentration
- The participant will be given reminders as described at Visit 4.
- **Visit 11: Additional procedures**
  - Collection of CV fluid for:
    - Semen swab testing
    - Anti-HIV-1 and anti-HSV-2 activity
- **Visit 29: Additional procedures:**
  - Collection of CV fluid for:
    - Semen swab testing
    - Anti-HIV-1 and anti-HSV-2 activity
    - Soluble markers of innate mucosal immunity and inflammatory response
  - Collection of rectal fluid for:
    - Anti-HIV-1 and anti-HSV-2 activity
  1. Visits 13, 22, and 31 (Months 1, 2, and 3)

This visit will be scheduled to take place ideally on days 28, 59, and 90 of IVR use, respectively.

- The study staff will ask the participant if she followed the instructions from the previous visit, and ask about AEs, CMs, menses, and review the guidebook. If the participant did not follow study instructions, she will be reminded of protocol requirements and the visit will continue.
- If indicated, a directed physical exam, dipstick urinalysis (and possibly microscopy/cultures) and/or wet mount/pH will be performed.
- Blood will be collected for TFV/LNG/SHBG/estradiol/P4 concentrations.
- Naked eye visualization of the CV epithelium (see *Visits 13, 22, and 31:* *Additional Procedures* below for details for specific visits)
- Vaginal fluid will be collected for qualitative measurement of TFV.
- Collection of CV fluid for:
  - Microflora^[[3]](#footnote-3)^
- Cervical mucus may be collected (see Section 8.7) for:
  - cervical mucus quality
  - sperm migration
- The participant will be given reminders as described at Visit 4.
- **Visits 13 and 22: Additional Procedures**
- IVRs will be removed to better visualize the cervicovaginal epithelium. IVRs for participants in Arms 1 and 3 will be reinserted immediately after examination procedures are performed and samples are collected.
- Arms 2 and 4 only: IVRs for participants in the interrupted groups will not be reinserted immediately; they will be processed, cleaned and stored for 72 hours as outlined in the Study Manual.
  - At Visit 13, the participant will also be asked to provide feedback via a psychosocial questionnaire.
- **Visit 31: Additional Procedures (Final IVR Removal)**
  - Height, weight, and blood pressure will be measured.
  - Blood will be collected for fasting complete blood count (CBC), serum chemistries, and lipids.
  - IVRs will be removed, and post-removal IVR processing will be immediately carried out as outlined in the Study Manual to allow for safety and adherence determinations (including residual drug concentrations, spectroscopy, IVR biomarkers, and microbial growth, as feasible).
  - A pelvic exam will be performed and the following procedures will be completed:
    - Naked eye visualization of the CV epithelium
    - Collection of CV biopsies for:
      - TFV and TFV-DP concentrations
      - HIV-1 target immune cell and HIV-1 activation/proliferation markers (both sites)
      - HIV-1 and HSV-2 infectivity (EVMS only)
- Post-biopsy instructions will be reviewed with the participant.
  - The participant will be asked to provide feedback via a psychosocial questionnaire (all participants). Selected participants may also undergo an in-depth interview anytime beginning at Visit 31 through one week after Visit 32.
  - The participant will be reminded to abstain from vaginal/anal intercourse/activity for 5 days after this visit.
  1. Visits 14 and 23 (Months 1 and 2)

This visit will be scheduled to take place ideally on days 32 and 63 of IVR use, respectively.

For Continuous arms (Arms 1 and 3), Visits 14 and 23 will be scheduled to take place approximately 72 hours after the end of Visits 13 and 22, respectively. For Interrupted arms (Arms 2 and 4), Visits 14 and 23 will be scheduled to take place 72 hours after IVR removal at Visits 13 and 22, respectively.

- The study staff will ask the participant if she followed the instructions from the previous visit, and ask about AEs, CMs, menses, and review the guidebook. If the participant did not follow study instructions, she will be reminded of protocol requirements and the visit will continue.
- If indicated, a directed physical exam, dipstick urinalysis (and possibly microscopy/cultures) and/or wet mount/pH will be performed.
- A urine specimen will be obtained for a urine pregnancy test to confirm that the participant is not pregnant. If pregnancy is diagnosed, the participant will be referred as necessary and will be discontinued.
- Blood will be collected for TFV/LNG/SHBG/estradiol/P4 concentrations.
- A pelvic exam will be performed and the following procedures will be completed:
  - Naked eye visualization of the CV epithelium
  - Collection of CV fluid for:
    - TFV concentration
  - Collection of CV fluid for:
    - Semen swab testing
  - Cervical mucus may be collected (see Section 8.7) for:
    - cervical mucus quality
    - sperm migration
- The participant will be given reminders as described at Visit 4.
- **Arms 2 and 4 only: Additional Procedures**
  - After PK/PD samples are obtained, the participant will be re-issued their stored IVR and will re-insert it into her vagina, under the supervision of clinic staff.
  1. Visit 32: Post-Removal Sample Collection (48 or 72 hours, or 5 days)

This visit will be scheduled to take place approximately 48 hours, 72 hours, or 5 days after IVR removal, based on time point assignment.

- The study staff will ask the participant if she followed the instructions from the previous visit, and ask about AEs, CMs, menses, and review the guidebook. The guidebook will be collected. If the participant did not follow study instructions, she will be reminded of protocol requirements and the visit will continue.
- If indicated, a directed physical exam, dipstick urinalysis (and possibly microscopy/cultures) and/or wet mount/pH will be performed.
- A urine specimen will be obtained for a urine pregnancy test to confirm that the participant is not pregnant. If pregnancy is diagnosed, the participant will be referred as necessary and will be discontinued.
- Blood will be collected for TFV/LNG/SHBG concentrations and HIV-1. Participants who test HIV positive will be counseled and given referrals, as needed.
- A pelvic exam will be performed and the following procedures will be completed:
  - Naked eye visualization of the CV epithelium
  - Collection of CV fluid for:
    - TFV concentration
  - Collection of rectal fluid for:
    - TFV concentration
  - Collection of CV biopsies for:
    - TFV and TFV-DP concentrations (both sites)
  - Post-biopsy instructions will be discussed with the participant.
- The participant will be reminded to abstain from vaginal/anal intercourse/activity for 5 days after this visit, and to contact the site at any time during the study if she experiences any of the symptoms described in Section 8.14.
- A follow-up contact will be scheduled for about 1 - 2 weeks after this visit.
  1. Follow-Up Call/Contact

The site will contact the participant approximately 1 - 2 weeks after Visit 32, or the last site visit, as appropriate. The participant will be asked about any AEs and CMs since her last visit. The participant will be exited from the study unless symptoms require further follow-up or she is appropriately referred.

- 1. Unscheduled Visits

The participant will be instructed to contact the site if she experiences fever, moderate to severe urogenital symptoms (e.g., genital burning, irritation, stinging, pressure, rash, itching, discharge, odor, urgency, dysuria, or hematuria), or if she experiences pelvic/lower abdominal pain, moderate to heavy menstrual bleeding (more than she would have during a normal period), or any bleeding (more than spotting) during the week following a biopsy. She should also contact the site if she expels or loses her IVR or otherwise needs additional supplies.

Unscheduled visits or procedures (e.g., pelvic exam, CBC) may be performed as indicated or otherwise deemed necessary by the investigator at any time during the study. The participant will be asked to come in for an evaluation, as indicated. Unscheduled visits that require examination or interview due to symptoms will be recorded on CRFs.

When an unscheduled visit occurs in response to an AE experienced by a study participant, study staff will assess the reported event clinically and provide or refer the participant to appropriate medical care, as necessary. All AEs will be evaluated and follow-up of any observed abnormalities will proceed according to Section 13.

- 1. Early Discontinuations

If the participant discontinues from the study prior to the final visit, the site, in consultation with CONRAD, may ask the participant to return for study procedures and/or sample collection, if she is willing and if relevant. At a minimum, the site may ask participants to return for safety assessments. If she undergoes biopsy procedures, she will be contacted approximately one week after the biopsy. She will be asked about medications taken and adverse events including symptoms since the last visit. She will be exited from the study during her last contact with the site.

1. Treatment of Participants
   1. Description of Study Drug

Table 6 provides a summary of the treatments to be administered, including the doses and route of administration.

Table 6: Investigational Product

|  | **Investigational Product** | **Reference Product** |
| --- | --- | --- |
| **Product Name:** | Tenofovir and Levonorgestrel | Placebo |
| **Unit Dose** | 8-10 mg/day TFV and 20 μg/day LNG | NA |
| **Dosage Form:** | Intravaginal ring | |
| **Route of Administration** | Vaginal | |
| **Physical Description** | Ring with outer diameter of 55.0 mm, consisting of two segments of polyurethane tubing with an outer cross-sectional diameter of 5.5 mm: a longer segment (135 mm) containing white to off-white TFV paste and a shorter one (34 mm) with a translucent LNG core | Ring with an appearance similar to that of the TFV/LNG IVR |
| **Manufacturer** | Particle Sciences (Bethlehem, PA USA) | |

- 1. Concomitant Medications

All concomitant medications will be recorded on CRFs for participants who have undergone baseline sampling.

Participants should not use the following medications during the study unless instructed by a clinician in which case they should inform the study staff:

Corticosteroids, antibiotics, anticoagulants or other drugs known to prolong bleeding and/or promote clotting, antifungals, antivirals, or antiretrovirals (e.g., acyclovir, valacyclovir, Viread^®^, Atripla^®^, Emtriva^®^, or Complera^®^) or CYP3A4 inducers or inhibitors as detailed in the Study Manual (e.g., St. John’s Wort or erythromycin) should not be used during the study. *Note: Participants should avoid non-steroidal anti-inflammatory drugs (NSAIDs) except for treatment of dysmenorrhea during menses. Participants may use acetaminophen on an as-needed but not daily basis during the study.^[[4]](#footnote-4)^*

Additionally, participants must refrain from the use of any hormonal contraceptive method months (oral, transdermal, transvaginal, implant, or hormonal intrauterine contraceptive device), injection of Depo-Provera, or the use of copper IUD during the study.

- 1. Treatment Compliance

The IVR will be inserted by the participant in the clinic at Visit 4, and will be used in accordance with the randomized assignment (i.e., for 90 days continuously, or for 3 periods of 28 days) allowing for per protocol removals.

The IVR will be removed in the clinic at Visit 31. Treatment compliance will be evaluated by self-report and by quantitative measures (see Section 15.2).

- 1. Randomization and Blinding

This is a prospective, randomized trial. To the extent possible, participants, laboratory staff, and investigators will be blinded to study treatment. Statistical/data analysts will be blinded to study treatment.

Participants will be randomized in a 4:4:1:1 ratio to:

Randomization Assignments

| Arm | N | Dose | Regimen |
| --- | --- | --- | --- |
| 1 | 24 | TFV/LNG IVR (8-10mg/20μg) | 90-Day (Continuous) |
| 2 | 24 | TFV/LNG IVR (8-10mg/20μg) | 3x28 Days (Interrupted) |
| 3 | 6 | Placebo | 90-Day (Continuous) |
| 4 | 6 | Placebo | 3x28 Days (Interrupted) |

1. Study DRug Materials and Management
   1. Description of Study Drug

The study drugs are supplied by CONRAD as IVRs packaged in individual foil pouches. Each participant will receive either an IVR containing 1.15 g TFV and 6.0 mg LNG or a placebo IVR. Each ring has a long white to off-white segment and a short translucent segment and has a 0.7 mm wall thickness, 5.5 mm outer cross-sectional diameter, and 55 mm outer diameter. The ring releases active pharmaceutical ingredients (APIs) of approximately 8-10 mg TFV per day (from the long segment) in combination with 20 µg LNG per day (from the short segment), or neither (placebo). The rings are formulated with the following excipients: modified starch (placebo only), polyurethane, glycerin, and water. The placebo IVR has a similar appearance and dimensions to the TFV/LNG IVR except that modified starch, which is non-eluting from the reservoir, is used to replace the TFV to provide a similar white filled tube appearance and the short segment consists of solid polyurethane.

The API TFV is supplied by Gilead Sciences, Inc. The API LNG is supplied by Industriale Chimica s.r.l. The formulation is manufactured for CONRAD under Good Manufacturing Practice (GMP) at Particle Sciences (Bethlehem, PA, USA).

- 1. Study Drug Packaging and Labeling

The site will be provided with and manage the distribution of the study products. Each site will be provided with sufficient study products for its designated number of participants. Supplies will be labeled, packaged, and distributed by Particle Sciences (Bethlehem, PA, USA).

The IVR will be packaged in an individual re-sealable foil pouch. Each pouch will include the following label:

Figure 2: Study Drug Label


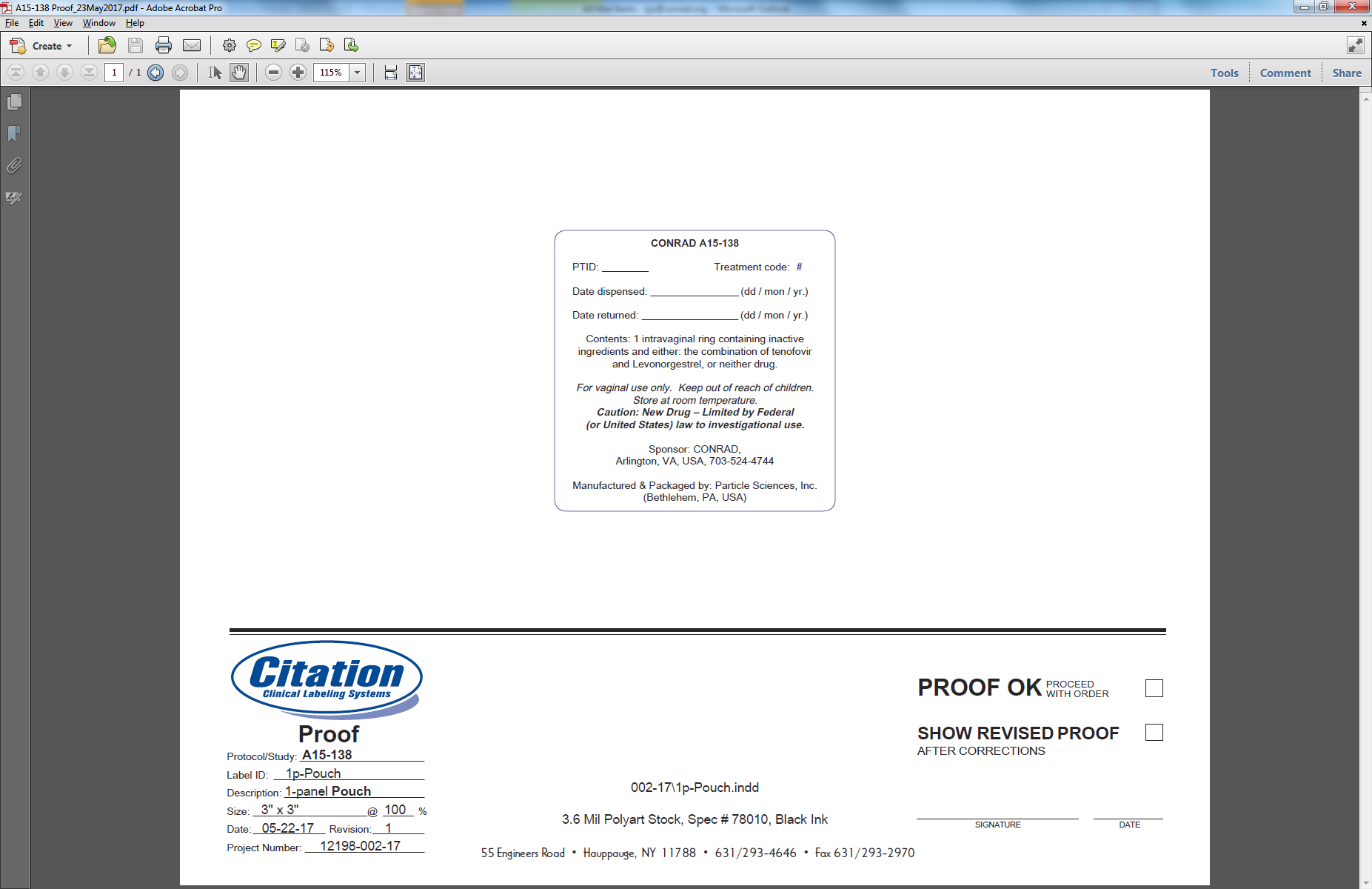


- 1. Study Drug Storage

Study product should be stored at room temperature (15° - 30°C) (59° - 86°F) in a locked cabinet or secure area in the clinic prior to dispensing.

- 1. Study Drug Preparation

These study products are ready to use and require no preparation other than packaging as described in Section 10.2.

- 1. IVR Insertion and Removal

Each participant will be randomized to an IVR regimen and will receive verbal and written instructions (participant guidebook) on insertion and removal, which will be done under supervision of clinic staff. For IVR insertion, the participant will insert the IVR in the clinic by pushing it in with a finger as deep as it will comfortably go.

The participant will be instructed not to remove the IVR but, if removal becomes necessary for some reason, or if the IVR is expelled, to try to re-insert the IVR and inform the site staff as soon as possible of expulsion/removal and reinsertion. The event should also be recorded in the guidebook. Participants should be strongly encouraged not to remove the IVR. If removal occurs, the IVR may remain outside of the vagina for up to two (2) hours without being considered a protocol violation and the IVR should be cleaned prior to reinsertion. If reinsertion is not possible, the participant should put the IVR into a zip-lock type foil pouch that is provided by the site, reseal it, and bring it to the clinic at her next visit.

For IVR removal, the participant will remove the IVR by hooking her index finger through the ring and gently pulling downward and forward to remove the ring to pull it out.

**For Arms 2 and 4:** At Visits 13 and 22, the removed IVR will be cleaned and stored in the clinic until the participant returns at the next visit for reinsertion. At Visits 14 and 23, the IVR will be reissued for reinsertion.

The date and time of any interim removals/expulsions and reinsertions by the participant will be recorded in the participant guidebook.

- 1. Study Drug Accountability

IVR dispensation and return will be recorded in a drug accountability log at the site.

- 1. Study Drug Handling and Disposal

All study product disposition (including receipt, dispensation, undispensed and returned study product) will be recorded by the site and reconciled by the clinical research associate (CRA). After reconciliation, the sponsor will provide instruction regarding disposal, storage, or return of product.

1. Assessment of Endpoints
   1. Pharmacokinetics
      1. Pharmacokinetics: Blood Sample Collection

Blood samples will be drawn to assess PK of TFV and LNG/SHBG as detailed below:

Table 7: PK: Blood Sample Collection

|  | **TFV: 8-10 mg and LNG: 20 μg** |
| --- | --- |
|  | **Visits** |
| Plasma: TFV | Visit 4: Pre-insertion and 8h post-insertion  Visits 5, 8, 11, 13, 14, 17, 20, 22, 23, 26, 29, 31, 32 |
| Plasma: LNG/SHBG | Visit 4: Pre-insertion and 1, 2, 4, and 8h post insertion  Visits 5, 8, 11, 13, 14, 17, 20, 22, 23, 26, 29, 31, 32 |

At Visit 5, samples will be collected per randomization time point (24, 48 or 72 hours post-IVR insertion).

At Visit 32, samples will be collected per randomization time point (48 or 72 hours, or 5 days post-IVR removal).

At Visits 13, 22, and 31 samples will be collected pre-IVR removal.

- - 1. Pharmacokinetics: Genital and Rectal Sample Collection

Genital samples will be collected to assess PK as detailed below:

Table 8: PK: Genital and Rectal Sample Collection

|  | **TFV: 8-10 mg and LNG: 20 μg** |
| --- | --- |
|  | **Visits** |
| CV Fluid: TFV^1^ | Visit 4: 2 and 8h post-insertion  Visits 5, 8, 11, 14, 17, 20, 23, 26, 29, and 32 |
| Rectal Fluid: TFV | Visits 5, 11, 20, 29, and 32 |
| CV Tissue: TFV and TFV-DP | Visits 5, 31, and 32 |

^1^ For Arms 2 and 4: At Visits 14 and 23, samples will be collected pre-IVR insertion.

- 1. Assessment of Pharmacodynamics
     1. Pharmacodynamics: Blood Sample Collection

Blood samples will be drawn to assess PD as detailed below:

Table 9: PD: Blood Sample Collection

|  | **TFV: 8-10 mg and LNG: 20 μg** |
| --- | --- |
|  | **Visits** |
| Plasma: P4^1^ | Visits 2, 4, and 6 - 31 |
| Plasma: estradiol | Visits 4, and 6 - 31 |

^1^ P4 will be drawn at Visit 2. If the result is < 3 ng/ml, the participant will return for repeat Visit 2 in the same menstrual cycle on day 23±1. If repeat Visit 2 does not confirm ovulation, the participant will be discontinued. If P4 ≥3 ng/ml at Visit 2 or repeat Visit 2, Visit 3 will be scheduled.

- - 1. Pharmacodynamics: Genital and Rectal Sample Collection

Genital samples will be taken to assess PD as detailed below:

Table 10: PD: Genital and Rectal Sample Collection

|  | **TFV: 8-10 mg and LNG: 20 μg** |
| --- | --- |
|  | **Visits** |
| CV Fluid: anti-HIV-1/anti-HSV-2 | Visits 4, 11, and 29 |
| CV Fluid: qualitative measurement of TFV | Visits 13, 22, and 31 |
| Rectal Fluid: anti-HIV-1/anti-HSV-2 | Visits 4 and 29 |
| Cervical Mucus: Quality/Sperm Migration^1^ | Visits 6 - 31 |
| CV Tissue: HIV-1-infectivity (EVMS only) | Visits 3 and 31 |
| CV Tissue: HSV-2-infectivity (EVMS only) | Visits 3 and 31 |

^1^ See Section 8.7

1. Assessment of Safety
   1. Medical History

A medical history will be completed at Visit 1. Height, weight, and blood pressure will be measured at Visits 1 and 31. Medical history will be updated at Visits 1, 2, and 3, and as applicable.

As part of AE data collection, participant AEs will be assessed at each study visit starting after baseline sample collection at Visit 3; participant report of symptoms will be documented and may result in follow-up assessments.

- 1. Physical and Pelvic Examination

A directed physical exam will be performed at each study visit, if indicated, with changes on physical exam documented.

Pelvic exams will be performed to assess safety. Findings will be managed as outlined in Table 11. Biopsy areas will be checked for healing at applicable visits and as indicated.

Table 11: Safety Management Plan for Genital Findings

| **Finding** | **Management** |
| --- | --- |
| Vaginal bleeding | The source of the bleeding should be evaluated. Bleeding from the cervical os should be evaluated per standard of care. If the bleeding is from a previous biopsy site or a deep epithelial disruption, hemostasis should be attempted via direct pressure. Silver nitrate or Monsel’s solution may also be applied. If adequate hemostasis is not obtained with these measures, electrocautery or suturing may be used. Samples may be collected per investigator discretion.  Product use may continue at investigator discretion and per Section 13.5. |
| Epithelial findings by naked eye visualization:   - Erythema, edema, petechiae, ecchymoses (epithelium intact by definition) – includes cervicitis - Peeling (superficial epithelial disruption, by definition) - Ulcers, abrasions, lacerations with superficial or deep epithelial disruption | **If the finding is bleeding** (which makes it deep epithelial disruption by definition), it should be managed as described under “Vaginal bleeding,” above.  **For all other findings**: If the area is small (≤3mm at the widest point), samples may be collected or the visit may be rescheduled, per investigator discretion. If the finding is >3 mm with epithelial disruption, consideration should be given to stopping product use and seeing the participant in unscheduled visits until the finding is resolved or resolving.  Product use may continue at investigator discretion and per Section 13.5. |
| Abnormal vaginal discharge | A wet mount with KOH and pH should be done, and NAAT for GC/CT/TV should be strongly considered. If an infection is diagnosed, it should be managed as described under “Epithelial findings” above. |
| Findings suspicious for infection | **If an infection is suspected (e.g., due to erythema, vesicles, concomitant discharge, etc.):**  A wet mount with KOH and pH, NAAT for neisseria gonorrhea, chlamydia trachomatis, trichomoniasis vaginalis (GC/CT/TV), and other tests may be done as indicated.   - If candidiasis, symptomatic BV*, or urinary tract infection (UTI) is diagnosed at **Visit 2**, the participant may be treated and Visit 2 rescheduled. - If candidiasis, symptomatic BV*, or UTI is diagnosed at **Visit 3**, the participant may be treated and Visit 2 and 3 rescheduled. - **At Visit 4 (pre-IVR insertion):** If candidiasis, UTI, or symptomatic BV* is diagnosed, the participant may be treated and Visit 4 rescheduled. - **After IVR insertion at Visit 4:** If candidiasis, UTI, or symptomatic BV is diagnosed, the participant may be treated and the visit may continue. Note: If symptomatic BV is diagnosed, the site should contact CONRAD, ideally during the visit to determine whether the participant may continue in the study. - If symptomatic GC/CT/TV or genital herpes simplex is diagnosed at any visit, the participant should be treated and discontinued.   * If the participant was diagnosed with symptomatic BV at a previous visit and at this visit, she will be treated and discontinued. Participants can only be treated once for BV prior to using study product. |

- 1. Laboratory Assessments
     1. Blood Samples

HIV-1 and HBsAg tests (with counseling) will be performed at Visit 1. An additional HIV-1 test (with counseling) will be performed at Visit 32. CBC, lipids (fasting), and serum chemistry will be measured at Visit 1 (the baseline analysis for the study) and at Visit 31. Changes will be assessed for safety. HSV-2 serology testing will be performed at Visit 1 for analysis only.

- - 1. Genital Samples

Genital swabs will be taken at baseline to test for the presence of Trichomonas, Chlamydia, and Gonorrhea. Additional samples will be taken to test for other infections if the participant is symptomatic at any visit.

Genital samples will be taken to assess PK as detailed above and to assess safety as detailed below.

Table 12: Safety: Genital Assessments

|  | **TFV: 8-10 mg and LNG: 20 μg** |
| --- | --- |
|  | **Visits** |
| CV Fluid: Microflora | Visits 4, 13*, 22*, and 31 |
| CV Fluid: Semen Swab | Visits 3, 4, 8, 11, 14, 17, 23, and 29 |
| CV Fluid: Soluble Markers of Mucosal Immunity | Visits 4 and 29 |
| CV Tissue: HIV-1 Target Immune Cells/Activation Markers | Visits 3 and 31 |

* Copan swabs will be collected at Visit 13 and Visit 22 and stored for possible analysis to assess for microflora during the study.

- - 1. Pregnancy

Participants must be protected from pregnancy as described in Section 7.1 A pregnancy test is required at Visits 1, 2, 3, 4, 14, 23 and 32 for all participants. A pregnancy test will be done at other visits, if indicated. If pregnancy is diagnosed, the participant will be referred as necessary and will be discontinued.

1. Adverse and Serious Adverse Events
   1. Definition of Adverse Events (AE)

An AE is any untoward medical occurrence associated with the use of an investigational product in humans, whether or not considered product related. An AE can therefore be any unfavorable and unintended sign (including an abnormal laboratory finding), symptom, or disease temporally associated with the use of an investigational product, whether or not considered related to the investigational product. Pre-existing events that increase in frequency or severity in nature during or as a consequence of use of an investigational product in human clinical trials will also be considered as AEs. Any AE with an onset date after the first date of study product administration is considered to be treatment-emergent.

All AEs with onset at or after baseline sampling, whether or not related to the study product, will be recorded on eCRFs provided by CONRAD.

According to 21 CFR 312.32 “IND Safety Reports” the following definitions of terms apply to AEs occurring in clinical studies involving drugs.

- - 1. Suspected Adverse Reaction

A suspected adverse reaction is “any AE for which there is a reasonable possibility that the study product caused the event.” An AE is considered to be a suspected adverse reaction only if there is evidence to suggest a causal relationship between the study product and the AE. Examples of causal relationship include:

- A single occurrence of an event that is uncommon and known to be strongly associated with product exposure (Examples, not necessarily applicable to this study, include angioedema, hepatic injury, Stevens-Johnson Syndrome)
- One or more occurrences of an event that is not commonly associated with product exposure, but is otherwise uncommon in the population exposed to the study product (e.g., tendon rupture)
- An aggregate analysis of specific events observed in a clinical trial (such as known consequences of the underlying disease or condition under investigation or other events that commonly occur in the study population independent of product exposure) that indicates those events occur more frequently in the product exposure group than in a concurrent or historical control group.
  - 1. Adverse Reaction

An adverse reaction is any AE caused by the study product. Adverse reactions are a *subset* of suspected adverse reactions where there is reason to conclude that the product caused the event.

- - 1. Serious Adverse Event (SAE) or Serious Suspected Adverse Reaction

An AE or suspected adverse reaction is considered “serious” if, in the view of either the investigator or the sponsor, it:

- Results in death
- Is immediately life-threatening
- Requires in-patient hospitalization or prolongation of existing hospitalization*
- Results in persistent or significant disability or incapacity
- Results in a congenital abnormality or birth defect
- Is an important medical event that may jeopardize the patient or may require medical or surgical intervention to prevent one of the outcomes listed above

*Note that per ICH SAE definition, hospitalization itself is not an AE, but is an outcome of the event. Thus, hospitalization in the absence of an AE is not regarded as an AE, and is not subject to expedited reporting. The following are examples of hospitalization that are not considered to be AEs:

- Protocol specified admission for procedure required by study protocol
- Admission for treatment of target disease of the study, or for pre-existing condition (unless it is a worsening or increase in frequency in hospital admissions as judged by the clinical investigator)
- Diagnostic admission (e.g., for a workup of an existing condition such as persistent pre-treatment lab abnormality)
- Administrative admission (e.g., for annual physical)
- Social admission (e.g., placement for lack of place to sleep)
- Elective admission (e.g., for elective surgery)

All SAEs starting from baseline sampling until the last contact with the site, whether or not they are related to the study product, must be recorded on CRFs provided by CONRAD. Reporting of SAEs is outlined in Section 13.4.

- - 1. Unexpected Adverse Event or Unexpected Suspected Adverse Reaction

An AE or suspected adverse reaction is considered “unexpected” if it is not listed in the investigator brochure or drug packet insert, is not listed at the specificity or severity that has been observed; or, if an investigator brochure is not required or available, is not consistent with the risk information described in the general investigational plan or elsewhere in the current application, as amended. For example (not necessarily applicable to this study), under this definition, hepatic necrosis would be unexpected (by virtue of greater severity) if the investigator brochure referred only to elevated hepatic enzymes or hepatitis. Similarly, cerebral thromboembolism and cerebral vasculitis would be unexpected (by virtue of greater specificity) if the investigator brochure listed only cerebral vascular accidents. “Unexpected,” as used in this definition, also refers to AEs or suspected adverse reactions that are mentioned in the investigator brochure as occurring with a class of drugs or as anticipated from the pharmacological properties of the drug, but are not specifically mentioned as occurring with the particular drug under investigation.

- - 1. Serious and Unexpected Suspected Adverse Reaction

The sponsor must report any suspected adverse reaction that is both serious and unexpected. The sponsor must report an AE as a suspected adverse reaction only if there is evidence to suggest a causal relationship between the product and the AE as described above.

- 1. Relationship to Study Product

An Investigator who is qualified in medicine must make the determination of relationship to the investigational product for each AE. Relatedness is an assessment made by a study clinician of whether or not the event is related to the study agent. Degrees of relatedness will be categorized according to current DAIDS-approved guidelines. Per the Manual for Expedited Reporting of Adverse Events to DAIDS (currently Version 2.0, January 2010), the relationship categories that will be used for this study are:

- **Related**: There is a reasonable possibility that the AE is related to the study product(s)
- **Not related**: There is not a reasonable possibility that the AE is related to the study product(s)
  1. Recording and Grading Adverse Events for Severity

Adverse events spontaneously reported by the participant and/or in response to an open question from the study personnel or revealed by observation will be recorded during the study at the clinical site. The AE term should be reported in standard medical terminology when possible. For each AE, an investigator, who is qualified in medicine, will evaluate and report the date of onset, date of resolution, severity, relatedness, action taken, serious outcome (if applicable), and whether or not it caused the patient to discontinue the study. Each AE will be graded for severity using the applicable DAIDS tables for grading the severity of adverse events (see table below), which can also be found at the main page [http://rsc.tech-res.com/safetyandpharmacovigilance](http://rsc.tech-res.com/safetyandpharmacovigilance/)/.

Table 13: Grading the Severity of Adverse Events

| Adult and Pediatric AEs (version 2.1, March 2017) | http://rsc.tech-res.com/clinical-research-sites/safety-reporting/daids-grading-tables |
| --- | --- |
| Female Genital AEs (version 1.0, November 2007) |  |
| Rectal Grading Table (version 1.0, November 2007; clarification May 2012) |  |

For clinical AEs NOT identified in the DAIDS AE Grading Tables, the following scale (as listed in the DAIDS Table for Grading the Severity of Adult and Pediatric AEs) should be used to grade severity:

- Mild: Symptoms causing no or minimal interference with usual social and functional activities
- Moderate: Symptoms causing greater than minimal interference with usual social and functional activities
- Severe: Symptoms causing inability to perform usual social and functional activities
- Potentially Life-Threatening: Symptoms causing inability to perform basic self-care functions OR medical or operative intervention indicated to prevent permanent impairment, persistent disability
- Death

It is important to distinguish between serious and severe AEs. Severity is a measure of intensity whereas seriousness is defined by the criteria under Section 13.1.3. An AE of severe intensity may not necessarily be considered serious.

Participants must be protected from pregnancy to participate in the study. Should a pregnancy occur after baseline sampling, it must be reported as soon as possible to CONRAD and recorded on CONRAD’s pregnancy CRF. The participant will be discontinued from the study and appropriate exit procedures will be followed. Note that pregnancy in itself is not regarded as an AE unless there is a suspicion that an investigational product may have interfered with the effectiveness of a contraceptive medication. If the participant has been exposed to study product, the course of the pregnancy should be followed until it has an outcome (e.g., spontaneous miscarriage, elective termination, normal birth, congenital abnormality). If the participant seeks care outside the site, every effort should be made to obtain her consent for the site to receive a copy of her medical records related to the pregnancy, its outcome, and the health of the neonate, if applicable.

Reports of congenital abnormalities/birth defects are SAEs. Spontaneous miscarriages should be reported and handled as AEs. Elective abortions without complications should not be reported as AEs.

- 1. Reporting Serious Adverse Events

Any SAE including those listed in the protocol, investigator brochure, or package insert must be reported to CONRAD within 24 hours of discovery. If there is any question whether the event meets the criteria for “serious” it should be reported anyway. In addition, a completed SAE form must be emailed to CONRAD as soon as possible. The investigator must complete, sign and date the SAE pages, verifying the accuracy of the information recorded on the SAE pages and the corresponding source documents.

Additional follow-up information, if required or available, should all be emailed to CONRAD within one business day of receipt and this should be completed on an SAE form and placed with the original SAE information and kept with the appropriate section of the study database and/or study file.

CONRAD will notify the FDA and all participating investigators (i.e., all investigators to whom CONRAD is providing the relevant drug under its IND(s) or under any investigator's IND) in an IND safety report of potentially serious risks, from clinical trials or any other source, as soon as possible, but in no case later than 15 calendar days after CONRAD determines that the information qualifies for reporting under CFR 312.32(c)(1) which includes serious and unexpected suspected adverse reactions, findings from other studies, findings from animal or in-vitro testing, and increased rates of occurrences of serious suspected adverse reactions. In each IND safety report, CONRAD will identify all IND safety reports previously submitted to FDA concerning a similar suspected adverse reaction, and will analyze the significance of the suspected adverse reaction in light of previous, similar reports or any other relevant information.

CONRAD will notify the FDA of any unexpected fatal or life-threatening suspected adverse reactions as soon as possible, but in no case later than 7 calendar days after CONRAD initially receives the information.

CONRAD will promptly investigate and follow up on all safety information it receives, with the cooperation of the investigator. Follow-up information relevant to an IND safety report will be submitted as soon as the information is available and will be identified as such, i.e., “Follow-up IND Safety Report.”

The investigator is responsible for complying with IRB requirements for AE reporting and supplying CONRAD with copies of such correspondence.

- 1. Temporary Product Hold/Permanent Discontinuation in Response to Adverse Events

All AEs are defined by the DAIDS Table for Grading the Severity of Adult and Pediatric Adverse Events, Version 2.1, March 2017; Addendum 1, Female Genital Table for Use in Microbicide Studies (Version 1.0, November 2007); and Addendum 3, Rectal Grading Table for Use in Microbicide Studies (Version 1.0, November 2007; Clarification dated May 2012).

- - 1. Grade 1 or 2

In general, a participant who develops a Grade 1 or 2 AE, regardless of relationship to study product, may continue product use. If the PI/designee opts to temporarily hold study product, CONRAD must be notified.

Follow-up testing for Grade 2 laboratory test results should be performed at scheduled study visits, as clinically relevant. More frequent testing may be performed at any time if required to properly monitor and/or manage participant safety, at the discretion of the PI/designee.

- - 1. Grade 3 and 4

A participant who develops a Grade 3 or a Grade 4 AE that is either urogenital or product-related should have the product withheld and CONRAD should be notified. If a participant develops a Grade 3 or Grade 4 AE that is not urogenital or product-related, the product may be withheld per PI discretion. If the product is withheld, the PI/designee must notify CONRAD.

If the product is withheld, the PI/designee must continue the product hold until a recommendation is obtained from CONRAD regarding whether product use may resume. If, in consultation with CONRAD, product use is resumed and the same AE recurs at the same (or worse) grade level at any time during the study, study product must then be permanently discontinued.

1. Statistics

An expanded statistical analysis plan (SAP) will be written and approved before study completion. Any decisions regarding data handling, exclusions from analysis, etc., made subsequent to the approval of this protocol will be documented in the SAP or final study report.

- 1. Sample Size Justification

Sample size for this Phase I study is primarily based on the size of similar studies and feasibility although statistical considerations are considered.

As a means to characterize the statistical properties of this study Table 14 shows the exact 2-sided 95% confidence intervals for the probability of an event based on a particular observed rate. For example, if none of the 24 participants in either of the TFV/LNG IVR study arms experiences a safety event, the 95% exact 2-sided confidence interval for the true rate of event is (0.0%, 14.2%). If we see 2 events, this changes to (1.0%, 27.0%).

Table 14: Exact Two-sided 95% Confidence Intervals Based on Observing a Particular Rate of Safety Endpoints for Groups of Size 24 and 48

| **Active Treatment Group** | **Observed Event Rate, Percentage (Fraction)** | **Confidence Interval (%)** |
| --- | --- | --- |
| One Active Group (N=24) | 0.0 (0/24) | (0.0, 14.2) |
|  | 4.2 (1/24) | (0.1, 21.2) |
|  | 8.3 (2/24) | (1.0, 27.0) |
| Pooled Active Groups (N=48) | 0.0 (0/48) | (0.0, 7.4) |
|  | 2.1 (1/48) | (0.1, 11.1) |
|  | 4.2 (2/48) | (0.5, 14.3) |

The primary aim of the study is to evaluate the safety of TFV/LNG IVR. Assuming a two-sided Fisher’s Exact test with α =.10 and 80% power, Table 15 provides the difference in the rates of safety events (proportion of females experiencing the safety event of interest) between TFV/LNG IVR and placebo that is detectable for a given rate in the placebo group. For example, if the true rate of a given toxicity endpoint in the combined placebo group is 8.3% (1 out of 12 females experiencing a safety event); the proposed sample size provides 80% power to detect a difference between one active group versus pooled placebo groups for safety endpoint rates greater than 50.0% (45.8% for pooled active groups versus placebo).

Table 15: Difference in the Observed Rates of Safety Events

| **True Rate in Pooled Placebo Groups, percentage (fraction)** | **Observed Rate in One Active Group (N=24) Detectable with 80% Power,**  **percentage (fraction)** | **Observed Rate in Pooled Active Groups (N=48) Detectable with 80% Power,**  **percentage (fraction)** |
| --- | --- | --- |
| 8.3 (1/12) | 50.0 (12/24) | 45.8 (22/48) |
| 16.7 (2/12) | 62.5 (15/24) | 58.3 (28/48) |
| 25.0 (3/12) | 70.8 (17/24) | 68.8 (33/48) |
| 33.3 (4/12) | 79.2 (19/24) | 75.0 (36/48) |
| 41.7 (5/12) | 87.5 (21/24) | 81.3 (39/48) |
| 50.0 (6/12) | 91.7 (22/24) | 87.5 (42/48) |

- 1. General Statistical Issues

All attempts will be made to avoid missing data. However, missing values will remain as missing, i.e., no attempt will be made to impute. In general, only observed values will be used in data analyses and presentations. The one exception to this is summarizing concentration data; values that are below the lower limit of quantification (LLOQ) will be handled as follows: pre-dose concentrations will be set to zero and post-dose concentrations will be set to one-half the value LLOQ. Otherwise, no imputation is anticipated. No transformation will be applied.

- 1. Analysis Populations

The Randomized Population (RP) includes all randomized participants.

The Treated Population (TP) is a subset of RP and will consist of all randomized participants with any IVR use. Treatment groups are defined by treatment received (i.e., allocation errors are taken into account).

The Evaluable Population (EP) is a subset of TP and will consist of all randomized participants with any IVR use and contributing at least some follow-up safety or PK/PD data. The Evaluable Population will be the primary analysis population for most objectives.

Additional populations or refinements to these definitions, if needed due to unanticipated circumstances, will be described in the SAP or statistical report.

A subset of the continuous and interrupted regimens (and by product group) will be selected by an unblinded statistician during the course of the study for in-depth interviews. Healthcare provider perspectives will also be collected regarding the study participant experiences.

- 1. Statistical Analysis

*Evaluation of Study Objectives:* Study objectives will be evaluated by clinical review of descriptive summaries and graphical displays.

Analysis of safety will include summaries by treatment group of the incidence of treatment-emergent adverse events and incidence of other findings from pelvic examinations (e.g., ulcerations, abrasions, edema, and bleeding). Differences in event rates between treatment groups will be evaluated as detailed in the full analysis plan. Changes from baseline in clinical laboratory endpoints, soluble markers of mucosal immunity, HIV-1 target immune cell and activation markers, and microflora bacteria concentrations will be summarized by treatment group and time point. Shift tables will be produced where relevant.

PK analysis will include descriptive statistics by time point of TFV concentrations for all plasma, tissue, and fluid sample types, in addition to LNG and SHBG concentrations from plasma samples and TFV-DP concentrations from CV tissue samples. PK parameters (e.g., C_max_, T_max_, AUC) of TFV, TFV-DP, and LNG will be estimated by non-compartmental analysis of the concentration time curve or mean (composite) concentration time curve as appropriate. Comparisons of TFV concentrations and select PK parameters of TFV concentration will be made between active [continuous TFV/LNG IVR (8-10mg/20μg) vs. interrupted TFV/LNG IVR (8-10mg/20μg)] treatment groups.

PD endpoints will be summarized using descriptive statistics by time point as well as changes from baseline. Estimates and 95% confidence intervals will be provided. Comparison between treatment groups may be performed for selected endpoints using an analysis of covariance (ANCOVA) model though it is acknowledged that the trial may not be powered to test hypotheses regarding treatment group differences. Analysis will include summaries of P4 and estradiol concentrations over time, HIV-1 and HSV-2 infectivity endpoints from CV tissue [p24, cumulative p24 (CUM; the summation of p24 values measured by Elisa from Day 0 to Day 21), AUC (area under the virus growth curve), and p24 and the soft endpoint (SOFT)] before and after treatment, anti-HIV-1/anti-HSV-2 activity in CV and rectal fluid, and qualitative measurement of TFV in CV fluid.

Other secondary and exploratory endpoints will be summarized by treatment group using descriptive statistics, as appropriate. Endpoints of quantitative adherence (acceptability questionnaire) will be largely descriptive and will be collected via a GCP compliant electronic survey system. IDIs will be audio-recorded, transcribed, and translated as necessary, for qualitative analysis.

We may explore correlations between IVR scales collected at Visits 3, 13 and 31 with biomarker adherence measures.

*Descriptive Statistics:* Categorical variables will be summarized by frequencies and percentages. Continuous variables will be summarized by means, standard deviations, medians, quartiles, minima and maxima. Summaries of PK concentrations will also include the geometric mean and geometric coefficient of variation.

*Imputation:* In terms of summarizing concentration data, values that are below the lower limit of quantification (LLOQ) will be handled as follows: pre-dose concentrations will be set to zero and post-dose concentrations will be set to one-half the value LLOQ. Otherwise, no imputation is anticipated. If needed, rules for imputing missing values will be created and will be documented in the SAP or statistical report.

*Control of Type I Error:* This Phase I study is descriptive in nature. Any p-values or confidence intervals around estimates of treatment differences will not be adjusted for multiplicity and are descriptive in nature. These statistics will be provided to guide clinical judgment; caution must be used as these statistics will not have a controlled Type I error.

*Definition of Baseline:* Baseline is defined as the non-missing value obtained closest to but prior to the first product use.

*Subject-Specific Listings:* Summary tables will be accompanied by subject-specific listings of data as needed.

- 1. Interim Analysis

An interim analysis may be conducted to evaluate drug-release parameters to help inform the direction of further clinical development (i.e., additional studies). The analysis details of any interim analysis will be described in the SAP.

1. management of Intercurrent Events
   1. Loss to Follow-up

If a participant fails to appear for a scheduled visit, at least three attempts to contact her should be made over the subsequent 30 days. These attempts should be documented in the participant’s study file. The final attempt must be a certified letter to the participant with return-receipt requested, or an outreach attempt. A copy of this letter or documentation of the final outreach attempt should be in her file. After these three attempts, no further efforts need to be made to find her, but her file should remain open until study closeout.

If the participant does not contact the clinic before the study is closed, her final disposition will be recorded at the time of study closeout, to indicate that she was lost to follow-up. The lost to follow-up designation cannot be made for any participant until study close out.

- 1. Protocol Adherence

Participants will be considered compliant with the study regimen if they use the IVR per protocol. See Section 9.3 (Treatment Compliance). Compliance can also be quantitatively evaluated from PK concentrations; therefore, the site should carefully record the date and time of each collection of PK samples. The time of sampling will be considered a protocol violation based on the time the first sample is taken (when multiple samples types are being collected at a time point); all samples should be collected within a 45 minute period. Sampling windows:

- ± 15 minutes outside of the 1 and 2 hour time points
- ± 30 minutes outside of the 4 and 8 hour time points
- ± 2 hours outside of the 24 hour time point
- ± 10 hours outside of the 48 and 72 hour time points
- ± 24 hours outside the 5 day time point

Blood samples for CBC/serum chemistries/lipids scheduled for collection at Visit 31 may be collected just prior to or shortly after IVR removal at the visit.

Visits should adhere as close as possible to the visit schedule. Sites should contact CONRAD if a visit will be missed or if two visits will overlap, to determine appropriate procedures. Missing visits or study procedures will be considered protocol violations.

- 1. Protocol Violations

If a protocol violation is required to protect the life or physical well-being of a participant in an emergency, it may be carried out without prior approval from CONRAD or the IRB. The investigator must, however, report the violation to CONRAD and the IRB as soon as possible, no later than 5 working days after the emergency occurred. CONRAD will in turn notify the FDA within 5 working days after receipt of the report.

Other violations from the protocol may not be carried out without prior approval from the IRB if the change involves the rights, safety, or welfare of participants; and without prior approval from CONRAD if the change involves the validity of the data, the study's scientific soundness or the rights, safety, or welfare of participants.

All protocol violations should be recorded in a protocol violation log provided by CONRAD.

- 1. Modification of Protocol

No modification of this protocol may be made without written approval of CONRAD.

1. Direct access to source data/documents
   1. Study Monitoring

CONRAD will be responsible for developing the monitoring plan and monitoring the study. A Site Visit Log will be maintained at the site in which all site monitoring and other study-related visits will be recorded.

Before an investigational site can enter a patient into the study, a representative of CONRAD may visit the study site to:

- Determine the adequacy of the facilities; and/or
- Discuss with the investigator(s) and other personnel their responsibilities with regard to protocol adherence, and the responsibilities of CONRAD or its representatives.

During the study, a clinical research associate (CRA) from CONRAD or representative will have regular contact with the clinical site, for the following reasons, including but not limited to:

- Provide information and support to the investigator(s)
- Confirm that facilities remain acceptable
- Confirm that the investigational team is adhering to the protocol, that data is being accurately recorded in the CRFs, and that investigational product accountability checks are being performed
- Confirm all volunteers have been properly consented
- Perform source data verification. This includes a comparison of the data in the CRFs with the patient’s medical records at the clinical site, and other records relevant to the study. This will require direct access to all original records for each patient (e.g., clinic charts).
- Record and report any protocol violations not previously sent to CONRAD
- Confirm AEs and SAEs have been properly documented on CRFs and confirm any SAEs have been forwarded to CONRAD and those SAEs that met criteria for reporting have been forwarded to the IRB

The CRA will be available between visits if the investigator(s) or other staff needs information or advice.

- 1. Audits and Inspections

Authorized representatives of CONRAD, a regulatory authority, an Independent Ethics Committee or an IRB may visit the site to perform audits or inspections, including source data verification. The purpose of a CONRAD audit or inspection is to systematically and independently examine all study-related activities and documents to determine whether these activities were conducted, and data were recorded, analyzed, and accurately reported according to the protocol, Good Clinical Practice (GCP) guidelines of the International Conference on Harmonization (ICH), and any applicable regulatory requirements.

The investigator may be subject to a field audit by FDA inspectors. This audit could occur while the study is in progress, several years after the study is completed, or when the data are under review by the FDA as part of the new drug approval process. All of the participants’ records and other study documentation must be filed and accessible on short notice (3-5 days) during the study and subsequent retention period. The investigator should contact CONRAD immediately if contacted by a regulatory agency about an inspection.

- 1. Institutional Review Board (IRB)

The Principal Investigator (PI) must obtain IRB approval for the investigation. Initial IRB approval, and all materials approved by the IRB for this study including the patient consent form and recruitment materials must be maintained by the Investigator and made available for inspection.

1. quality control and quality assurance

To ensure compliance with GCP and all applicable regulatory requirements, CONRAD may conduct a quality assurance audit. Please see Section 16.2 for more details regarding the audit process.

1. ethics
   1. Ethics Review

The final study protocol, including the final version of the informed consent form, must be approved or given a favorable opinion in writing by an IRB or IEC as appropriate. The investigator must submit written approval to CONRAD before he or she can enroll any patient/subject into the study. The study must be conducted in accordance with all conditions of approval by the IRB.

The PI is responsible for informing the IRB or IEC of any amendment to the protocol in accordance with local requirements. In addition, the IRB or IEC must approve all advertising used to recruit patients for the study. The protocol must be re-approved by the IRB or IEC upon receipt of amendments and annually, as local regulations require.

The PI is also responsible for providing the IRB with reports of any reportable serious adverse drug reactions from any other study conducted with the investigational product. CONRAD will provide this information to the PI.

Progress reports and notifications of serious adverse drug reactions will be provided to the IRB or IEC according to local regulations and guidelines.

- 1. Ethical Conduct of the Study

The study will be performed in accordance with ethical principles that have their origin in the Declaration of Helsinki and are consistent with ICH GCP, and applicable regulatory requirements. In addition, the PI will follow U.S. Department of Health and Human Services regulations regarding the Health Information Portability and Accountability Act (HIPAA 45, CFR 164). The PI will ensure that appropriate health care or referral is provided for the participants throughout the study.

- 1. Written Informed Consent
     1. Procedure for Obtaining Informed Consent

No volunteer may be admitted into this study until the PI (or designee) has obtained her legally effective informed consent. The PI (or designee) shall seek such consent only under circumstances that provide the prospective participant with sufficient opportunity to consider whether or not to participate in the study. Informed consent must be obtained without coercion, undue influence, or misrepresentation of the potential benefits or risks that might be associated with participation in the study.

Informed consent encompasses all oral and written information given to the volunteer about the study and the study materials. This includes the consent form signed by the participant, the instructions for use of study materials that are provided to the participant, recruitment advertising, and any other information provided to the participant. All such information that is given to the participant will be in a language that is understandable to her. The information will not include any language in which the participant is made to waive any of her rights or which releases or appears to release the PI, the PI’s institution, or CONRAD from liability for negligence.

Informed consent will be documented by the use of a written consent form that is signed by the participant and the PI (or designee). A copy of the consent form will be offered to each participant. The original signed consent form for each participant will be kept at the site. The consent form must include each of the basic and additional elements of informed consent described in 21 CFR Part 50.25 and must describe each of the risks or discomforts to the participant that have been identified by CONRAD as reasonably foreseeable. CONRAD will provide a sample consent form that meets these requirements. If the PI revises the sample consent form or develops a new one, the new or revised consent form should be submitted to CONRAD for review before it is submitted to the local IRB.

- - 1. Subject Confidentiality

The confidentiality of all subjects consented into this clinical study will be protected to the fullest extent possible. Subjects’ clinic records may be audited by CONRAD staff or other individuals authorized in writing by CONRAD to audit the study. However, study subjects will not be identified by name on any CRF, or on any other documentation sent to CONRAD or other organizations involved in this study, and will not be reported by name in any report or publication resulting from data collected in this study.

1. Data handling and recordkeeping
   1. Method of Data Capture

Clinical data will initially be recorded on source documents at the clinical site. The source documents, including signed informed consent forms, laboratory reports, and participant records, should be maintained at the site, and should be available for review during monitoring visits.

Information from the source documents will then be entered onto electronic CRFs (eCRFs), as outlined in the Study Manual and the data management plan. eCRFs will not be completed for participants who discontinue before baseline sampling.

Data from central laboratories will be entered into electronic data sets, provided or approved by CONRAD or designee, as appropriate.

Quantitative adherence (acceptability questionnaire) data will be collected via a GCP-compliant, professionally administered electronic survey system. IDIs will be audio-recorded, transcribed, and translated as necessary.

- 1. Inspection of Records

CONRAD will be allowed to conduct site visits to the clinical site for the purpose of monitoring any aspect of the study. The PI agrees to allow the CRA or designee to inspect the storage area, inventory, and accountability records for study product, subject charts and study source documents, and other records relative to study conduct.

- 1. Retention of Records

The signed original informed consent documents for each participant and originals of all study documentation (e.g., study product inventory forms, participant clinic records, original laboratory reports, guidebooks) will be retained by the PI for a minimum of 2 years after FDA approval or withdrawal of a New Drug Application (NDA). If an NDA is not submitted within 5 years of the last follow-up visit, the site may request permission in writing from CONRAD to destroy the records. No records may be destroyed without written permission from CONRAD.

1. Investigator Responsibilities
   1. Prior to Starting Study
      1. Signing of Investigator’s Agreement and Amendments

Prior to study start the PI is responsible for signing and dating the Investigator’s Agreement for this study protocol. The signed and dated original must be submitted to CONRAD, and a copy must be maintained by the PI at the site with the study files.

All protocol amendments must be signed and dated by the PI. The signed and dated original must be submitted to CONRAD, and a copy must be maintained by the PI at the site. Amendments must be approved by CONRAD and the IRB before implementation.

- - 1. Forms and Records

Prior to study activation, and as applicable, the following forms and records will be provided to CONRAD, and a copy maintained in site files:

- Statement of the Investigator (FDA Form 1572)
- Curriculum vitae for staff listed on FDA Form 1572:
  - The PI will provide CONRAD with a curriculum vitae for him/herself showing the education, training, and experience that qualifies him/her as an expert in the area of clinical investigation specific to the product under investigation and his/her affiliation with the site at which the study is being conducted.
  - Curricula vitae will also be provided for study staff listed on the 1572 showing the education, training, and experience that qualifies them for their role in the study, and their affiliation with the study site.
- Financial Disclosure Statement (completed by each staff member listed on FDA Form 1572). Note that this form will also need to be provided for each staff person at the end of the study and one year following completion of the study, as possible.
- IRB information consisting of:
  - Name, address, and chairperson of the IRB
  - Multiple Project Assurance (MPA) or Federal-Wide Assurance (FWA) number
  - List of IRB members (names may be withheld in accordance with IRB policy) with title, occupation, and affiliation for each member
  - Copy of IRB approval letter for protocol, consent form, advertisement, and other written materials provided to participants
  - Copy of IRB-approved consent form, advertisements, and other written materials provided to participants, as applicable
- Laboratory information including:
  - Name of laboratories to be used to process study specimens
  - Curriculum Vitae of laboratory director(s)
  - Current license(s) and/or laboratory certification (such as the Clinical Laboratory Improvement Act [CLIA] certification), with expiration date
  - Copy of normal values for tests done by each laboratory for this study
  1. During the Study: Forms and Records

The following forms and records will be maintained at the study site, including but not limited to:

- Investigator’s Brochure or equivalent
- Subject Status Log(s)
- Subject Identification Code List
- Delegation of Responsibilities Log
- Site Visit Log
- Study Supplies Accountability Logs and packing slips
- Protocol Violation Log
- Source documents
- Signed and dated informed consent forms
- CRFs
- IRB documents:
  - Submission and approval letters for protocol and any protocol amendments
  - Submission and approval letters for original and any revised consent forms and any other written material provided to participants
  - Annual submission and approval letters
  - Other IRB correspondence
- Updates on curricula vitae and laboratory information
- General correspondence

Copies of all correspondence between the site and its IRB should be sent to CONRAD.

The PI is responsible for obtaining any updates to these documents, including certification renewals, and sending them to CONRAD in a timely fashion.

- 1. During the Study: Progress Reports

During the study, a monthly report will be sent to CONRAD and will include:

- Participant status
- Protocol violations
- AEs that are serious, urogenital, and/or related to product use

Annual progress reports and a final report will be submitted by the site to the IRB or IEC according to local regulations and guidelines.

1. References
2. Kott A. Rates of Unintended Pregnancy Remain High In Developing Regions. International Perspectives on Sexual and Reproductive Health. 2011;37(1).
3. Sedgh G, Singh S, Hussain R. Intended and Unintended Pregnancies Worldwide in 2012 and Recent Trends. *Studies in family planning*. 2014;45(3):301-314. doi:10.1111/j.1728-4465.2014.00393.x.
4. Singh S. Abortion Worldwide: A Decade of Uneven Progress. In: Wulf D, Hussain R, Bankole A, Sedgh G, eds: Guttmacher Institute; 2009:1-64.
5. UNAIDS. AIDS Epidemic Update, May 2016.
6. Abdool Karim Q, Abdool Karim SS, Frohlich JA, et al. Effectiveness and safety of tenofovir gel, an antiretroviral microbicide, for the prevention of HIV infection in women. Science. 2010;329(5996):1168-1174.
7. Burton FG, Skiens WE, Duncan GW. Low-level, progestogen-releasing vaginal contraceptive devices. Contraception. 1979;19(5):507-516.
8. Koetsawang S, Ji G, Krishna U, et al. Microdose intravaginal levonorgestrel contraception: a multicentre clinical trial. I. Contraceptive efficacy and side effects. World Health Organization. Task Force on Long-Acting Systemic Agents for Fertility Regulation. Contraception. 1990;41(2):105-124.
9. Sahota J, Barnes PM, Mansfield E, Bradley JL, Kirkman RJ. Initial UK experience of the levonorgestrel-releasing contraceptive intravaginal ring. Adv Contracept. 1999;15(4):313-324.
10. A randomized, double-blind study of six combined oral contraceptives. Contraception. 1982;25(3):231-241.
11. Sheth A, Jain U, Sharma S, et al. A randomized, double-blind study of two combined and two progestogen-only oral contraceptives. Contraception. 1982;25(3):243-252.
12. Thurman A, Schwartz J, Brache V, et al. Pharmacokinetics/Pharmacodynamics of Tenofovir and Tenofovir Plus Levonorgestrel Vaginal Rings in Women HIV Research for Prevention 2016: AIDS Vaccine, Microbicide and ARV-based Prevention Science (HIVR4P), Chicago, USA, 17 – 21 October 2016.
13. Marrazzo JM, Ramjee G, Richardson BA, Gomez K, Mgodi N, Nair G, et al. Tenofovir-based preexposure prophylaxis for HIV infection among African women. N Engl J Med. 2015 Feb 5;372(6):509-18. PubMed PMID: 25651245. Pubmed Central PMCID: 4341965

1. Drugs such as acetaminophen that can affect liver function and drugs that affect renal function may affect metabolism and should not be used on a daily basis during the study. [↑](#footnote-ref-1)
2. HSV-2 results are for analysis only. [↑](#footnote-ref-2)
3. Copan swabs will be collected at Visit 13 and Visit 22 and stored for possible analysis to assess for microflora during the study. [↑](#footnote-ref-3)
4. Drugs such as acetaminophen that can affect liver function and drugs that affect renal function may affect metabolism and should not be used on a daily basis during the study. [↑](#footnote-ref-4)
