# Supplementary material for: Randomized, placebo controlled phase I trial of the safety, pharmacokinetics, pharmacodynamics and acceptability of a 90 day tenofovir plus levonorgestrel vaginal ring used continuously or cyclically in women: The CONRAD 138 study
Source: PLoS One. 2022 Oct 10;17(10):e0275794. doi: 10.1371/journal.pone.0275794 (PMC9550080; doi:10.1371/journal.pone.0275794)
Supplement: Attachment — Submitted filename: Response to Reviewers THURMAN 20 APR 2022.docx [file pone.0275794.s009.docx]

Andrea Thurman, MD

Professor of Obstetrics and Gynecology

Eastern Virginia Medical School/CONRAD

601 Colley Ave, Norfolk, VA 23507

Email: [thurmaar@evms.edu](mailto:thurmaar@evms.edu), Phone: 210-380-5241

19 APR 2022

**Dear Dr. Royle, Editorial Team Members and Reviewers,**

Thank you for your thoughtful review of our manuscript, PONE-D-21-23955, “Randomized, Placebo Controlled Phase I Trial of Safety, Pharmacokinetics, Pharmacodynamics and Acceptability of a 90 day Tenofovir Plus Levonorgestrel Vaginal Ring in Women”. We have addressed all of the reviewer’s comments and recommended edits below. We point to the page and line number(s) of the changes in a revised, tracked version of the manuscript. We hope that these changes make our manuscript acceptable for publication.

Our amended funding statement is as follows: This study and the clinical development of the TFV/LNG ring were supported by the United States Agency for International Development (USAID) with funds from The U.S. President's Emergency Plan for AIDS Relief (PEPFAR) under Cooperative Agreements (AID-OAA-A-10-00068, AID-OAA-A-14-00010, and AID-OAA-A-14-00011). The Endocrine Technologies Core (ETC) at the Oregon National Primate Research Center (ONPRC) is supported by NIH Grant P51 OD011092 awarded to ONPRC. Tenofovir active pharmaceutical ingredient was donated by Gilead Sciences (Foster City, CA, USA).The contents are the sole responsibility of the authors and do not necessarily reflect the views of their institutions, PEPFAR, USAID or the United States Government.  The funders had no role in study design, data collection and analysis, decision to publish, or preparation of the manuscript. No author reports any conflict of interest or any competing interests which interfered with the conduct of this study or interpretation of results.

Please include the following items when submitting your revised manuscript:

Journal requirements:

When submitting your revision, we need you to address these additional requirements.

1. Please ensure that your manuscript meets PLOS ONE's style requirements, including those for file naming. The PLOS ONE style templates can be found at

[https://journals.plos.org/plosone/s/file?id=wjVg/PLOSOne_formatting_sample_main_body.pdf](https://urldefense.com/v3/__https:/journals.plos.org/plosone/s/file?id=wjVg*PLOSOne_formatting_sample_main_body.pdf__;Lw!!Aeua5Q!CrapZRUX_LwmqROn7e2Caa9Etap5TN6B_aArvtWuaNBTF5wUftCjdaVK_ECsnBoN$) and

[https://journals.plos.org/plosone/s/file?id=ba62/PLOSOne_formatting_sample_title_authors_affiliations.pdf](https://urldefense.com/v3/__https:/journals.plos.org/plosone/s/file?id=ba62*PLOSOne_formatting_sample_title_authors_affiliations.pdf__;Lw!!Aeua5Q!CrapZRUX_LwmqROn7e2Caa9Etap5TN6B_aArvtWuaNBTF5wUftCjdaVK_MtinfQD$).

RESPONSE: We confirm that the manuscript is formatted per PLOS One’s style requirements. Headings are 18 font, 2^nd^ level headings are 16 font, 3^rd^ level headings are 14 font. Text is double-spaced at 12 font. The references are cited using Plos style in EndNote. Figures and Tables and Supplemental materials are labeled as per the journal style format.

2. Thank you for stating the following in the Funding Section of your manuscript:

“This study and the clinical development of the TFV/LNG ring were supported by the United States Agency for International Development (USAID) with funds from The U.S. President's Emergency Plan for AIDS Relief (PEPFAR) under Cooperative Agreements (AID-OAA-A-10-00068, AID-OAA-A-14-00010, and AID-OAA-A-14-00011). The Endocrine Technologies Core (ETC) at the Oregon National Primate Research Center (ONPRC) is supported by NIH Grant P51 OD011092 awarded to ONPRC. The contents are the sole responsibility of the authors and do not necessarily reflect the views of their institutions, PEPFAR, USAID or the United States Government.  The funders had no role in study design, data collection and analysis, decision to publish, or preparation of the manuscript.  No author reports any conflict of interest or any competing interests which interfered with the conduct of this study or interpretation of results.”

We note that you have provided funding information that is not currently declared in your Funding Statement. However, funding information should not appear in the Acknowledgments section or other areas of your manuscript. We will only publish funding information present in the Funding Statement section of the online submission form.

RESPONSE: We have amended funding information and placed it under the Funding Statement section.

Please remove any funding-related text from the manuscript and let us know how you would like to update your Funding Statement. Currently, your Funding Statement reads as follows:

“This study and the clinical development of the TFV/LNG ring were supported by the United States Agency for International Development (USAID) with funds from The U.S. President's Emergency Plan for AIDS Relief (PEPFAR) under Cooperative Agreements (AID-OAA-A-10-00068, AID-OAA-A-14-00010, and AID-OAA-A-14-00011). The Endocrine Technologies Core (ETC) at the Oregon National Primate Research Center (ONPRC) is supported by NIH Grant P51 OD011092 awarded to ONPRC. The contents are the sole responsibility of the authors and do not necessarily reflect the views of their institutions, PEPFAR, USAID or the United States Government.  The funders had no role in study design, data collection and analysis, decision to publish, or preparation of the manuscript.  No author reports any conflict of interest or any competing interests which interfered with the conduct of this study or interpretation of results.”

RESPONSE: There are no funding related statements in the text of the manuscript. All funding related statements are now in the funding section.

Please include your amended statements within your cover letter; we will change the online submission form on your behalf.

RESPONSE: This amended text is included in the body of the cover letter.

3. We note that you have indicated that data from this study are available upon request. PLOS only allows data to be available upon request if there are legal or ethical restrictions on sharing data publicly. For more information on unacceptable data access restrictions, please see [http://journals.plos.org/plosone/s/data-availability#loc-unacceptable-data-access-restrictions](https://urldefense.com/v3/__http:/journals.plos.org/plosone/s/data-availability*loc-unacceptable-data-access-restrictions__;Iw!!Aeua5Q!CrapZRUX_LwmqROn7e2Caa9Etap5TN6B_aArvtWuaNBTF5wUftCjdaVK_MMS_vHV$).

In your revised cover letter, please address the following prompts:

a) If there are ethical or legal restrictions on sharing a de-identified data set, please explain them in detail (e.g., data contain potentially sensitive information, data are owned by a third-party organization, etc.) and who has imposed them (e.g., an ethics committee). Please also provide contact information for a data access committee, ethics committee, or other institutional body to which data requests may be sent.

RESPONSE: There are no ethical or legal restrictions on de-identified study endpoint data. The rest is not applicable.

b) If there are no restrictions, please upload the minimal anonymized data set necessary to replicate your study findings as either Supporting Information files or to a stable, public repository and provide us with the relevant URLs, DOIs, or accession numbers. For a list of acceptable repositories, please see [http://journals.plos.org/plosone/s/data-availability#loc-recommended-repositories](https://urldefense.com/v3/__http:/journals.plos.org/plosone/s/data-availability*loc-recommended-repositories__;Iw!!Aeua5Q!CrapZRUX_LwmqROn7e2Caa9Etap5TN6B_aArvtWuaNBTF5wUftCjdaVK_P4CkBTk$).

We will update your Data Availability statement on your behalf to reflect the information you provide.

RESPONSE: We will deposit de-identified data with PLOS One as part of the manuscript submission.

Reviewers' comments:

Reviewer's Responses to Questions

**Comments to the Author**

1. Is the manuscript technically sound, and do the data support the conclusions?

The manuscript must describe a technically sound piece of scientific research with data that supports the conclusions. Experiments must have been conducted rigorously, with appropriate controls, replication, and sample sizes. The conclusions must be drawn appropriately based on the data presented.

Reviewer #1: Yes

Reviewer #2: Yes

RESPONSE: Thank you

2. Has the statistical analysis been performed appropriately and rigorously?

Reviewer #1: Yes

Reviewer #2: No

RESPONSE: We made the edits from reviewer #2 to the statistical methods below

3. Have the authors made all data underlying the findings in their manuscript fully available?

The [PLOS Data policy](https://urldefense.com/v3/__http:/www.plosone.org/static/policies.action*sharing__;Iw!!Aeua5Q!CrapZRUX_LwmqROn7e2Caa9Etap5TN6B_aArvtWuaNBTF5wUftCjdaVK_MmMzzjj$) requires authors to make all data underlying the findings described in their manuscript fully available without restriction, with rare exception (please refer to the Data Availability Statement in the manuscript PDF file). The data should be provided as part of the manuscript or its supporting information, or deposited to a public repository. For example, in addition to summary statistics, the data points behind means, medians and variance measures should be available. If there are restrictions on publicly sharing data—e.g. participant privacy or use of data from a third party—those must be specified.

Reviewer #1: Yes

Reviewer #2: Yes

RESPONSE: Thank you – we will also deposit the anonymized data as per requested above by the journal requirements.

 4. Is the manuscript presented in an intelligible fashion and written in standard English?

PLOS ONE does not copyedit accepted manuscripts, so the language in submitted articles must be clear, correct, and unambiguous. Any typographical or grammatical errors should be corrected at revision, so please note any specific errors here.

Reviewer #1: Yes

Reviewer #2: No

RESPONSE: We made the edits and clarifications requested by reviewer #2 below.

5. Review Comments to the Author

Please use the space provided to explain your answers to the questions above. You may also include additional comments for the author, including concerns about dual publication, research ethics, or publication ethics. (Please upload your review as an attachment if it exceeds 20,000 characters)

**Reviewer #1: A 4-arm randomized, placebo-controlled phase I safety trial was conducted which reported pharmacokinetics, pharmacodynamics and acceptability. All IVRs were safe and no serious adverse events were encountered.**

**Minor revisions:**
1- Page 10: Indicate if adverse events were collected according to a standardized method, e.g. by CTCAE.

RESPONSE: We clarified on page 12 of the revised tracked manuscript that per protocol, adverse events were graded for severity, using the DAIDS tables for grading the severity of adverse events (http://rsc.tech-res.com/clinical-research-sites/safety-reporting/daids-grading-tables) and relationship to study product or study procedures (reported as related versus not related). Adverse events were coded with the appropriate MDR code for the clinical study report and for data presentation.

2- Define the abbreviations SD and STD at their first occurrence.

RESPONSE: “Standard deviation” is defined at the first occurrence, Table 1, and is uniformly abbreviated as SD now in Tables 1, 3, 4, 6, 7 and 8.

3- Page 25, line 4: State the statistical method from which the paired p-values were estimated.

RESPONSE: We added in the Sample size and statistical analyses section (pages 18 – 20) that the Wilcoxon signed rank sum test was used to compare paired changes from baseline, pre-insertion safety endpoints to the end of treatment values (e.g. density of tissue lymphocytes and concentrations of secreted soluble proteins) as these data are not normally distributed. Similarly, the Kruskall-Wallis test was used to compare safety endpoints among the 4 independent treatment/dosing groups at the end of treatment. We put this clarification in the Methods section because it applies to the statistical analyses presented for both immune cells and cytokines.

4- Provide a comprehensive statistical analysis section. State all statistical methods used, including chi square tests, and Spearman correlations.

RESPONSE: We have added on pages 19 and 20 all statistical tests used to compare safety, PK and PD data. We also added the correlation methods used to correlate PK and PD variables.

5- To improve transparency, on each table indicate the statistical testing method(s) used to estimate the p-values.

RESPONSE: The statistical test for calculating the p values has been added to the Table legend for Tables 2, 3, 4, 5, and 7. Additional information about the statistical analyses has been added to the methods section, pages 19 and 20 in the revised tracked manuscript.

**Reviewer #2: MAJOR COMMENTS**

As general comment, I highly recommend a complete re-writing of the manuscript to obtain a more formal, better-structured text. As an example, try to keep the same scheme for describing methods in all the sub-sections of this section (as a guidance: which type of sample, when in the study, what to analyze in it). Finally, try to avoid so many abbreviations throughout the text (and always in titles), mainly the non-standard abbreviations (CM, CV, CVF, CVL….)

RESPONSE: We have edited the manuscript to a more formal writing style. In the past, many reviewers have asked that we either use a first person active language or that we use the third person past tense style. We have removed abbreviations from the section headings. We do believe that CV, CVF and CVL are standard abbreviations for this field and type of Phase I study and would therefore like to keep these abbreviations. In addition they are used frequently throughout the text. We have now spelled out cervical mucus. We have formatted the methods text as requested and agree that presenting sample type, when in the study and then specified in the statistical section the tests used to compare each endpoint. Due to the length of the manuscript, we added additional methods details in a supplemental methods section.

Statistical methods must be reviewed. The use of parametrical test with low sample size and disbalanced groups must be very well justified…if possible!

RESPONSE: As also requested by reviewer #1, we have added additional detail to the statistical methods section, pages 18 – 20, specifying which tests were used for each endpoint comparison. We added on page 19 that all data sets were examined for normality using the PROC UNIVARIATE procedure in SAS and examining the histogram, Q plot and the Shapiro Wilk statistic. All continuous variables were not normally distributed and therefore appropriate non-parametric statistical testing was performed. We also note on page 19 that the sample size for this phase 1 study, like most phase 1 studies and particularly those done previously in this field or specifically on this MPT product, was primarily based on the size of similar studies MPT IVRs [1]. For statistical comparisons, we note on page 19, lines 7 – 11, that the primary objective of the study, safety, was compared using exact statistical methods whenever possible for the reporting of TEAEs between treatment groups. For transparency, we have also added the Fisher exact test p values for proportion of participants reporting various TEAE categories to Table 2. We acknowledged on page 19, lines 9 – 11, that other statistical comparisons must be interpreted with caution for this phase 1 study due to the limited sample size, particularly for exploratory modeled PD objectives.

Finally, try to follow the style of your previous paper (PLOS one 2018), with some exceptions below recommended.

RESPONSE: As requested we have changed the methods section of this manuscript to reflect the style of our previous PLOS One ring manuscript (2018), explaining at what visits samples were collected, the sample collection specifics and then the sample analysis details. Because this is extensive information, we have also included a supplemental methods section to reduce main manuscript length.

**MINOR COMMENTS**
Pg.5, Ln.12-23: The difference between the CONRAD 128 (15 days use) and this study (up to 90 days) is not apparent enough after a quick reading. Then, the wording should be improved. As a suggestion, split it in two paragraphs: The first one to summarize the CONRAD 128, including some results from this study to make more obvious that this study was completed. The second paragraph, dedicated to the current paper as a subsequent study from the former, finalizing with the objectives.

RESPONSE: We agree that this is a very important distinction to make between the initial first-in-woman study of 15 days of continuous use (CONRAD 128) [1] and this first study of the TFV/LNG IVR for the full 90 day duration. We have added these details, as suggested in two paragraphs, to the introduction on pages 5 and 6 of the revised tracked manuscript.

Pg.6. A scheme of activities and short description of visits is mandatory at the beginning of Methods sections. Throughout the section, refer to this figure when necessary and avoid the continuous quoting of “…prior to insertion….”, “….at the end of….”, or similar sentences.

RESPONSE: We have moved the sentence “The study visits and procedures are summarized in Supplementary Table 1” to the beginning of the methods section, clinical study section, page 7 lines 1 – 2. Given that this study had 32 visits, we believe the best method to orient the reader to the multiple visits and endpoints is a standard schedule of evaluations table, with which most clinical study researchers are familiar. Throughout the manuscript, when we refer to visits, we have now cited Supplemental Table 1. Plos One format style has Table 1 describe the study population and therefore we chose, as we have done in our previous Plos One manuscripts, to outline the study visits in Supplemental Table 1.

Pg.6,Ln.14. The inclusion/exclusion criteria must be summarized.

RESPONSE: We have added additional inclusion and exclusion criteria to the main I/E criteria noted in the methods section, page 7.

Pg.6,Ln.20-22: The 4 arms are explained (and better explained) in Pg.8,Ln.8-11.

RESPONSE: We copied the randomization text on page 8 to better explain the study groups (now on pages 7 and 8) in the overall methods section. This allowed us to delete some of the redundant detail in the randomization section on page 10 of the revised tracked manuscript.

Pg.7, Ln.3: It is not clear if the ring was inserted at a specific time of the cycle (e.g., Days 1 to 5 of menstruation) or was inserted at any moment of the cycle, regardless the menstrual status.

RESPONSE: We have added in the methods section that IVR insertion at visit 4 was performed in the menstrual/follicular phase on day 6 ± 1 day of the menstrual cycle on page 8, lines 6 – 9.

Pg.7, Ln.4: Please explain the different extraction regimen “based on the randomization assignment”.

RESPONSE: We clarified, on page 8, lines 10 – 14, that the PK sampling time at visit 5 was based on a randomization where participants had blood and CV tissue for PK obtained at 24 OR 48 OR 72 hours after IVR insertion. We also clarified that this randomization was done because participants could not have CV tissue obtained at all three time points for safety reasons (page 8, lines 12 – 14). When CV tissue biopsies are taken, we place Monsel’s solution on the biopsy site to prevent bleeding. We don’t want the participants to have anything in the vagina for 5 days after a biopsy, including doing a repeat speculum exam to obtain more biopsies. This could result in bleeding from the biopsy site.

Pg.7, Ln5: Please explain how cervicovaginal (CV) tissue is used for PK purposes. In addition, abbreviation “CV” must be clarified since this is the first time of use (cervicovaginal).

RESPONSE: We now define CV at the first presentation on page 8. We also clarified that the tissue biopsies for PK were two vaginal tissue biopsies (page 15, line 7). In the methods section, we detail how the Marzinke central PK lab at Johns Hopkins processed the vaginal tissue for both tenofovir and tenofovir-diphosphate concentrations as part of the pharmacokinetic assessment of the rings. PK parameters are now presented in supplemental Table 3. We also detail in the discussion section (pages 53 – 55) how tissue concentrations of TFV in our current study compare to past studies in women using TFV topical vaginal gel [2-4].

Pg.7, Ln14: Please clarify when did you collect CM ….end of the month 1, 2 or 3 visit…

RESPONSE: We have added to the description of the timing of cervical mucus checks, in the methods section on pages 8 – 9 that “To detect ovulation and time cervical mucus assessments, participants returned approximately twice weekly after IVR initiation, to have serum estradiol (E2) and P4 measured. We performed a cervical mucus check for Insler score [7] and sperm penetration assay (modified slide test) [8-12] at the next regularly scheduled visit, usually within 48 hours, if the serum E2 was between 75 – 150 pg/mL. If the serum E2 was over 150 pg/mL, indicating imminent follicular development, we brought the participant in for a cervical mucus check within 24 hours. If the serum E2 did not reach 75 pg/mL before the end of each month (i.e. days 28, 59 and 90), we collected cervical mucus at the end of the month 1 (V13), month 2 (V22) or month 3 (V31) visits (S1 Table)”.

Pg.9,Ln11: …designed to release approximately 20 u/day. See Ln.6.

RESPONSE: Since the release rate was defined on page 11, line 11 as (20 ug/day), we deleted the redundant phrase on page 11, line 16.

Pg.10,Ln 22: As stated above, please: Type of sample > when > what to do.

RESPONSE: We have added a standard structure to the methods section, which explains type of sample, when collected and what methods were used to process the samples. We also added reference to the Supplemental Table 1, to direct the reader to the schedule of evaluations.

Pg.11,Ln 3-4: Since no further comments will be included, please consider to delete this sub-section.

RESPONSE: We agree and have deleted this sub-section on page 13 of the revised tracked manuscript.

Pg.11, Ln 7-13: This is an example of re-witting and simplification, as suggested above: At visits 4 and 29, a CV fluid lavage was collected in 4ml normal saline after speculum insertion and lavage of the cervical fornices and vaginal walls, avoiding spraying directly into the cervical os. The concentration of soluble proteins in the supernatant was then measured by multiplex electrochemiluminescence assay and ELISA, using procedures established under accreditation by the College of American Pathologists [6].

RESPONSE: Thank you for the alternative text. We have inserted this text on page 14, lines 3 - 7 based on your recommendation.

Pg.11,Ln21: ….a subset of XX participants….. Though again, since no further comments will be included, please consider to delete this sub-section.

RESPONSE: We agree and have deleted the text on page 14 regarding the individual qualitative interviews as these data will be reported separately.

Pg.12, Ln4: Strictly speaking, “Pharmacokinetics” (PK) is currently defined as the study of the time course of drug absorption, distribution, metabolism, and excretion. The measurement of drug concentration in tissues or the quantification of binding protein cannot be considered as PK. The sub-sections must be re-written following the previous recommendation and the times of quantification must be included on the text (Supplemental is not appropriate for this purpose).

RESPONSE: We agree with the Reviewer that we have not done a full ADME study with the rings, but we have collected enough drug concentration data in different compartments to present some key PK parameters, which illustrate how the rings perform and may achieve the desired effects. We have edited the methods section accordingly and added supplemental Table 3 with PK parameters. Because multiple samples were obtained from multiple matrices, we refer the reader to the S1 Table for each time point, to reduce word length. In addition, to make sure that the PK methods are defined in detail, while respecting the journal’s word limits, we have added ample information to the supplemental methods section on the calculation of PK parameters.

Pg.13,Ln.8: This sub-section is described in much more detail than the other sub-sections. The style must be homogenized throughout the manuscript. Since there is a Supplemental, this sub-section must be reduced in agreement with the previous.

RESPONSE: To assess anti-HSV2 activity (now pages 16 and 17 of the revised tracked manuscript), two methods were used. The Vero cell method has been previously reported ,and therefore we summarized it and added references (page 16). The method using HEC1A epithelial cells is a new method, developed and refined by our group and not previously reported; therefore, we chose to describe this method more comprehensively to enable others to reproduce.

Pg.15,Ln.3: Contraceptive efficacy can only be measured by the follow-up for a long enough time of a cohort of sexually active women (please, see Pearl Index). In this sub-section the PD of LNG is addressed, as well described in its title.

RESPONSE: We agree and have edited this section header on page 18 to clarify that this is LNG PD assessments as *surrogates of contraceptive effect*. In line with the type of study (Phase I), we have assessed LNG PD effects (e.g. anovulation, thickened cervical mucus) as surrogates of potential contraceptive activity.

Pg.15, Ln.10: Already described in Pg.7,Ln.21-23. Improve this content in pg.7 and delete this subsection in pg. 15.

RESPONSE: We agree with the recommendation and have added detail and referenced our methods for the assessment of objective adherence biomarkers on page 9 as part of the clinical study methods. This allowed us to delete the redundant text on page 18 of the tracked manuscript.

Pg.15, Ln.16: Please confirm that the study was authorized by IRB and without a formal calculation of sample size.

RESPONSE: We confirm the text in the methods section, page 7 “The study was approved by the Advarra Institutional Review Board (IRB) (#Pro00022358) and Comisiòn Nacional de Bioetica (#030-2017), respectively, and registered with ClinicalTrials.gov (#NCT03279120)”. The statistical summary in the approved protocol (also submitted to Plos One) states in the study synopsis and the statistical analyses summary sections the same verbage as reported in the manuscript, on page 19. Our group does several first in woman and phase I studies and it is the norm for these type of studies to be primarily descriptive in nature. We also note, and have added this to the manuscript, page 19, that the primary objective safety, was measured using TEAEs. For this endpoint, we used the Fisher exact test for comparisons, which is not dependent on sample size.

Pg.16,Ln.1: A real PK analysis must include a description of the concentration/time curve….

RESPONSE: We agree and have added the calculation of standard PK parameters to the supplemental methods section. We have also included the calculated PK parameter data for TFV in vaginal fluid and vaginal tissue, TFV-DP in vaginal tissue, TFV in plasma and LNG and SHBG in serum as Supplemental Table 3. Furthermore, following the Reviewer’s suggestion below, we have changed the figure to plot the data as concentration/time curves. The PK parameters are compared to established benchmarks for LNG contraceptive concentrations [13] and vaginal fluid TFV [14, 15] and vaginal tissue TFV-DP concentrations in regard to HIV antiviral activity [5, 6], which were previously established from either human cross sectional studies (LNG contraceptive PK [13] and vaginal fluid TFV [14, 15]), or SHIV infection experiments in non-human primates days [5, 6]. These references and the discussion of all these PK benchmarks are included in the discussion section pages 52 - 55. Because a Supplemental Table 3 was added, this makes the rectal and vaginal anti-HSV2 data now Supplemental Table 4.

Pg.16,Ln.11: “For select PD…” it is not acceptable, must be for all, unless a good explanation of selection criteria is provided.

RESPONSE: We apologize for this error. We have specified that for comparison of the anti-HIV inhibition and p24 antigen product between the 4 treatment/dosing groups an ANOVA model was used, page 20.

Pg.16,Ln6-20: The statistical methods must be reviewed. The proposal of parametrical tests sound inadequate for such a low sample size and disbalanced between groups.

RESPONSE: No parametric test has been used to statistically compare data sets following non-Gaussian distributions. As pointed out above, we have added the SAS code (PROC univariate) that we used to determine the normality of each endpoint data set on page 19. We specifically note on page 19 that all continuous variables were not normally distributed and therefore appropriate non-parametric statistical testing was performed. We now also note the statistical test used to compare data as footnotes in all tables. We note in the statistical methods section that the primary objective of the study, safety, was compared using exact statistical methods for the reporting of TEAEs between treatment groups on page 19. We acknowledge in the statistical analyses section that other statistical comparisons must be interpreted with caution for this phase 1 study due to the limited sample size, particularly for exploratory modeled PD objectives.

Pg.16.Ln.12: The ANCOVA model must be appropriately described. But prior, review the appropriateness of this statistical method.

RESPONSE: You are correct that the ANCOVA model, which we have used in the past to categorize “infected” versus “not infected” categories of the p24 antigen production PD model was not used or reported in this manuscript and therefore we have removed this model from the statistical methods section on page 20.

Pg.16.Ln.17: It is not clear if paired t-test is used for the comparison of more than 2 times, which is inappropriate.

RESPONSE: We have clarified in Table 6 that paired changes from baseline to month 1 and paired changes from baseline to month 3 were calculated using the non-parametric Wilcoxon signed rank sum test. We recognize that multiple comparisons could have also been performed, but with only two time points from baseline, paired non-parametric analyses are also accurate. We added additional text in the methods section to clarify this statistical process (page 20, lines 1 – 7) for the HIV inhibition of vaginal fluids endpoint.

Pg.16.Ln.21: No description of the safety analysis is missing.

RESPONSE: As is typical for phase 1 studies, we clarified in the methods section (Safety Assessments sub-section) that the primary safety measure was treatment emergent adverse events (TEAEs). We added, based on reviewer #1’s comments how TEAEs were graded (NIH/NIAID/DAIDS criteria) and how relationship to study product or study procedures was determined on page 12, lines 8 - 10. The other sub-clinical safety assessments outlined in the methods section include the assessment of the density and phenotype of immune cells in ectocervical tissue and changes from baseline in the concentration of secreted soluble proteins from the CV mucosa.

Pg.17,Ln.11: Please clarify: If the subject was allocated in one arm, the subject was randomized and therefore enrolled (by definition). Do you mean that she was not included on the analysis? Was the analysis ITT or PP or both? (Not specified on the Statistitics sub-section).

RESPONSE: We have added a description of the randomized, treated and evaluable population to the statistical analysis section on page 19, lines 12 - 18

Pg.17,Ln.13: Demographic characteristics of participants.

RESPONSE: As per Plos One journal style, the title of the figure (in this case Figure 1) is listed after the paragraph in which the figure is referenced, on page 22, line 8. The title of Figure 1 is Disposition of participants. The title of Table 1 is Demographic and baseline characteristics treated population. Per Plos One formatting requirements, titles of tables are listed below the table on page 24.

Pg.20,Ln.1-2: Please delete, not necessary.

RESPONSE: We have deleted this text on page 25, lines 1 and 2.

Pg.20,Ln.5-15: Please, use a table to show the exposed population by arm.

RESPONSE: We agree that this is a more straightforward way to display exposure data and have included this text as data in the main safety table, Table 2.

Pg.20,Ln.16: Please, include a sentence that all the AEs recorded were considered as TEAE (if so!).

RESPONSE: We have included on page 12, lines 6 and 7 of the methods section, page 11, that TEAEs were monitored at every visit, starting with the enrollment visit and therefore all adverse events were considered to be TEAEs, not medical history.

20.19: In accordance with this sentence, the incidence of TEAE was lower in interrupted treatment than in placebo.

RESPONSE: We have clarified, using the Fisher exact statistical comparison of the proportion of participants in each of the 4 treatment/dosing groups reporting various levels of TEAEs and the p values are now displayed in Table 2. This clarifies that while there were trends, although none were statistically significant (page 25, lines 18 – 20).

20.1-….:Please, use the classification by ICH-E2A to show the safety results, including the classification of AE by severity, causality and seriousness. Additionally, expectedness. And this classification should be explained in Methods.

RESPONSE: We have outlined additional detail on the classification of severity of TEAEs (mild, moderate, severe, potentially life threatening and death) and gave the link for the DAIDS tables (<http://rsc.tech-res.com/clinical-research-sites/safety-reporting/daids-grading-tables>) which were used to grade the severity of TEAEs in this study on page 12, lines 6 - 20. We added definitions used for classifying a TEAE as suspected versus unexpected and serious (page 12, lines 12 – 20). We have added information to the results section that there were no SAEs, no unexpected TEAEs or deaths (page 25, line 16).

Page 23.13: Delete “(all…0.05)”

RESPONSE: This text has been deleted on page 30, line 5.

23.15: See 23.13.

RESPONSE: The text of all p values > 0.07 has been deleted on page 30, line 7.

23.16: of ….N…. samples. And the same in next pages, when p is > 0.05. The statistical significance level at p<0.05 is already established in Methods/Statistical methods (16.20).

RESPONSE: We added the detail that there were 12 ectocervical biopsies which could be split for standard formalin fixation versus cryopreservation (done at the EVMS site) for this analyses (page 30, line 8). We also changed the text to say that there were no statistically significant differences between the CD4 and CCR5 lymphocyte densities between TFV/LNG and placebo IVR groups (page 30, lines 8 and 9). However these conclusions, as we noted, are limited by the small sample size (displayed in Supplemental Table 2).

Page 24. To simplify this one and the following tables, you can choose between either Mean&SD (if data follow a Normal distribution) or alternatively, Median&Min-Max (if not normal).

RESPONSE: We agree that normally distributed data is displayed by Mean and SD and non-parametric data is best displayed by median with either IQR or min max. However, we would like to include both mean/SD and median to give the reader a better understanding of the data.

Page 27.1-9: Considering the limited sample size in the placebo group, the use of percentages is not appropriate. The description in absolute values (1/5… One of the 5 …. Etc.) is more adequate and simplifies the paragraph.

RESPONSE: We clarified in the methods section, that the proportions noted for adherence are the percent of expected days of use (page 9, lines 10 – 17). The percentages are not the percent of participants with perfect use. Specifically, based on self-report and observations of IVR use in the clinic, for the continuous arms, the total number of days with the IVR was determined as (the last day of IVR removal – the first day of IVR insertion + 1) – the total number of days of unintentional IVR removal. The expected number of days with the IVR for continuous use was 90 days. For the interrupted arms, the total number of days with the IVR was determined as (the last day of IVR removal – the first day of IVR insertion +1) – (the total number of days of scheduled and unintentional IVR removal). The expected number of days with the IVR for interrupted use was 84 days. We have added verbage to the results section (page 34, lines 2 and 3 and lines 7 and 8) to clarify that this is the mean proportion of days the IVR was used of the expected days of use. We also categorized the proportion of expected days used as 100%, ≥ 80% to < 100%, and < 80%.

Page 27.12-17: This comments correspond to Discussion.

RESPONSE: We agree and took out the comments from the results section (objective biomarkers) (page 34, lines 13 – 19) and placed these in to the discussion section (page 51, lines 14 – 23) regarding how these objective biomarker data compare to our previous validation study study [16].

28.10: Considering the high number of tables, avoid Table 5 and include only relevant results in the text.

RESPONSE: Given that bleeding abnormalities are the main reason that women discontinue effective progestin only based contraceptives [17], we would like to keep Table 5. It contains data that the field is looking for. We proposed a microdose of topical LNG (20ug/day) to reduce menstrual irregularities with this LNG MPT [18], and therefore, data on menstrual cycle impact, reported in this cohort over 3 months of use, represent unique and valuable information.

30.2: Results must be shown. A figure is recommended.

RESPONSE: We agree and have added the TFV in plasma PK data to Supplemental Table 3.

34. This table can be included on the text.

RESPONSE: We agree and have included the plasma TFV PK data in Supplemental Table 3

38.19-23. The statistical methods (ROC, Fisher, Spearman…) must be described only on the Methods section, not in Results.

RESPONSE: We agree and have moved the statistical methods used for the post hoc categorization of the anti-HSV2 data to the statistical methods section (page 21, lines 6 – 13).

41.4. The higher frequency of TEAE in interrupted than in continuous treatment (Pg.21,Ln.6) must be discussed at any point of this section.

RESPONSE: As stated above, we used the Fisher exact test to compare the proportion of participants in each study treatment (TFVLNG or placebo IVR) and each dosing regimen (continuous or interrupted) as this was the primary safety endpoint and we recognize that exact tests should be used when the sample size or cell sizes are low. Although there were some trends noted, there were no statistically significant differences in reports of various levels of TEAEs by participants randomized to the four cohorts studied. We have included the summary sentence: “The TFV/LNG IVR was safe and well tolerated. There were no significant differences in the proportion of participants randomized to one of the four treatment and dosing groups in their reports of various levels of TEAEs and the relatedness of these TEAEs to the study product or study procedures” as the top line assessment in the discussion section, pages 48 and 49.

41.10. The inclusion of a not sexually active participant should be justified or commented.

RESPONSE: We have added to the introduction that the initial first in woman study, CONRAD 128, did not allow sexual activity to limit exposure of a male partner to an IND product (page 6, lines 1 - 3). After proving safety in the first study, we allowed sexual activity in this 3 month use study because it was not feasible to limit participant’s sexual activity for this long. Furthermore topical tenofovir has been used previously as a topical gel by thousands of sexually active women in phase 2/3 efficacy trials [19-21]. However, as the 138 study was still a phase 1 study to assess safety, PK and model PD in women, we did not require participants to be heterosexually active as no endpoint in this study was related to a male partner, nor required timed intercourse and this study was not assessing sero-conversion.

42.2. How was resolved? With the removal of the ring? With additional treatment?

RESPONSE: We have added additional details on page 49, lines 9 – 10, to this participant’s finding and recovery. We removed her IVR and her epithelial disruption completely resolved within 10 days without additional treatment.

42.23. Avoid the direct relationship of bleeding irregularities and anemia. Menstrual irregularities can involve also amenorrhea, or irregular bleeding not necessarily excessive and hence not related with anemia. From other perspective, the potential reason for discontinuation is the irregular bleeding pattern, not the abnormal laboratory finding.

RESPONSE: We agree and have removed the verbage referring to serum hemoglobin and ferritin concentrations on page 51, lines 1 and 2.

44.3. Clarify that the plasma levels refers to the steady state. Not necessary to specify the method of quantification.

RESPONSE: That is correct, these are steady state concentrations of serum LNG in previous studies of the LNG implant (Norplant). We have added this clarification on page 52, lines 15 and 17.

44.8. The use of the same method of quantification does not guarantee that independent results can be directly compared.

RESPONSE: We completely agree. We have added additional detail on pages 52 and 53, to emphasize that the past contraceptive benchmarks for levonorgestrel were for systemic delivery (implants) and were established using RIA on page 52. Although we were able to use RIA in our first study, this method has been replaced with LC/MS-MS. We have added an additional reference [22] (reference 27 in the tracked revised manuscript), which also notes that RIA and LC/MS-MS quantification are not directly comparable.

Fig.2-3 The use of concentration/time CURVES is recommended (instead of bars). Please, taking in consideration that X-axis is time.

RESPONSE: We have changed the bar graphs showing mean (SD) in Figures 2 and 3 to concentration/time graphs. We have added that the X axis is time.

Again, we thank the reviewers for their thoughtful comments. We hope that the edits and clarifications added makes our manuscript acceptable for publication in PLOS One.

Sincerely,

Andrea Thurman

REFERENCES FOR REVIEWER RESPONSE LETTER:

1.	Thurman AR, Schwartz JL, Brache V, Clark MR, McCormick T, Chandra N, et al. Randomized, placebo controlled phase I trial of safety, pharmacokinetics, pharmacodynamics and acceptability of tenofovir and tenofovir plus levonorgestrel vaginal rings in women. PLoS One. 2018;13(6):e0199778. Epub 2018/06/29. doi: 10.1371/journal.pone.0199778. PubMed PMID: 29953547; PubMed Central PMCID: PMCPMC6023238.

2.	Schwartz JL, Rountree RW, Kashuba ADM, Brache A, Creinin M, Poindexter A, et al. A Multi-Compartment, Single and Multiple Dose Pharmacokinetic Study of the Vaginal Candidate Microbicide 1% Tenofovir Gel. PLoS One. 2011;6(10):e25974.

3.	Herold BC, Chen BA, Salata RA, Marzinke MA, Kelly CW, Dezzutti CS, et al. Impact of Sex on the Pharmacokinetics and Pharmacodynamics of 1% Tenofovir Gel. Clin Infect Dis. 2016;62(3):375-82. doi: 10.1093/cid/civ913. PubMed PMID: 26508513; PubMed Central PMCID: PMC4706638.

4.	Hendrix CW, Chen BA, Guddera V, Hoesley C, Justman J, Nakabiito C, et al. MTN-001: randomized pharmacokinetic cross-over study comparing tenofovir vaginal gel and oral tablets in vaginal tissue and other compartments. PLoS One. 2013;8(1):e55013. doi: 10.1371/journal.pone.0055013. PubMed PMID: 23383037; PubMed Central PMCID: PMC3559346.

5.	Parikh UM, Dobard C, Sharma S, Cong ME, Jia H, Martin A, et al. Complete protection from repeated vaginal simian-human immunodeficiency virus exposures in macaques by a topical gel containing tenofovir alone or with emtricitabine. J Virol. 2009;83(20):10358-65. Epub 2009/08/07. doi: JVI.01073-09 [pii]

10.1128/JVI.01073-09. PubMed PMID: 19656878; PubMed Central PMCID: PMC2753130.

6.	Dobard C, Sharma S, Martin A, Pau CP, Holder A, Kuklenyik Z, et al. Durable protection from vaginal simian-human immunodeficiency virus infection in macaques by tenofovir gel and its relationship to drug levels in tissue. J Virol. 2011;86(2):718-25. Epub 2011/11/11. doi: JVI.05842-11 [pii]

10.1128/JVI.05842-11. PubMed PMID: 22072766; PubMed Central PMCID: PMC3255839.

7.	Insler V, Melmed H, Eichenbrenner I, Serr DM, Lunenfeld B. The Cervical Score: A Simple Semiquantitative Method for Monitoring of the Menstrual Cycle. International Journal of Gynaecology and Obstetrics. 1972;10(6):223-7.

8.	Leader A, Wiseman D, Taylor PJ. The prediction of ovulation: a comparison of the basal body temperature graph, cervical mucus score, and real-time pelvic ultrasonography. Fertil Steril. 1985;43(3):385-8. PubMed PMID: 3884396.

9.	Nulsen J, Wheeler C, Ausmanas M, Blasco L. Cervical mucus changes in relationship to urinary luteinizing hormone. Fertil Steril. 1987;48(5):783-6. PubMed PMID: 3311823.

10.	Abidogun KA, Ojengbede OA, Fatukasi UI. Prediction and detection of ovulation: an evaluation of the cervical mucus score. Afr J Med Med Sci. 1993;22(1):65-9. PubMed PMID: 7839884.

11.	Organization WH. The World Health Organization Laboratory Manual for the Examination of Human Semen and Sperm-Cervical Mucus Interaction. Fourth ed. Cambridge, United Kingdom: Cambridge University Press; 1999.

12.	Pandya IJ, Mortimer D, Sawers RS. A standardized approach for evaluating the penetration of human spermatozoa into cervical mucus in vitro. Fertil Steril. 1986;45(3):357-65. PubMed PMID: 3949035.

13.	Cherala G, Edelman A, Dorflinger L, Stanczyk FZ. The elusive minimum threshold concentration of levonorgestrel for contraceptive efficacy. Contraception. 2016;94(2):104-8. doi: 10.1016/j.contraception.2016.03.010. PubMed PMID: 27000997.

14.	Kashuba AD, Gengiah TN, Werner L, Yang KH, White NR, Karim QA, et al. Genital Tenofovir Concentrations Correlate With Protection Against HIV Infection in the CAPRISA 004 Trial: Importance of Adherence for Microbicide Effectiveness. J Acquir Immune Defic Syndr. 2015;69(3):264-9. doi: 10.1097/QAI.0000000000000607. PubMed PMID: 26181703; PubMed Central PMCID: PMC4505741.

15.	Karim SS, Kashuba AD, Werner L, Karim QA. Drug concentrations after topical and oral antiretroviral pre-exposure prophylaxis: implications for HIV prevention in women. Lancet. 2011;378(9787):279-81. doi: 10.1016/S0140-6736(11)60878-7. PubMed PMID: 21763939; PubMed Central PMCID: PMC3652579.

16.	Jacot TA, Clark MR, Adedipe OE, Godbout S, Peele AG, Ju S, et al. Development and clinical assessment of new objective adherence markers for four microbicide delivery systems used in HIV prevention studies. Clin Transl Med. 2018;7(1):37. Epub 2018/11/08. doi: 10.1186/s40169-018-0213-6. PubMed PMID: 30402770; PubMed Central PMCID: PMCPMC6219998.

17.	Polis CB, Hussain R, Berry A. There might be blood: a scoping review on women's responses to contraceptive-induced menstrual bleeding changes. Reprod Health. 2018;15(1):114. Epub 2018/06/27. doi: 10.1186/s12978-018-0561-0. PubMed PMID: 29940996; PubMed Central PMCID: PMCPMC6020216.

18.	Xiao BL, Zhang XL, Feng DD. Pharmacokinetic and pharmacodynamic studies of vaginal rings releasing low-dose levonorgestrel. Contraception. 1985;32(5):455-71. Epub 1985/11/01. PubMed PMID: 3936678.

19.	Karim QA, Karim SSA, Frohlich JA, Grobler AC, Baxter C, Mansoor LE, et al. Effectiveness and Safety of Tenofovir Gel, an Antiretroviral Microbicide, for the Prevention of HIV Infection in Women. Science Express. 2010;10.1126(1193748):1.

20.	Delany-Moretlwe S, Lombard C, Baron D, Bekker LG, Nkala B, Ahmed K, et al. Tenofovir 1% vaginal gel for prevention of HIV-1 infection in women in South Africa (FACTS-001): a phase 3, randomised, double-blind, placebo-controlled trial. Lancet Infect Dis. 2018;18(11):1241-50. Epub 2018/12/07. doi: 10.1016/S1473-3099(18)30428-6. PubMed PMID: 30507409.

21.	Marrazzo JM, Ramjee G, Richardson BA, Gomez K, Mgodi N, Nair G, et al. Tenofovir-based preexposure prophylaxis for HIV infection among African women. N Engl J Med. 2015;372(6):509-18. doi: 10.1056/NEJMoa1402269. PubMed PMID: 25651245; PubMed Central PMCID: PMC4341965.

22.	Blue SW, Winchell AJ, Kaucher AV, Lieberman RA, Gilles CT, Pyra MN, et al. Simultaneous quantitation of multiple contraceptive hormones in human serum by LC-MS/MS. Contraception. 2018;97(4):363-9. Epub 2018/02/07. doi: 10.1016/j.contraception.2018.01.015. PubMed PMID: 29407362; PubMed Central PMCID: PMCPMC5840044.
