# Supplementary material for: Randomized, placebo controlled phase I trial of the safety, pharmacokinetics, pharmacodynamics and acceptability of a 90 day tenofovir plus levonorgestrel vaginal ring used continuously or cyclically in women: The CONRAD 138 study
Source: PLoS One. 2022 Oct 10;17(10):e0275794. doi: 10.1371/journal.pone.0275794 (PMC9550080; doi:10.1371/journal.pone.0275794)
Supplement: Attachment — Submitted filename: Response to Reviewers REV2 THURMAN 8 JUN 2022.docx [file pone.0275794.s010.docx]

Andrea Thurman, MD

Professor of Obstetrics and Gynecology

Eastern Virginia Medical School/CONRAD

601 Colley Ave, Norfolk, VA 23507

Email: [thurmaar@evms.edu](mailto:thurmaar@evms.edu), Phone: 210-380-5241

8 JUN 2022

**Dear Dr. Phillips, Editorial Team Members and Reviewers,**

Thank you for your thoughtful second review of our manuscript, PONE-D-21-23955R1, “Randomized, Placebo Controlled Phase I Trial of Safety, Pharmacokinetics, Pharmacodynamics and Acceptability of a 90 day Tenofovir Plus Levonorgestrel Vaginal Ring in Women”. We have addressed all of the additional reviewer’s comments and recommended edits below. We point to the page and line number(s) of the changes in a revised, tracked version of the manuscript. We hope that these changes make our manuscript acceptable for publication.

**Reviewer #1: Minor revisions: (Page numbers refer to those in the tracked changes version of revision 1.)**
1- Page 19, Line 8 and Page 25, Line 19: Clarify the phrase "exact statistical methods."

ANSWER: We have clarified that we mean the Fisher’s exact test, which is not impacted by sample size. This clarification is now on page 18, line 6 of the revision #2 tracked manuscript

2- Page 26, Line 4 and Page 29, Line 9: "Fisher's exact."

ANSWER: We have made this correction, on page 8, line 22 of the tracked second revision.

3- Tables 3, 4, 6, 7 and supplemental tables: Typically median, first and third quartiles are used to summarize non-normally distributed data.

ANSWER: We have edited Tables 3, 4, 6 and 7 to show medians, with the 25^th^ – 75^th^ interquartile ranges for these non-normally distributed data.

4- The abbreviation STD appears in the supplemental tables.

ANSWER: We have revised the Tables 3, 4, 6 and 7 to only include median and interquartile ranges and deleted mean and standard deviation for these non-normally distributed data. In Table 1, standard deviation is correctly abbreviated as SD.

Reviewer #2: • Congratulations by the improvement of the manuscript.
• Something that must be more obvious from the first reading is that you are comparing LVG/TNF ring vs placebo under TWO regimens of use (continuous vs cyclic), resulting in a 4-arms study. I’d suggest to include these ideas (2 products, 2 regimens) from the very beginning (title, abstract), etc.

ANSWER: We have added the dosing regimen (continuous versus cyclic) in the title of the manuscript. Even with this addition to the title, the title is less than the 250 character limit (204 characters). We also added the clarification that this is a 4 arm study to the abstract and introduction.

• ABSTRACT: Please, follow the structure of introduction, methodology, results, discussion and conclusion (but avoid the section titles).

ANSWER: We have added an introductory statement to the abstract so that now the abstract has the introduction, methodology, results, discussion and conclusions highlighted in the correct order. We have stayed under the 300 word abstract limit (299 words) with these changes.

• Page 6, Line 6: The study hereby presented (CONRAD 138)…….(to better distinguish both studies).

ANSWER: We agree and made edits to the introduction (page 6 of the tracked second manuscript revision) to distinguish the first-in-woman 14 day study (CONRAD 128) from the current 90 day full duration of treatment study (CONRAD 138).

• 7, 15-17: The quotation of some factors is not necessary: ….(e.g. ….infections).

ANSWER: We agree that since the STI screenings are listed in Supplementary Table 1 and the study protocol, we have taken out the specific listings of exclusionary infections (active HSV-2, *Neisseria gonorrhoeae, Chlamydia trachomatis, Trichomonas vaginalis*, HIV-1, and/or Hepatitis B). This deletion is found on page 7, lines 17 and 18.

• 9,3: Data of residual drug must be provided!!!!! If I’m not wrong, there are several comments to residual glycerin as adherence marker, but not residual drug. Data form LNG/TNF IVR must be provided, analyzed between regimens (continuous vs cyclic) and discussed if differences are seen.

ANSWER: Thank you for this question. We found an intriguing relationship between TFV release rate from the rings and the composition of the vaginal microbiota. Higher than expected in vivo release rates were observed in women with community state type IVA/B, polydisperse microbiota. We have reported these data in a separate manuscript [Thurman AR, Ravel J, Gajer P, Marzinke MA, Ouattara LA, Jacot T, Peet MM, Clark MR, Doncel GF. Vaginal Microbiota and Mucosal Pharmacokinetics of Tenofovir in Healthy Women Using a 90-Day Tenofovir/Levonorgestrel Vaginal Ring. Front Cell Infect Microbiol. 2022 Mar 8;12:799501. doi: 10.3389/fcimb.2022.799501. PMID: 35350436; PMCID: PMC8957918.] describing detailed correlations with microbial community states and individual bacteria such as *Prevotella bivia*, *Gardnerella vaginalis* and lactobacillus species (now reference 14 in the second revision). This finding has also been observed in a study of the TFV and TFV/LNG IVRs in Kenyan women (Dabee et al., submitted). Conversely, release rates were similar and not statistically different between rings used continuously or cyclically. We have now clarifications about the included a statement and referenced this paper in methods (pages 9 and 10 in the tracked second revision) and in the discussion section (page 52, lines 17 – 22).

Thank you again for your thoughtful review of our manuscript. We hope that the additional revisions will make the manuscript acceptable for publication in PLoS One.

Sincerely,

Andrea Thurman
