# Supplementary material for: Randomized, placebo controlled phase I trial of the safety, pharmacokinetics, pharmacodynamics and acceptability of a 90 day tenofovir plus levonorgestrel vaginal ring used continuously or cyclically in women: The CONRAD 138 study
Source: PLoS One. 2022 Oct 10;17(10):e0275794. doi: 10.1371/journal.pone.0275794 (PMC9550080; doi:10.1371/journal.pone.0275794)
Supplement: Attachment — Submitted filename: Response to Reviewers REV4 THURMAN 11 JUL 2022.docx [file pone.0275794.s011.docx]

Andrea Thurman, MD

Professor of Obstetrics and Gynecology

Eastern Virginia Medical School/CONRAD

601 Colley Ave, Norfolk, VA 23507

Email: [thurmaar@evms.edu](mailto:thurmaar@evms.edu), Phone: 210-380-5241

11 JUL 2022

**Dear Jianhong Zhou, Editorial Team Members and Reviewers,**

Thank you for your thoughtful third review of our manuscript, PONE-D-21-23955R2, “Randomized, Placebo Controlled Phase I Trial of Safety, Pharmacokinetics, Pharmacodynamics and Acceptability of a 90 day Tenofovir Plus Levonorgestrel Vaginal Ring in Women”. We have addressed all of the additional reviewer’s comments and recommended edits below. We point to the page and line number(s) of the changes in a revised, tracked version of the manuscript. We hope that these changes make our manuscript acceptable for publication.

This manuscript was originally submitted to PLoS One on 29 JUL 2021. We thank the PLoS One team for continuing to solicit reviewers and academic editors for our work. This appears to be a third set of reviews, with an additional new reviewer and a third academic editor. As expected, there are some contradictions between what previous reviewers wanted added or deleted and the comments of the current reviewers, in particular the new reviewer. We will now delete additions, as recommended by Reviewer 2, which were previously requested in the first and second reviews of this manuscript.

Reviewers' comments:

Reviewer #1: (No Response)

Reviewer #2: OVERVIEW
Please adapt the abstract in accordance with below suggested changes.

Response: Abstract adapted as per the suggestions below

Avoid abbreviations in titles (including section titles)

Response: We have removed all abbreviations from titles and sub-titles.

Discuss with the Editor the alternative of splitting the manuscript in 2 different but related papers.

Response: Our research group originally submitted this manuscript to PLoS One on July 29, 2021. This manuscript represents the main findings of the CONRAD 138 study. It has gone through multiple academic editors and reviews. In fact, during the year-long PLoS One review, we published a second, related manuscript of the relation between the vaginal microbiota and pharmacokinetics of tenofovir [1]. We very much want to publish the main findings of the CONRAD 138 study and think the way they are presented in the manuscript has conceptual unity. Therefore we prefer not to split the data and manuscript into two related paper. Furthermore, two previous sets of academic editors and reviewers were agreeable to present these data as a single manuscript.

MAJOR COMMENTS
The same order of objectives must be followed through the text. I understand that should be the one stated on the title: Safety, PK, PD, Acceptability. Then follow it in all sections: Pg5,Ln 8-9; Methods (Safety-> PK -> PD -> Acceptability), Results, Discussion and Conclusions. Safety must be always the first since it is the primary outcome.

Response: We have changed the order in the methods and results section to reflect the objectives of the study: Safety, Pharmacokinetics, Pharmacodynamics and Acceptability (effect on the menstrual cycle).

A review of the manuscript by an expert in drug safety / pharmacovigilance is highly recommended. Some of the statements from authors should be carefully reassessed.

Response: The manuscript has been reviewed by Dr. Jill Schwartz, MD, and Dr. Mark Marzinke, PhD, who currently are in charge of pharmacovigilance and pharmacology, respectively, at a large clinical contract research organization and John Hopkins School of Medicine. We have revised terminology regarding TEAE and clarified assessment and recording of all AEs as requested. The study was performed, and therefore reviewed and independently monitored, under an IND (#118,510) from the USFDA.

MINOR COMMMENTS
Pg1, Ln1: Typo error “59”

RESPONSE: The additional “59” in the title was removed

Pg4, Ln19-22: The manuscript is long and this paragraph does not add relevant information, please delete.

RESPONSE: We removed this text. Of note, during the initial review of this manuscript, a previous PLoS One reviewer requested these additional details.

Pg6,Ln14: Please list exclusion criteria as you did inclusion. When they were evaluated is described elsewhere. Same for the next paragraphs, the visits are already described in S1 Table.

RESPONSE: We added the exclusion criteria on page 6, lines 12 - 16.

Pages 6,9. Randomization is described later.

RESPONSE: We have deleted the details of the randomization explanation from pages 6 and 7, as this is repeated in the randomization section.

7.4 Blood extraction timing is part of the PK methods.

RESPONSE: We have deleted lines 4 – 8 of page 7, as the timing of the PK blood draws after initial IVR insertion is explained in the PK Methods section.

7.8 Confirmation of anovulatory effect is part of the PD.

RESPONSE: We have consolidated the methods we used for assessing levonorgestrel PD in to the Methods section (pages 16 and 17)

10.5 If “The unit dose for the IVRs…” is true, then the placebo also release TFV and LNG…!!!! To avoid this type of mistake, describe first the similarities (manufacturing place, physical appearance) and then the drug content of the active product.

RESPONSE: We have corrected the errors in explaining the active TFV/LNG IVR versus the placebo IVR on page 10, lines 7 – 21.

11.2 In accordance with Good Pharmacovigilance Practice, all AE must be recorded, and subsequently classified. This statement must be included, not only refer to TE (which of course are the relevant).

RESPONSE: As clarified in the Methods section, page 11, AEs were collected at each visit, starting with the enrollment visit, visit 3. AEs noted during screening (visits 1 and 2) would be medical history AEs. We clarified in the methods section that AEs were collected at each of the 32 visits, starting with visit 3 and were coded with the appropriate MDR code.

11. Delete “or …reaction”

RESPONSE: We deleted this phrase on page 11, line 18. Of note, this verbage was a requested addition during the first round of reviews.

12. As above requested, follow the order of sections.

RESPONSE: We have re-ordered the methods section to explain safety (both clinical and sub-clinical safety), PK, PD and acceptability assessments.

12. 14 CVL abbreviation.

RESPONSE: CVL (cervico-vaginal fluid lavage) is now defined on page 13, line 1.

12.22 And CFR compliant also, I hope!!

RESPONSE: Yes, CFR compliance was added, now on page 17, line 16.

16.22 Contraception was not investigated, women followed abstinence !!!

RESPONSE: We agree, and this is why the sub-title is pharmacodynamics surrogate. We have added that potential contraceptive efficacy was modeled by several surrogates to make it clear that we are not directly evaluating contraceptive efficacy or tracking pregnancy.

17.3-5 This limitation of the study must be transferred to Discussion section.

RESPONSE: We have deleted the verbage from the Statistical Analyses section (page 18, lines 10 – 12) and transferred this to the Discussion section, page 56, line 13.

17.8 Delete (V3…)

RESPONSE: Deleted

17.9 Before this last paragraph, methodology to assess acceptability is missing.

RESPONSE: We have moved the acceptability assessments to just prior to the sample size and statistical analyses section (page 17, lines 15 – 21) to keep the same order of Safety, PK, PD and Acceptability.

17.19 “No participant…. Is a Safety result, no allocation of population.

RESPONSE: We clarified that safety results are reported in the evaluable population. Page 18, lines 16 – 19.

31.1 No previous mention to “expulsions” in objectives, nor in methods…. Actually, no mention of expulsion in this paragraph beyond the title….

RESPONSE: The expulsions and adherence results section describes the study population’s compliance with the study product. We have therefore moved these results to the study population section, page 24.

31.1 If you consider Adherence as part of Acceptability assessment, explain and merge. If you consider as part of follow-up, then must be described before main results: Safety > Pk>Pd>Accept.

RESPONSE: We agree that adherence is part of the follow up of the study population. We have therefore moved these results to the study population section page 24.

45.5 A first paragraph of wrap-up is usually recommended at the beginning of the Discussion.

RESPONSE: We have added a wrap up sentence which summarizes PK and PD data to the beginning of the Discussion, page 50.

46.9 Cannot considered out of caution. Taking in consideration the occurrence of genital ulcers in this and in Keller’s studies, this AE must be considered as a serious concern to be carefully evaluated within the present and future clinical development. It would probably be considered as a risk by Health Authorities and probably, additional measures of safety surveillance will be required.

RESPONSE: We have now removed this verbage.

46.12-16 The two potential causes of ulcers are here confounded: 1-drug effect over the epithelium; 2-ring’s mechanical effect. COMMENT: both must be investigated in future trials.

RESPONSE: We agree and have cited drug effect (reference 34) and mechanical effect, as we clarified that 90% of patients enrolled in this trial were sexually active.

47.8-9 SERIOUS INCONSISTENCY: TEAE means Treatment – Emergent……therefore, by definition, it is considered related with the treatment !!!!!!!!!!!!!!!!!!!!!!!!!!!!!!

RESPONSE: To add clarity, we have changed the term treatment emergent adverse events (TEAEs) to adverse events (AEs). There are treatment-emergent and treatment-related AEs. We considered TEAEs those AEs occurring during treatment, whether they were related to treatment or not. As noted in the Methods section, adverse events were collected at every visit after enrollment. Each AE was graded as to severity and relatedness to study product or study procedures. Not all AEs that occur during a trial are related to the study product or procedure. All AEs were monitored by an independent data monitor and reported to the FDA as part of the Clinical Study Report.

We hope that these third round of changes makes our manuscript acceptable for publication in PLoS One.

Sincerely

Andrea R. Thurman MD

1.	Thurman AR, Ravel J, Gajer P, Marzinke MA, Ouattara LA, Jacot T, et al. Vaginal Microbiota and Mucosal Pharmacokinetics of Tenofovir in Healthy Women Using a 90-Day Tenofovir/Levonorgestrel Vaginal Ring. Front Cell Infect Microbiol. 2022;12:799501. Epub 20220308. doi: 10.3389/fcimb.2022.799501. PubMed PMID: 35350436; PubMed Central PMCID: PMCPMC8957918.
