# Supplementary material for: Randomized, placebo controlled phase I trial of the safety, pharmacokinetics, pharmacodynamics and acceptability of a 90 day tenofovir plus levonorgestrel vaginal ring used continuously or cyclically in women: The CONRAD 138 study
Source: PLoS One. 2022 Oct 10;17(10):e0275794. doi: 10.1371/journal.pone.0275794 (PMC9550080; doi:10.1371/journal.pone.0275794)
Supplement: Attachment — Submitted filename: Response to Reviewers REV5 THURMAN 22 AUG 2022.docx [file pone.0275794.s012.docx]

Andrea Thurman, MD

Professor of Obstetrics and Gynecology

Eastern Virginia Medical School/CONRAD

601 Colley Ave, Norfolk, VA 23507

Email: [thurmaar@evms.edu](mailto:thurmaar@evms.edu), Phone: 210-380-5241

22 AUG 2022

**Dear Vanessa Carels, Editorial Team Members and Reviewers,**

Thank you for your thoughtful fourth review of our manuscript, PONE-D-21-23955R3, “Randomized, Placebo Controlled Phase I Trial of Safety, Pharmacokinetics, Pharmacodynamics and Acceptability of a 90 day Tenofovir Plus Levonorgestrel Vaginal Ring in Women”. We have addressed all of the additional reviewer’s comments and recommended edits below. We point to the page and line number(s) of the changes in a revised, tracked version of the manuscript. We hope that these changes finally make our manuscript acceptable for publication.

**REVIEWER COMMENTS:**

Reviewer #2: Thanks to the authors for this new effort and congratulations for the improvement. The questions were appropriately addressed and the present version, with only minor suggestions would be acceptable.

TITLE: Apologize for adding this suggestion now, but previous topics were of higher priority. Please, consider to add the name of the study at the end of the title: “Randomized …. used Continuously or Cyclically in Women: The CONRAD 138 Study”.

RESPONSE: We have added “The CONRAD 138 Study” to the title.

Pg.5, Ln.19: I assume that a contraceptive method was recommended, since sexual activity was allowed and there is a placebo arm. A sentence must be included in this section.

RESPONSE: We included that participants could not be at risk of pregnancy due to the consistent use of condoms, sterilization or heterosexual abstinence on page 6, lines 10 – 12.

Pg.6, Ln.13: “(BV)” is not necessary, since it is not used later.

RESPONSE: This abbreviation has been deleted on page 6, line 15.

10, 15: Please, include the method, algorithm or scale used to determine relationship to AE to study product (as well done in the previous line specifying the DAIDS for severity).

RESPONSE: We clarified on page 10, line 15 that the relationship of the treatment emergent adverse event to study product or study procedures was graded as related or not related. This process included assignment of relatedness by the clinical site PI and confirmation of classification by the Sponsor’s Medical Director. We did not include other sub-divisions of this grading scale such as possibly related, probably related, etc.

46, 9-10: Sentence unclear, please review.

RESPONSE: We have re-written this sentence, now on page 46, lines 9 – 12 to state, “Twelve of 18 TFV/LNG IVR continuous dosing users reported mild or moderate AEs versus 15 of 18 TFV/LNG IVR cyclic dosing users. While this difference was statistically significant, we do not believe that it is clinically relevant, as the difference is small and AEs were mostly mild and unrelated to product use.”

Pages 50-52: Considering this discussion and having in mind HIV protection objective, would you recommend the continuous or the intermittent use? Or this question is still unclear and must be elucidated in future studies? A sentence in the Conclusions section would be appropriate.

RESPONSE: Our data appear to support a preferred recommendation for continuous IVR use, as this regimen shows a more consistent PK/PD profile. We have added clarification to the discussion section of LNG PK (page 49, lines 15 - 20) that although we do not know if the decreases in serum LNG concentration with IVR removal would ultimately impact contraceptive efficacy, the fact that interrupted use does not offer an advantage over continuous use in menstrual bleeding patterns, supports continuous use. We have also added a statement to the conclusion section, page 54, lines 5 - 10 stating that “Because cyclic dosing did not offer an advantage in the menstrual bleeding pattern over continuous use, our data currently would support a preferred recommendation of continuous IVR use, as this regimen shows a more consistent PK/PD pattern. Long-lasting protective levels of TFV-DP in tissue, however, would also support ring removal for short periods.”

Again, we thank the team for their thoughtful review and hope that this 4^th^ revision makes the manuscript acceptable for publication in PLoS One.

Sincerely,

Andrea Thurman, MD
